# Factors Associated with Early Deterioration after Spontaneous Intracerebral Hemorrhage: A Systematic Review and Meta-Analysis

**DOI:** 10.1371/journal.pone.0096743

**Published:** 2014-05-08

**Authors:** Adrian V. Specogna, Tanvir C. Turin, Scott B. Patten, Michael D. Hill

**Affiliations:** 1 Department of Community Health Sciences, Faculty of Medicine, University of Calgary, Calgary, Alberta, Canada; 2 Department of Clinical Neurosciences, Faculty of Medicine, University of Calgary, Calgary, Alberta, Canada; St Michael's Hospital, University of Toronto, Canada

## Abstract

**Background and Purpose:**

Spontaneous intracerebral hemorrhage (ICH) is a devastating form of stroke with a poor prognosis overall. We conducted a systematic review and meta-analysis to identify and describe factors associated with early neurologic deterioration (END) after ICH.

**Methods:**

We sought to identify any factor which could be prognostic in the absence of an intervention. The Cochrane Library, EMBASE, the Global Health Library, and PubMed were searched for primary studies from the years 1966 to 2012 with no restrictions on language or study design. Studies of patients who received a surgical intervention or specific experimental therapies were excluded. END was defined as death, or worsening on a reliable outcome scale within seven days after onset.

**Results:**

7,172 abstracts were reviewed, 1,579 full-text papers were obtained and screened. 14 studies were identified; including 2088 patients. Indices of ICH severity such as ICH volume (univariate combined OR per ml:1.37, 95%CI: 1.12–1.68), presence of intraventricular hemorrhage (2.95, 95%CI: 1.57–5.55), glucose concentration (per mmol/l: 2.14, 95%CI: 1.03–4.47), fibrinogen concentration (per g/l: 1.83, 95%CI: 1.03–3.25), and d-dimer concentration at hospital admission (per mg/l: 4.19, 95%CI: 1.88–9.34) were significantly associated with END after random-effects analyses. Whereas commonly described risk factors for ICH progression such as blood pressure, history of hypertension, and ICH growth were not.

**Conclusions:**

This study summarizes the evidence to date on early ICH prognosis and highlights that the amount and distribution of the initial bleed at hospital admission may be the most important factors to consider when predicting early clinical outcomes.

## Introduction

Spontaneous intracerebral hemorrhage (ICH) is widely considered to be the most devastating form of stroke. Up to half of sufferers may die from ICH and the majority of survivors will remain permanently disabled [1], [2]. ICH progresses rapidly and the majority of those who deteriorate neurologically, will do so within the first few days [3], [4].

Several studies have investigated hypertensive treatments as well as other potential therapies for ICH; many of which were designed to identify and treat ICH progression [5], [6]. Despite this, there is currently no approved, effective therapy. Although the exact mechanism for treatment failures in clinical studies is currently unknown, one potential reason may be that we truly do not understand what factors affect early deterioration and thus have been unable to target appropriate mechanisms of injury, or control for important confounders in clinical studies [7]. We conducted a systematic review and meta-analysis to identify and describe factors associated with early neurologic deterioration (END) after ICH; with a goal to summarize the current state of knowledge of early ICH prognosis and guide future clinical research.

## Methods

### Definitions

Ethical approval was not required for this study. The population under study was adults with spontaneous ICH treated with standard medical care. Authors must have stated they included ‘primary’ or ‘spontaneous’ ICH patients or at least stated that they excluded patients with ICH due to trauma, tumors/malignancy, aneurysm, anticoagulant use, or arteriovenous malformations. Further, we excluded studies which included patients who received surgery prior to the assessment of deterioration, and we also excluded studies where all patients received a specific medical therapy. Our specific inclusion and exclusion criteria are described in Table 1.

**Table 1:** Review inclusion and exclusion criteria.

Inclusion Criteria
Studies of spontaneous/primary ICH patients
Studies of humans
Studies of adults (≥ 18 years of age)
Studies which measured any prognostic factor
Studies which measured END
Studies published in a peer reviewed journal
Studies in any language with an English abstract available
Primary studies of any design
Exclusion Criteria
A full-text copy of the study could not be obtained
A measure of risk (e.g. OR, RR, HR) for spontaneous/primary, adult ICH patients could not be estimated
Patients received an experimental or specific medical therapy or it was unclear
Patients received a surgical intervention before END was measured or it was unclear
The prognostic factor was not defined
END was not defined
The reliability of the measure used to define END was poor or unclear in the literature
The timing of the measurement of the prognostic variable or END was not defined or unclear

END denotes early neurologic deterioration.

We sought to identify any factor which may be associated with deterioration. Specifically, a prognostic factor was defined as any specific biological characteristic or indicator of a specific biological characteristic that could be measured early after ICH and could be prognostic in the absence of an intervention [8]. We only considered prognostic factors which were reported by two or more studies, using the same definition, and where a similar measure of risk could be estimated and compared [9].

**Figure 1:**
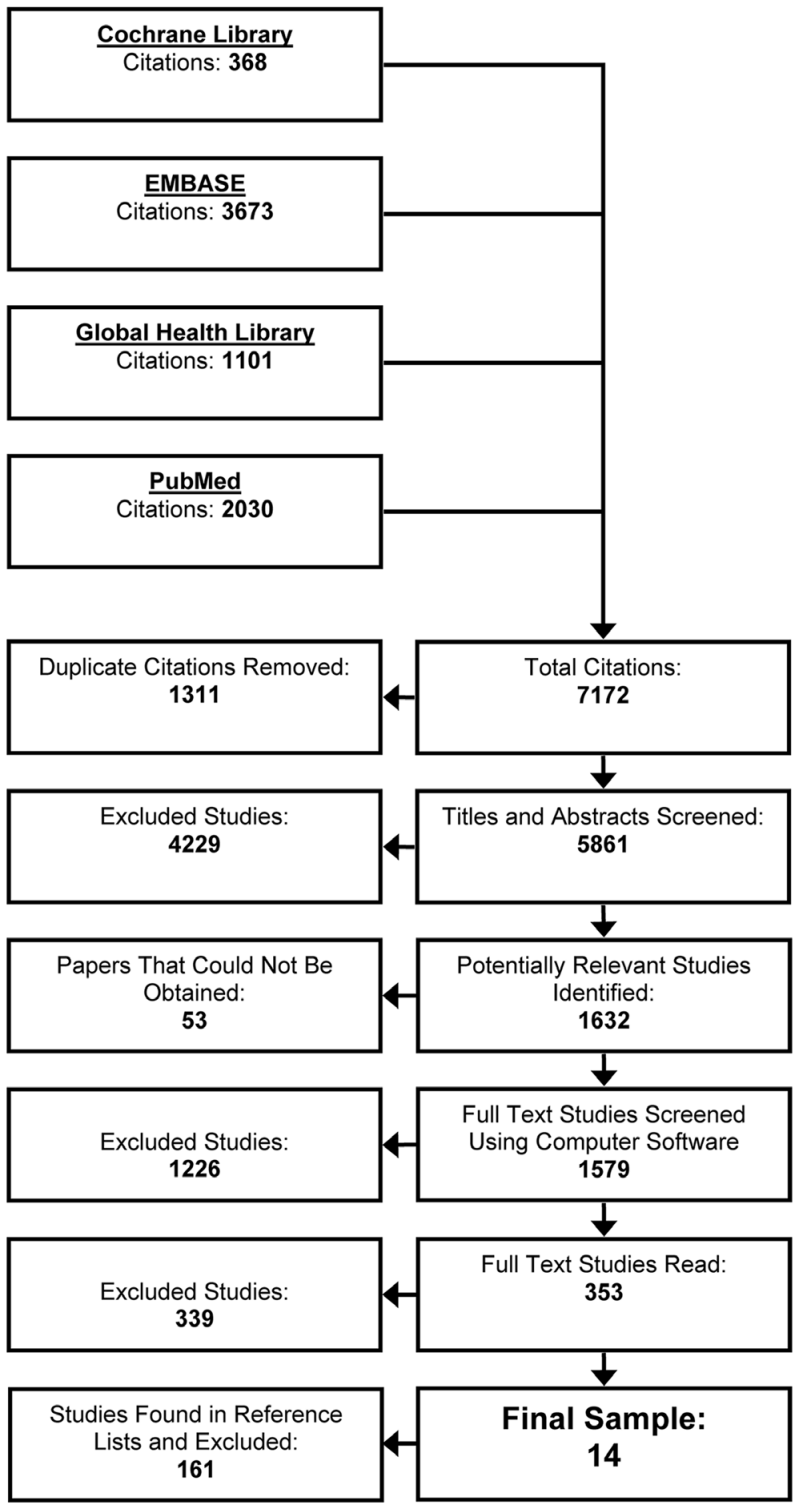
Literature search flow. Studies were excluded because they didn't appear to measure deterioration or adult ICH during abstract screening (n = 4229), full-text versions could not be obtained (n = 53), none of the >3000 words or phases for END could be found in the full-text (n = 1226), authors did not measure END in adult ICH patients (n = 264), a measure of risk could not be estimated (n = 47), all patients received an experimental or specific medical therapy or it was unclear (n = 12), patients received a surgical intervention before END was measured or it was unclear (n = 44), the prognostic factor was not defined (n = 1), END was not defined (n = 6), the reliability of the measure used to define END was poor or unclear (n = 1), the timing of the measurement of the prognostic variable or END was not defined or unclear (n = 109), the study was the only study to describe a specific prognostic factor (n = 1), or the study did not describe new data (n = 15).

The primary outcome was END. Several studies have demonstrated that, of those patients who deteriorate after ICH, the majority will do so within the first week [3], [4]. Thus, we felt it was reasonable to define END as death, or worsening on a reliable outcome scale within seven days after onset; since assessing deterioration beyond when it is likely to occur may introduce confounding into risk estimates. Death was defined as all-cause mortality and a reliable neurologic outcome scale was one that measured neurologic impairment according to the World Health Organization's International Classification of Functioning, Disability and Health model and had been reported in at least one previous study to have good reliability in patients with stroke. The search flow is described in Figure 1.

### Search

In consultation with a librarian and experts in stroke research, a list of over 300 unique key words and phrases were created for intracerebral hemorrhage and deterioration. These terms were searched in four databases; The Cochrane Library, EMBASE, The Global Health Library, and PubMed from January 1^st^, 1966[9] to December 16^th^, 2012.

Any primary study published in a peer-reviewed journal in any language was eligible for inclusion provided an English abstract was available. A primary study was defined as a study which was a randomized clinical trial, cohort study, case-control study, case-series, quasi-experimental, or any study which collected and reported new, unique data.

The titles and abstracts of citations identified in the databases were screened by one reviewer who had a PhD in epidemiology and expertise in stroke research (A.V.S). Studies were retained for further review if they examined adult ICH patients, they measured or reported death or deterioration at any time during follow-up, or the reviewer was uncertain of any of the above.

A full-text copy of studies which met any of the three aforementioned criteria during the abstract screening process was obtained from our university holdings or Docline; a multinational interlibrary document delivery service. If articles could not be found in any of these sources, it was assumed to be irrelevant and excluded. A list of over 3000 unique words and phrases for END was developed. All obtained papers were searched using computer software (PDF Converter Professional version 8.0 [10]) for any of these keywords or phrases. Papers were retained for further review if they contained at least one of these words or phrases. Non-English articles were translated to English prior to screening or screened by a native speaker. The full electronic search strategy with all keywords and phrases is available in Data Supplement s1.

Three hundred and fifty-three studies were selected for further review by one of two reviewers (A.V.S. or T.C.T.). Both reviewers had PhD degrees in epidemiology and expertise in stroke research. A random sample of these studies (n = 236) were read by both reviewers to determine the reliability of human screening. Agreement between raters was excellent with an unweighted interrater kappa of 0.86. Disagreements between raters were resolved by consensus. Consensus was reached for each study. The reference lists of included studies were searched for missing studies. No additional studies were found and no studies which were previously excluded during computer screening were found to be relevant. Data was extracted from these studies by one reviewer (A.V.S.). An attempt was made to contact the authors to provide individual patient data for further analysis. Too few authors responded to make these analyses meaningful.

### Statistical Methods

Studies were combined using random-effects models (DerSimonian and Laird [11]) in the absence of substantial heterogeneity. It was reasonable to use random-effects models since some heterogeneity across studies was expected due to differences in when END was measured throughout the first week, differences in treatment strategies, and differences in patient characteristics across studies.[12] We used the Cochrane Q test and stratified analysis to investigate and describe heterogeneity across studies, and investigate the impact of factors related to study quality on combined estimates within each factor comparison [12], [13]. The I^2^ statistic [13] was used to quantify statistical heterogeneity, and patient inclusion and exclusion criteria were assessed to determine clinical heterogeneity between studies.

For all analyses, effect estimates (hazard ratios (HR), risk ratios (RR), odds ratios (OR)) or data provided by the authors in the original publication were used. To remain consistent with previous systematic reviews of ICH [9], all results were reported as ORs. Hazard ratios were assumed to equal RRs, and ORs were assumed to approximate RRs provided the proportion of patients who experienced END was less than 15% in the respective study [14], or the proportion of patients without the prognostic factor who experienced END was known, so the OR could be corrected to approximate an RR [15]. If the authors reported sufficient data to calculate multiple estimates of the same associations, then the ratio estimates reported by the authors were always used over estimates calculated from the raw data; provided the direction of the estimate was described. Also, adjusted estimates were always used over unadjusted estimates if available. If raw data were used then ORs were estimated from categorical and continuous data using methods previously described [13]. If continuous data were presented as a median it was assumed the data were skewed and thus not included in any analyses. All analyses were conducted using Stata statistical software (version 12) [16] assuming alpha was equal to 0.05.

## Results

**Figure 2:**
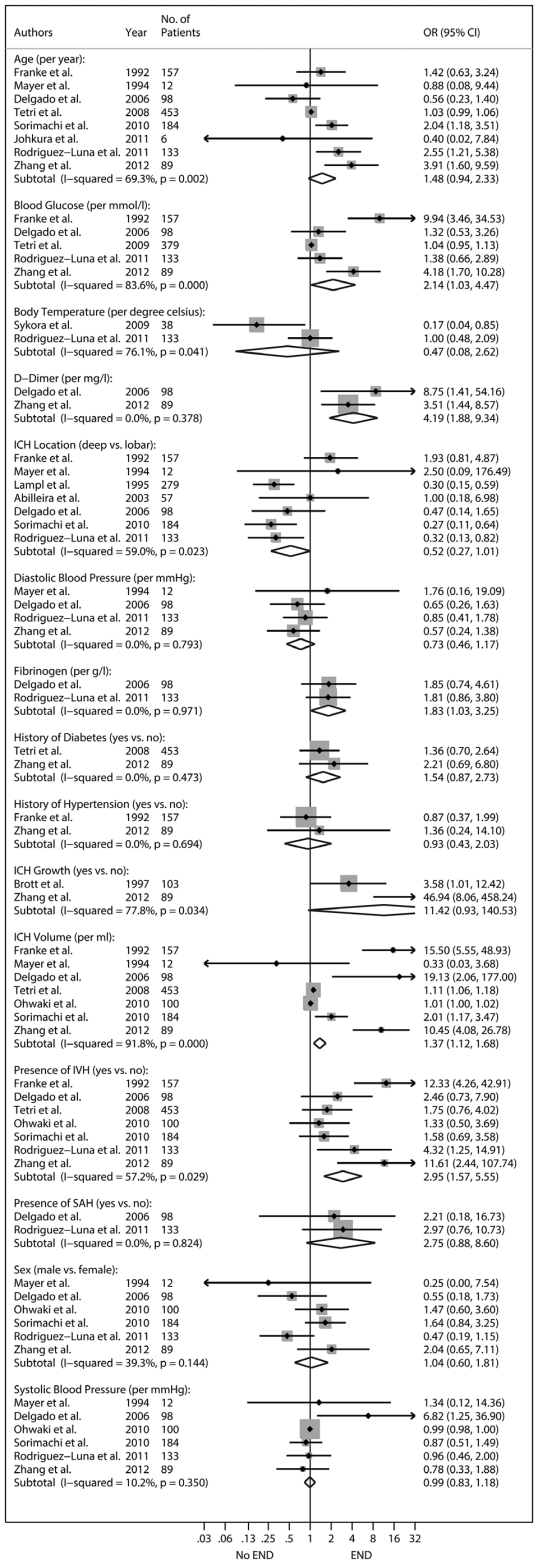
Prognostic factors identified and their association with early neurologic deterioration (END). Effect estimates are reported as odds ratios (OR) with 95% confidence intervals (CI). Subtotal denotes the overall combined random-effects OR for each given factor. Point estimates lying to the right of one suggest an increased odds of END per one unit increase in the reported units. Dichotomous factors are coded and reported as 1 vs. 0 (e.g. deep location = 1, lobar = 0). Confidence intervals (horizontal lines encompassing the point estimates) which cross one suggest no association. IVH denotes intraventricular extension. SAH denotes subarachnoid extension. The boxes surrounding the point estimates represents the relative weight each study has within the factor comparison; such that larger boxes represent greater weight in the overall combined estimates.

Fifteen factors were identified across 14 studies. A description of these studies is provided in Table 2. A summary of the results is provided in Figure 2. Two studies with similar samples were identified [17], [18]. In the cases of the age, ICH volume, and IVH analyses, both studies were eligible for inclusion in the same analysis. In these cases, only the larger study was used [18]. Deep location was defined as structures associated with basal ganglia or thalamus. All factors were measured at hospital admission except ICH growth. ICH growth was defined as a >33% increase in ICH volume from baseline to follow-up.

**Table 2:** Characteristics of included studies.

Authors	Design	No. of Patients	Definition of END	Patient Exclusion Criteria
Mayer et al [3].(1994)	Prospective Cohort	12	Death	Patients with trauma, tumor, ICH related to aneurysm or AVM, multiple hemorrhages, primary IVH.
Delgado et al [23].(2006)	Prospective Cohort	98	Death	Patients with ICH related to AVM, impaired coagulation, receiving anticoagulant therapy, head trauma, hemorrhagic infarction, tumoral bleedings, history of deep vein thrombosis.
Brott et al [25].(1997)	Prospective Cohort	103	Change in GCS	Patients with ICH due to trauma, ruptured aneurysm, AVM, tumor, receiving anticoagulant therapy, primary SAH, primary IVH.
Tetri et al [18].(2008)	Retrospective Cohort	453	Death	Patients with ICH due to brain tumor, aneurysm, AVM, hematological malignancy, coagulation disorder, head trauma, patients admitted to departments other than neurology, patients living outside the hospital catchment area.
Rodriguez-Luna et al [29].(2011)	Prospective Cohort	133	Change in NIHSS or Death	Patients with unknown time of onset, GCS = 8.
Sorimachi et al [30].(2010)	Retrospective Cohort	184	Change in NIHSS	Patients admitted > 24 hours after onset, patients not receiving blood pressure control or antifibrinolytics, patients without a follow-up CT scan, ICH due to aneurysm, AVM, cavernous hemangioma, moyamoya disease, tumor, or cerebral angiitis.
Zhang et al [31].(2012)	Case-Control	89	Change in NIHSS	Patients with a history of neurological disease, head trauma, receiving antiplatelet or anticoagulant medication, other systemic diseases including uremia, liver cirrhosis, malignancy, chronic heart or lung disease (with the exceptions of diabetes mellitus and hypertension), patients without copeptin measurements.
Franke et al [35].(1992)	Prospective Cohort	157	Death	Patients with aneurysmal bleeding.
Lampl et al [36].(1995)	Prospective Cohort	279	Death	Patients with recurrent ICH, previous stroke, AVM, brain tumors, SAH, receiving anticoagulant therapy.
Abilleira et al [37].(2003)	Prospective Cohort	57	Change in CNS	Patients with infection, inflammatory or malignant diseases, receiving immunosuppressive treatment, AVM, aneurysms, tumors, hemorrhagic infarctions, coagulopathies, IVH.
Tetri et al [17]. (2009)	Retrospective Cohort	379	Death	Patients with ICH due to brain tumor, aneurysm, AVM, hematological malignancy, coagulation disorder, head trauma, patients admitted to departments other than neurology, patients living outside the hospital catchment area, patients without glucose or blood pressure measurements.
Sykora et al [38].(2009)	Prospective Cohort	38	Change in NIHSS	Patients with previous history of stroke, atrial fibrillation, myocardial infarction, diabetes mellitus, chronic renal failure, medical conditions known to affect autonomic functions, coagulopathy, receiving anticoagulant therapy, tumor, trauma, ICH due to cerebral venous thrombosis, requiring antihypertensive therapy, requiring cardiovascular active treatment at the time of admission, treated with osmotherapy.
Ohwaki et al [39].(2010)	Retrospective Cohort	100	Change in GCS or MMT	Patients with aneurysm, AVM, amyloid angiopathy, brain death shortly after admission, receiving sedative therapy (if interfered with neurological assessment), patients transferred out of hospital within 24 hours, admission GCS = 3.
Johkura et al [40].(2011)	Retrospective Cohort	6	Death	Not stated.

NIHSS denotes National Institutes of Health Stroke Scale, GCS denotes Glasgow Coma Scale, CNS denotes Canadian Neurological Scale, MMT denotes Manual Muscle Testing, AVM denotes arteriovenous malformation/vascular malformations, IVH denotes intraventricular hemorrhage, SAH denotes subarachnoid hemorrhage, END denotes early neurologic deterioration.

Random-effects analyses suggested ICH volume (combined OR per ml higher: 1.37, 95%CI: 1.12–1.68), presence of intraventricular hemorrhage (combined OR for having IVH: 2.95, 95%CI: 1.57–5.55), glucose concentration (combined OR per mmol/l higher: 2.14, 95%CI: 1.03–4.47), fibrinogen concentration (combined OR per g/l higher: 1.83, 95%CI: 1.03–3.25), and d-dimer concentration (combined OR per mg/l higher: 4.19, 95%CI: 1.88–9.34) at hospital admission were significantly associated with END. There was evidence of statistical heterogeneity across studies that assessed ICH volume, presence of IVH, and glucose concentration as well as age, body temperature, ICH location, and ICH growth.

Since the number of studies in each factor comparison was low overall (<20) [19], we used stratified analysis to examine whether important study characteristics changed the interpretation of the overall combined OR estimate in all factor comparison regardless of observed statistical heterogeneity. Studies were stratified based on estimate adjustment (adjusted vs. unadjusted), type of measure used to define END (death vs. neurologic scale), blinding of prognostic factor measurement (yes vs. no), whether any patients received medical treatments for blood pressure, intracranial pressure, body temperature, deep vein thrombosis, glucose, iron, or abnormal clotting; provided this data were reported. Neither estimate adjustment, blinding, nor stratification based on treatment changed the interpretation of the overall combined OR for any prognostic factor investigated. With regard to patient age, studies which defined END based on a neurologic outcome scale, were more likely to report that older age was significantly associated with END compared to those that defined END as death only (combined OR for older age using a neurologic outcome scale: 2.55 (95% CI; 1.39–4.68). Since neurologic scales are subjective, evaluator bias may be playing a role in this finding. Regardless, removing these studies did not change the interpretation of the overall combined OR (combined OR for older age using death to define END: 1.03 (95% CI; 1.00–1.07), however it may have described some of the observed statistical heterogeneity in this comparison.

Since there were less than 20 studies [19], we did not investigate publication bias using formal statistical methods. Implicit in these methods is the assumption that there is an association between study size and effect size or significance [13]. We found no significant association between study size and effect size (linear regression coefficient for larger study size: 0.193, 95%CI; −0.111 to 0.497), and no significant association between study size and the significance of the effect size (yes vs. no) (OR for larger study size: 1.64, 95%CI; 0.83 to 3.26) across all effect estimates reported.

## Discussion

After combining the published literature to date on ICH, we found evidence to suggest there may be a significant association overall between END and blood glucose, fibrinogen, and d-dimer concentrations, as well as ICH volume, and presence of IVH. With regard to blood glucose specifically, our observations are consistent with a previous review with a longer follow-up interval [20]. Although it is possible that the elevation of blood factors such as glucose, fibrinogen, and d-dimer after ICH may be indicative of a stress reaction due to severe hemorrhage, and thus not the cause of deterioration per se [21]–[23], the case for a causal interaction between higher hemorrhage volume or IVH on neurologic deterioration may be stronger [24].

This study suggests that for each millilitre increase in hemorrhage volume at hospital admission the odds of END increases by 37%, and those with IVH are almost three times as likely to deteriorate within the first week compared to those without IVH. The majority of studies to date suggest that ICH volume and IVH are positively associated with END, but combined analyses suggested statistical heterogeneity overall despite the larger sample sizes in these comparisons. Important study characteristics such as estimate adjustment, measures used to define END, blinding, and treatments used did not explain all of this statistical heterogeneity. Thus, our review illustrates the high variability of ICH cases across different centers and highlights the need to include large sample sizes in future studies to ensure sufficient power is reached.

Some have suggested that the presence of IVH may have confounded the observation of treatment effects in previous clinical studies of ICH therapy [7]. This review supports this theory and suggests that the presence of IVH, as well as ICH volume at admission may be associated with END, and thus should be investigated as confounders in future studies which measure END.

It has been demonstrated in several studies that an ICH hematoma continues to grow for some time during the acute period [25]. Thus, it has been widely accepted that hematoma growth during the acute period is perhaps one of the most clinically significant risk factors for neurologic deterioration [26]. Despite this, some have suggested that the magnitude of the initial ICH injury at hospital admission may be more important in predicting clinical outcomes than the physical ways ICH progresses after patients arrive [27]. Our findings on admission ICH volume and presence of IVH are inline with this theory, such that larger, diffuse bleeds at hospital admission were associated with early deterioration, and factors commonly thought to be related to ICH progression, by their proposed relationship to ICH growth, such as high blood pressure, were not. Specifically, nearly all of the studies we included which examined blood pressure or history of hypertension showed no association. Several studies were included in the blood pressure comparisons, the estimates were not significantly affected by differences in study characteristics, the precision was high in the overall combined OR, and there was no evidence of statistical heterogeneity. The combined estimate for ICH growth was also not significant, although this estimate should be viewed with caution. Although we felt it was reasonable to combine studies in the hemorrhage growth analysis based on their clinical characteristics, the estimates for both studies was highly variable and thus this factor warrants further investigation.

It is reasonable to suggest that an ICH grows initially after onset, and this growth may result in damage to brain tissue and larger hematomas. However, the results of this study support the theory that the damaging effects of high blood pressure or growth may be apparent well before patients arrive at hospital, perhaps minutes after ICH, and thus perhaps treating high blood pressure or growth early after hospital admission may not improve early outcomes [6]. In any case, this review provides evidence that it may be reasonable to consider conducting future studies of treatments which focus on the early reduction of both primary ICH volume and intraventricular volume. Such studies could focus on developing safer surgical techniques to reduce hematoma volume, since it appears that the extent of the initial bleed at hospital admission, whether it be in the brain tissue or ventricles, predicts deterioration early after ICH in various groups of ICH patients.

Several studies were not included in some factor comparisons, including hemorrhage growth, as they all defined specific factors using their own unique definitions and thus could not be compared [18], [23], [28]–[31]. Arguably, the way one defines a prognostic factor, will ultimately determine if one observes a significant association with deterioration. Further, although we investigated heterogeneity in all factors comparisons and felt it was reasonable to combine these studies, some factor comparisons still had very few studies and some studies dichotomized their factors at different levels of the prognostic factor spectrum which may have resulted in high variability and imprecise ORs. With regard to these factors, caution is warranted when drawing conclusions. It is clear that future studies should be diligent to define factors using commonly accepted definitions to allow for comparison and summary conclusions to be drawn.

Further, our study was limited in that we did not have individual patient data, or complete information on the time from symptom onset to hospital admission for in-depth analysis. Also, we could not assess publication bias using formal statistical methods. We stratified studies based on treatments reported and quality factors [12], however we acknowledge that it is still possible that specific treatments may have influenced END in a systematic way. It should be noted that the medical treatments patients received in this review were similar to what is recommended in the guidelines for ICH care [26], [32], and since there is no proven effective treatment for ICH, one would expect that any influence a specific medical treatment had on END in this study, would have been small.

## Conclusion

Many authors have emphasized the need to summarize the literature on neurologic deterioration before new therapies can be tested appropriately [33], [34]. We believe this study provides a thorough summary of the evidence to date on ICH prognosis. Overall, this review highlights that the extent of bleeding at hospital admission may be the most important factor in determining early clinical outcomes, and highlights the lack of understanding we still have on many aspects of ICH prognosis.

## Supporting Information

Checklist S1 **Preferred Reporting Items for Systematic Reviews and Meta-Analyses (PRISMA) Checklist.**
(DOC)

Data Supplement S1 **Systematic review electronic search strategy.**
(DOC)

## References

[1] Qureshi AI, Tuhrim S, Broderick JP, Batjer HH, Hondo H, et al (2001) Spontaneous intracerebral hemorrhage. N Engl J Med 344: 1450–1460. 11346811 10.1056/NEJM200105103441907

[2] Fogelholm R, Murros K, Rissanen A, Avikainen S (2005) Long term survival after primary intracerebral haemorrhage: a retrospective population based study. J Neurol Neurosurg Psychiatry 76: 1534–1538. 16227546 10.1136/jnnp.2004.055145 PMC1739413

[3] Mayer SA, Sacco RL, Shi T, Mohr JP (1994) Neurologic deterioration in noncomatose patients with supratentorial intracerebral hemorrhage. Neurology 44: 1379–1384. 8058133 10.1212/wnl.44.8.1379

[4] Qureshi AI, Safdar K, Weil J, Barch C, Bliwise DL, et al (1995) Predictors of early deterioration and mortality in black Americans with spontaneous intracerebral hemorrhage. Stroke 26: 1764–1767. 7570722 10.1161/01.str.26.10.1764

[5] Mayer SA, Brun NC, Begtrup K, Broderick J, Davis S, et al (2008) Efficacy and safety of recombinant activated factor VII for acute intracerebral hemorrhage. N Engl J Med 358: 2127–2137. 18480205 10.1056/NEJMoa0707534

[6] Anderson CS, Heeley E, Huang Y, Wang J, Stapf C, et al (2013) Rapid blood-pressure lowering in patients with acute intracerebral hemorrhage. N Engl J Med 368: 2355–2365. 23713578 10.1056/NEJMoa1214609

[7] Tuhrim S (2008) Intracerebral hemorrhage—improving outcome by reducing volume? N Engl J Med 358: 2174–2176. 18480212 10.1056/NEJMe0801856

[8] Moher D, Hopewell S, Schulz KF, Montori V, Gotzsche PC, et al (2010) CONSORT 2010 Explanation and Elaboration: Updated guidelines for reporting parallel group randomised trials. J Clin Epidemiol 63: e1–37. 20346624 10.1016/j.jclinepi.2010.03.004

[9] Ariesen MJ, Claus SP, Rinkel GJ, Algra A (2003) Risk factors for intracerebral hemorrhage in the general population: a systematic review. Stroke 34: 2060–2065. 12843354 10.1161/01.STR.0000080678.09344.8D

[10] Nuance (2012) PDF Converter Professional.

[11] Stern JAC (2009) Meta-Analysis in Stata: An Updated Collection from the Stata JournalCollege Station, TexasStata Press257.

[12] Egger M, Smith GD, Altman DG (2001) Systematic Reviews in Health Care. Meta-analysis in contextTavistock Square, London, United KingdomBMJ Publishing Group487.

[13] Borenstein M, Hedges LV, Higgins JPT, Rothstein HR (2009) Introduction to Meta-Analysis. Hoboken, NJ: John Wiley and Sons, Ltd. 421 p.

[14] Last JM (2001) A Dictionary of EpidemiologyNew YorkOxford University Press196.

[15] Zhang J, Yu KF (1998) What's the relative risk? A method of correcting the odds ratio in cohort studies of common outcomes. JAMA 280: 1690–1691. 9832001 10.1001/jama.280.19.1690

[16] (2011) Stata Statistical Software: Release 12. College Station: StataCorp.

[17] Tetri S, Juvela S, Saloheimo P, Pyhtinen J, Hillbom M (2009) Hypertension and diabetes as predictors of early death after spontaneous intracerebral hemorrhage. J Neurosurg 110: 411–417. 19249937 10.3171/2008.8.JNS08445

[18] Tetri S, Mantymaki L, Juvela S, Saloheimo P, Pyhtinen J, et al (2008) Impact of ischemic heart disease and atrial fibrillation on survival after spontaneous intracerebral hemorrhage. J Neurosurg 108: 1172–1177. 18518724 10.3171/JNS/2008/108/6/1172

[19] Sterne JA, Gavaghan D, Egger M (2000) Publication and related bias in meta-analysis: power of statistical tests and prevalence in the literature. J Clin Epidemiol 53: 1119–1129. 11106885 10.1016/s0895-4356(00)00242-0

[20] Tan X, He J, Li L, Yang G, Liu H, et al (2014) Early hyperglycaemia and the early-term death in patients with spontaneous intracerebral haemorrhage: a meta-analysis. Intern Med J 44: 254–260. 24372661 10.1111/imj.12352

[21] Fogelholm R, Murros K, Rissanen A, Avikainen S (2005) Admission blood glucose and short term survival in primary intracerebral haemorrhage: a population based study. J Neurol Neurosurg Psychiatry 76: 349–353. 15716524 10.1136/jnnp.2003.034819 PMC1739544

[22] Castellanos M, Leira R, Tejada J, Gil-Peralta A, Davalos A, et al (2005) Predictors of good outcome in medium to large spontaneous supratentorial intracerebral haemorrhages. J Neurol Neurosurg Psychiatry 76: 691–695. 15834028 10.1136/jnnp.2004.044347 PMC1739633

[23] Delgado P, Alvarez-Sabin J, Abilleira S, Santamarina E, Purroy F, et al (2006) Plasma d-dimer predicts poor outcome after acute intracerebral hemorrhage. Neurology 67: 94–98. 16832084 10.1212/01.wnl.0000223349.97278.e0

[24] Hanley DF (2009) Intraventricular hemorrhage: severity factor and treatment target in spontaneous intracerebral hemorrhage. Stroke 40: 1533–1538. 19246695 10.1161/STROKEAHA.108.535419 PMC2744212

[25] Brott T, Broderick J, Kothari R, Barsan W, Tomsick T, et al (1997) Early hemorrhage growth in patients with intracerebral hemorrhage. Stroke 28: 1–5. 8996478 10.1161/01.str.28.1.1

[26] Morgenstern LB, Hemphill JC 3rd, Anderson C, Becker K, Broderick JP, et al (2010) Guidelines for the management of spontaneous intracerebral hemorrhage: a guideline for healthcare professionals from the American Heart Association/American Stroke Association. Stroke 41: 2108–2129. 20651276 10.1161/STR.0b013e3181ec611b PMC4462131

[27] Bruce SS, Appelboom G, Piazza M, Hwang BY, Kellner C, et al.. (2011) A Comparative Evaluation of Existing Grading Scales in Intracerebral Hemorrhage. Neurocrit Care. 10.1007/s12028-011-9518-7 21394545

[28] Hanger HC, Fletcher VJ, Wilkinson TJ, Brown AJ, Frampton CM, et al (2008) Effect of aspirin and warfarin on early survival after intracerebral haemorrhage. J Neurol 255: 347–352. 18297333 10.1007/s00415-008-0650-z

[29] Rodriguez-Luna D, Rubiera M, Ribo M, Coscojuela P, Pineiro S, et al (2011) Ultraearly hematoma growth predicts poor outcome after acute intracerebral hemorrhage. Neurology 77: 25. 10.1212/WNL.0b013e3182343387 21998314

[30] Sorimachi T, Fujii Y (2010) Early neurological change in patients with spontaneous supratentorial intracerebral hemorrhage. J Clin Neurosci 17: November. 10.1016/j.jocn.2010.02.024 20692165

[31] Zhang X, Lu XM, Huang LF, Ye H (2012) Copeptin is associated with one-year mortality and functional outcome in patients with acute spontaneous basal ganglia hemorrhage. Peptides 33: February. 10.1016/j.peptides.2012.01.011 22286033

[32] Broderick JP, Adams HP Jr, Barsan W, Feinberg W, Feldmann E, et al (1999) Guidelines for the management of spontaneous intracerebral hemorrhage: A statement for healthcare professionals from a special writing group of the Stroke Council, American Heart Association. Stroke 30: 905–915. 10187901 10.1161/01.str.30.4.905

[33] Hobart J (2007) Measuring outcomes in clinical trials of stroke: time for state-of-the-art clinical trials to reject state-of-the-ark rating scales. J Neurol 254: 1119. 17762947 10.1007/s00415-007-0535-6

[34] Creutzfeldt CJ, Tirschwell DL (2008) Intracerebral hemorrhage: balancing death versus disability. Crit Care Med 36: 2214–2215. 18594240 10.1097/CCM.0b013e31817e2605

[35] Franke CL, van Swieten JC, Algra A, van Gijn J (1992) Prognostic factors in patients with intracerebral haematoma. J Neurol Neurosurg Psychiatry 55: 653–657. 1527534 10.1136/jnnp.55.8.653 PMC489199

[36] Lampl Y, Gilad R, Eshel Y, Sarova-Pinhas I (1995) Neurological and functional outcome in patients with supratentorial hemorrhages. A prospective study. Stroke 26: 2249–2253. 7491645 10.1161/01.str.26.12.2249

[37] Abilleira S, Montaner J, Molina CA, Monasterio J, Castillo J, et al (2003) Matrix metalloproteinase-9 concentration after spontaneous intracerebral hemorrhage. J Neurosurg 99: 65–70. 12854746 10.3171/jns.2003.99.1.0065

[38] Sykora M, Diedler J, Turcani P, Rupp A, Steiner T (2009) Subacute perihematomal edema in intracerebral hemorrhage is associated with impaired blood pressure regulation. J Neurol Sci 284: 108–112. 19428030 10.1016/j.jns.2009.04.028

[39] Ohwaki K, Yano E, Nagashima H, Hirata M, Nakagomi T, et al.. (2010) Blood pressure management in acute intracerebral haemorrhage: Low blood pressure and early neurological deterioration. Br J Neurosurg 24: August. 10.3109/02688691003746282 20632876

[40] Johkura K, Nakae Y, Yamamoto R, Mitomi M, Kudo Y (2011) Wrong-way deviation: Contralateral conjugate eye deviation in acute supratentorial stroke. J Neurol Sci 308: 15. 10.1016/j.jns.2011.06.010 21705030

