# Supplementary material for: Factors Associated with Early Deterioration after Spontaneous Intracerebral Hemorrhage: A Systematic Review and Meta-Analysis
Source: PLoS One. 2014 May 8;9(5):e96743. doi: 10.1371/journal.pone.0096743 (PMC4014549; doi:10.1371/journal.pone.0096743)
Supplement: Data Supplement S1 — Systematic review electronic search strategy. (DOC) [file pone.0096743.s002.doc]

**Data Supplement s1**

Systematic Review Electronic Search Strategy

Procedures:

1) The titles and abstracts of citations identified in EMBASE, PubMed, the Cochrane Library, and the Global Health Library were screened and retained for further review if they 1) examined adult ICH patients, or 2) they measured or reported death or deterioration at any time during follow-up, or 3) the reviewer was uncertain of any of the above. A full-text copy of studies which met any of the three aforementioned criteria during the abstract screening process was obtained from our university holdings or Docline; a multinational interlibrary document delivery service.

2) Papers were translated to English and converted to searchable PDFs, then searched for keywords and phrases using PDF Converter Pro 8.0 software.

3) Papers were retained for further review by an expert if they contained at least one of these words or phrases.

**Step 1: Electronic Database Search**

EMBASE:

The following keywords and phrases were searched in EMBASE:

1. bleeding stroke.tw.

2. brain haematoma.tw.

3. brain haemorrhage.tw.

4. brain hematoma.tw.

5. brain hemorrhage.tw.

6. cerebral haematoma.tw.

7. cerebral haemorrhage.tw.

8. cerebral hematoma.tw.

9. cerebral hemorrhage.tw.

10. haemorrhagic stroke.tw.

11. hypertensive cerebral haematoma.tw.

12. hypertensive cerebral haemorrhage.tw.

13. hypertensive cerebral hematoma.tw.

14. hypertensive cerebral hemorrhage.tw.

15. hypertensive ICH.tw.

16. hypertensive infratentorial haemorrhage.tw.

17. hypertensive infratentorial hemorrhage.tw.

18. hypertensive intracerebral haemorrhage.tw.

19. hypertensive intracerebral hemorrhage.tw.

20. hypertensive parenchymal haematoma.tw.

21. hypertensive parenchymal haemorrhage.tw.

22. hypertensive parenchymal hematoma.tw.

23. hypertensive parenchymal hemorrhage.tw.

24. ICH.tw.

25. intracerebral haemorrhage.tw.

26. intracerebral hemorrhage.tw.

27. intracranial haemorrhage.tw.

28. intracranial hematoma.tw.

29. intracranial hemorrhage.tw.

30. intracranial haemorrhage.tw.

31. parenchymal haemorrhage.tw.

32. parenchymal hemorrhage.tw.

33. pICH.tw.

34. PICH.tw.

35. primary cerebral haematoma.tw.

36. primary cerebral haemorrhage.tw.

37. primary cerebral hematoma.tw.

38. primary cerebral hemorrhage.tw.

39. primary infratentorial haemorrhage.tw.

40. primary infratentorial hemorrhage.tw.

41. primary intracerebral haemorrhage.tw.

42. primary intracerebral hemorrhage.tw.

43. primary intracranial haematoma.tw.

44. primary intracranial haemorrhage.tw.

45. primary intracranial hematoma.tw.

46. primary intracranial hemorrhage.tw.

47. primary supratentorial haemorrhage.tw.

48. primary supratentorial hemorrhage.tw.

49. spontaneous cerebral haematoma.tw.

50. spontaneous cerebral haemorrhage.tw.

51. spontaneous cerebral hematoma.tw.

52. spontaneous cerebral hemorrhage.tw.

53. spontaneous hemispheric ICH.tw.

54. spontaneous infratentorial haemorrhage.tw.

55. spontaneous infratentorial hemorrhage.tw.

56. spontaneous intracerebral haemorrhage.tw.

57. spontaneous intracerebral hemorrhage.tw.

58. spontaneous parenchymal haematoma.tw.

59. spontaneous parenchymal haemorrhage.tw.

60. spontaneous parenchymal hematoma.tw.

61. spontaneous parenchymal hemorrhage.tw.

62. spontaneous supratentorial haemorrhage.tw.

63. spontaneous supratentorial hemorrhage.tw.

64. 1 OR 2 OR 3 OR 4 OR 5 OR 6 OR 7 OR 8 OR 9 OR 10 OR 11 OR 12 OR 13 OR 14 OR 15 OR 16 OR 17 OR 18 OR 19 OR 20 OR 21 OR 22 OR 23 OR 24 OR 25 OR 26 OR 27 OR 28 OR 29 OR 30 OR 31 OR 32 OR 33 OR 34 OR 35 OR 36 OR 37 OR 38 OR 39 OR 40 OR 41 OR 42 OR 43 OR 44 OR 45 OR 46 OR 47 OR 48 OR 49 OR 50 OR 51 OR 52 OR 53 OR 54 OR 55 OR 56 OR 57 OR 58 OR 59 OR 60 OR 61 OR 62 OR 63

65. Canadian Neurological Scale.tw.

66. Canadian Stroke Scale.tw.

67. CNS.tw.

68. CSS.tw.

69. ESS.tw.

70. European Stroke Scale.tw.

71. Hemispheric Stroke Scale.tw.

72. HSS.tw.

73. Hunt & Hess Scale.tw.

74. ICH Scale.tw.

75. ICH Score.tw.

76. Mathew Stroke Scale.tw.

77. MCA Scale.tw.

78. MCA Score.tw.

79. Middle Cerebral Artery Scale.tw.

80. mNIHSS.tw.

81. MNIHSS.tw.

82. modified national institutes of health stroke scale.tw.

83. national institutes of health stroke scale.tw.

84. NIH Stroke Scale.tw.

85. NIHSS.tw.

86. Orgogozo Stroke Scale.tw.

87. Oxfordshire Community Stroke Project Classification.tw.

88. Scandinavian Stroke Scale.tw.

89. severity of ICH.tw.

90. SSS.tw.

91. stroke severity.tw.

92. early deterioration.tw.

93. early neurologic deterioration.tw.

94. consciousness.tw.

95. GCS.tw.

96. glasgow coma scale.tw.

97. glasgow coma score.tw.

98. level of consciousness.tw.

99. deteriorated.tw.

100. deterioration.tw.

101. all-cause mortality.tw.

102. case-fatality.tw.

103. case-mortality.tw.

104. dead.tw.

105. death.tw.

106. death rate.tw.

107. fatality.tw.

108. in-hospital death.tw.

109. in-hospital fatality.tw.

110. in-hospital mortality.tw.

111. mortality.tw.

112. survival.tw.

113. survival rate.tw.

114. 65 OR 66 OR 67 OR 68 OR 69 OR 70 OR 71 OR 72 OR 73 OR 74 OR 75 OR 76 OR 77 OR 78 OR 79 OR 80 OR 81 OR 82 OR 83 OR 84 OR 85 OR 86 OR 87 OR 88 OR 89 OR 90 OR 91 OR 92 OR 93 OR 94 OR 95 OR 96 OR 97 OR 98 OR 99 OR 100 OR 101 OR 102 OR 103 OR 104 OR 105 OR 106 OR 107 OR 108 OR 109 OR 110 OR 111 OR 112 OR 113

115. cox hazard.tw.

116. cox proportional hazard.tw.

117. empirical.tw.

118. empirical research.tw.

119. epidemiology.tw.

120. frequency.tw.

121. hazard.tw.

122. hazard estimate.tw.

123. hazard ratio.tw.

124. Kaplan-Meiers estimate.tw.

125. linear models.tw.

126. logistic models.tw.

127. multivariable.tw.

128. multivariate.tw.

129. multivariable analysis.tw.

130. multivariate analysis.tw.

131. occurrence.tw.

132. odds.tw.

133. odds estimate.tw.

134. odds ratio.tw.

135. outcome.tw.

136. outcomes.tw.

137. prediction.tw.

138. predictor.tw.

139. predictors.tw.

140. prognosis.tw.

141. prognosis.tw.

142. prognostic.tw.

143. regression analysis.tw.

144. relative risk.tw.

145. risk.tw.

146. risk assessment.tw.

147. risk estimate.tw.

148. risk ratio.tw.

149. risks.tw.

150. sensitivity.tw.

151. specificity.tw.

152. ROC Curve.tw.

153. treatment failure.tw.

154. treatment outcome.tw.

155. incidence.tw.

156. 115 OR 116 OR 117 OR 118 OR 119 OR 120 OR 121 OR 122 OR 123 OR 124 OR 125 OR 126 OR 127 OR 128 OR 129 OR 130 OR 131 OR 132 OR 133 OR 134 OR 135 OR 136 OR 137 OR 138 OR 139 OR 140 OR 141 OR 142 OR 143 OR 144 OR 145 OR 146 OR 147 OR 148 OR 149 OR 150 OR 151 OR 152 OR 153 OR 154 OR 155

157. 64 AND 114 AND 156

158. limit 157 to animals

159. 157 NOT 158

160. limit 159 to animal studies

161. 159 NOT 160

162. limit 161 to (book OR book series OR editorial OR letter OR note OR proceeding OR "review" OR short survey)

163. 161 NOT 162

164. limit 163 to (embryo OR infant OR child OR preschool child < 1 to 6 years > OR school child < 7 to 12 years > OR adolescent < 13 to 17 years >)

165. 163 NOT 164

Notes:

i) “.tw” denotes the term was searched as a “text word”.

ii) The number preceding the terms denotes when the term was searched; such that “1” was first, “2” was second etc.

iii) “OR”, “AND” and “NOT” are Boolean Operators.

PubMed:

The following keywords and phrases were searched in PubMed:

((((adult) OR ("Adult"[Mesh]) OR (adults) OR ("Aged"[Mesh]) OR ("Aged, 80 and over"[Mesh]) OR (elderly) OR ("frail elderly") OR ("Frail Elderly"[Mesh]) OR ("Middle Aged"[Mesh])) AND ((Canadian Neurological Scale) OR (Canadian Stroke Scale) OR (CNS) OR (CSS) OR (ESS) OR (European Stroke Scale) OR (Hemispheric Stroke Scale) OR (HSS) OR (Hunt AND Hess Scale) OR (ICH Scale) OR (ICH Score) OR (Mathew Stroke Scale) OR (MCA Scale) OR (MCA Score) OR (Middle Cerebral Artery Scale) OR (mNIHSS) OR (MNIHSS) OR (modified national institutes of health stroke scale) OR (national institutes of health stroke scale) OR (NIH Stroke Scale) OR (NIHSS) OR (Orgogozo Stroke Scale) OR (Oxfordshire Community Stroke Project Classification AND (Bamford)) OR (Scandinavian Stroke Scale) OR (severity of ICH) OR (SSS) OR (stroke severity) OR (early deterioration) OR (early neurologic deterioration) OR ("Consciousness"[Mesh]) OR (GCS) OR (glasgow coma scale) OR ("Glasgow Coma Scale"[Mesh]) OR (glasgow coma score) OR (level of consciousness)) AND ((all-cause mortality) OR (case-fatality) OR (case-mortality) OR (dead) OR (death) OR ("Death"[Mesh]) OR (death rate) OR (fatality) OR (in-hospital death) OR (in-hospital fatality) OR (in-hospital mortality) OR (mortality) OR ("Mortality"[Mesh]) OR ("Survival"[Mesh]) OR (survival rate) OR ("Survival Rate"[Mesh])) AND ((cox hazard) OR (cox proportional hazard) OR (empirical) OR ("Empirical Research"[Mesh]) OR (epidemiology) OR ("Epidemiology"[Mesh]) OR (frequency) OR (hazard) OR (hazard estimate) OR (hazard ratio) OR (Kaplan-Meiers estimate) OR ("Kaplan-Meiers Estimate"[Mesh]) OR (linear models) OR (logistic models) OR (multivariable) OR (multivariate) OR (multivariable analysis) OR (multivariate analysis) OR ("Multivariate Analysis"[Mesh]) OR (occurrence) OR (odds) OR (odds estimate) OR (odds ratio) OR (outcome) OR (outcomes) OR (prediction) OR (predictor) OR (predictors) OR (prognosis) OR ("Prognosis"[Mesh]) OR (prognostic) OR ("Regression Analysis"[Mesh]) OR (relative risk) OR (risk) OR (risk assessment) OR ("Risk Assessment"[Mesh]) OR (risk estimate) OR (risk ratio) OR (risks) OR (sensitivity) OR ("Sensitivity and Specificity"[Mesh]) OR (specificity) OR (time to event) OR ("ROC Curve"[Mesh]) OR (ROC Curve) OR (treatment failure) OR ("Treatment Failure"[Mesh]) OR (treatment outcome) OR ("Treatment Outcome"[Mesh]) OR (incidence) OR ("Incidence"[Mesh]) OR (prevalence) OR ("Prevalence"[Mesh])) AND ((bleeding stroke) OR (brain haematoma) OR (brain hematoma) OR (brain haemorrhage) OR (brain hemorrhage) OR (cerebral haematoma) OR (cerebral haemorrhage) OR (cerebral hematoma) OR (cerebral hemorrhage) OR ("Cerebral Hemorrhage"[Mesh]) OR (haemorrhagic stroke) OR (hemorrhagic stroke) OR (hypertensive cerebral haematoma) OR (hypertensive cerebral haemorrhage) OR (hypertensive cerebral hematoma) OR (hypertensive cerebral hemorrhage) OR (hypertensive ICH) OR (hypertensive infratentorial haemorrhage) OR (hypertensive infratentorial hemorrhage) OR (hypertensive intracerebral haemorrhage) OR (hypertensive intracerebral hemorrhage) OR (hypertensive parenchymal haematoma) OR (hypertensive parenchymal haemorrhage) OR (hypertensive parenchymal hematoma) OR (hypertensive parenchymal hemorrhage) OR (ICH) OR (intracerebral haemorrhage) OR (intracerebral hemorrhage) OR ("Intracranial Hemorrhages"[Mesh]) OR (intracranial haemorrhage) OR (intracranial hematoma) OR (intracranial hemorrhage) OR (parenchymal haemorrhage) OR (parenchymal hemorrhage) OR (pICH) OR (PICH) OR (primary cerebral haematoma) OR (primary cerebral haemorrhage) OR (primary cerebral hematoma) OR (primary cerebral hemorrhage) OR (primary infratentorial haemorrhage) OR (primary infratentorial hemorrhage) OR (primary intracerebral haemorrhage) OR (primary intracerebral hemorrhage) OR (primary intracranial haematoma) OR (primary intracranial haemorrhage) OR (primary intracranial hematoma) OR (primary intracranial hemorrhage) OR (primary supratentorial haemorrhage) OR (primary supratentorial hemorrhage) OR (spontaneous cerebral haematoma) OR (spontaneous cerebral haemorrhage) OR (spontaneous cerebral hematoma) OR (spontaneous cerebral hemorrhage) OR (spontaneous hemispheric ICH) OR (spontaneous infratentorial haemorrhage) OR (spontaneous infratentorial hemorrhage) OR (spontaneous intracerebral haemorrhage) OR (spontaneous intracerebral hemorrhage) OR (spontaneous parenchymal haematoma) OR (spontaneous parenchymal haemorrhage) OR (spontaneous parenchymal hematoma) OR (spontaneous parenchymal hemorrhage) OR (spontaneous supratentorial haemorrhage) OR (spontaneous supratentorial hemorrhage))) NOT (((adult) OR ("Adult"[Mesh]) OR (adults) OR ("Aged"[Mesh]) OR ("Aged, 80 and over"[Mesh]) OR (elderly) OR ("frail elderly") OR ("Frail Elderly"[Mesh]) OR ("Middle Aged"[Mesh])) AND ((Canadian Neurological Scale) OR (Canadian Stroke Scale) OR (CNS) OR (CSS) OR (ESS) OR (European Stroke Scale) OR (Hemispheric Stroke Scale) OR (HSS) OR (Hunt AND Hess Scale) OR (ICH Scale) OR (ICH Score) OR (Mathew Stroke Scale) OR (MCA Scale) OR (MCA Score) OR (Middle Cerebral Artery Scale) OR (mNIHSS) OR (MNIHSS) OR (modified national institutes of health stroke scale) OR (national institutes of health stroke scale) OR (NIH Stroke Scale) OR (NIHSS) OR (Orgogozo Stroke Scale) OR (Oxfordshire Community Stroke Project Classification AND (Bamford)) OR (Scandinavian Stroke Scale) OR (severity of ICH) OR (SSS) OR (stroke severity) OR (early deterioration) OR (early neurologic deterioration) OR ("Consciousness"[Mesh]) OR (GCS) OR (glasgow coma scale) OR ("Glasgow Coma Scale"[Mesh]) OR (glasgow coma score) OR (level of consciousness)) AND ((all-cause mortality) OR (case-fatality) OR (case-mortality) OR (dead) OR (death) OR ("Death"[Mesh]) OR (death rate) OR (fatality) OR (in-hospital death) OR (in-hospital fatality) OR (in-hospital mortality) OR (mortality) OR ("Mortality"[Mesh]) OR ("Survival"[Mesh]) OR (survival rate) OR ("Survival Rate"[Mesh])) AND ((cox hazard) OR (cox proportional hazard) OR (empirical) OR ("Empirical Research"[Mesh]) OR (epidemiology) OR ("Epidemiology"[Mesh]) OR (frequency) OR (hazard) OR (hazard estimate) OR (hazard ratio) OR (Kaplan-Meiers estimate) OR ("Kaplan-Meiers Estimate"[Mesh]) OR (linear models) OR (logistic models) OR (multivariable) OR (multivariate) OR (multivariable analysis) OR (multivariate analysis) OR ("Multivariate Analysis"[Mesh]) OR (occurrence) OR (odds) OR (odds estimate) OR (odds ratio) OR (outcome) OR (outcomes) OR (prediction) OR (predictor) OR (predictors) OR (prognosis) OR ("Prognosis"[Mesh]) OR (prognostic) OR ("Regression Analysis"[Mesh]) OR (relative risk) OR (risk) OR (risk assessment) OR ("Risk Assessment"[Mesh]) OR (risk estimate) OR (risk ratio) OR (risks) OR (sensitivity) OR ("Sensitivity and Specificity"[Mesh]) OR (specificity) OR (time to event) OR ("ROC Curve"[Mesh]) OR (ROC Curve) OR (treatment failure) OR ("Treatment Failure"[Mesh]) OR (treatment outcome) OR ("Treatment Outcome"[Mesh]) OR (incidence) OR ("Incidence"[Mesh]) OR (prevalence) OR ("Prevalence"[Mesh])) AND ((bleeding stroke) OR (brain haematoma) OR (brain hematoma) OR (brain haemorrhage) OR (brain hemorrhage) OR (cerebral haematoma) OR (cerebral haemorrhage) OR (cerebral hematoma) OR (cerebral hemorrhage) OR ("Cerebral Hemorrhage"[Mesh]) OR (haemorrhagic stroke) OR (hemorrhagic stroke) OR (hypertensive cerebral haematoma) OR (hypertensive cerebral haemorrhage) OR (hypertensive cerebral hematoma) OR (hypertensive cerebral hemorrhage) OR (hypertensive ICH) OR (hypertensive infratentorial haemorrhage) OR (hypertensive infratentorial hemorrhage) OR (hypertensive intracerebral haemorrhage) OR (hypertensive intracerebral hemorrhage) OR (hypertensive parenchymal haematoma) OR (hypertensive parenchymal haemorrhage) OR (hypertensive parenchymal hematoma) OR (hypertensive parenchymal hemorrhage) OR (ICH) OR (intracerebral haemorrhage) OR (intracerebral hemorrhage) OR ("Intracranial Hemorrhages"[Mesh]) OR (intracranial haemorrhage) OR (intracranial hematoma) OR (intracranial hemorrhage) OR (parenchymal haemorrhage) OR (parenchymal hemorrhage) OR (pICH) OR (PICH) OR (primary cerebral haematoma) OR (primary cerebral haemorrhage) OR (primary cerebral hematoma) OR (primary cerebral hemorrhage) OR (primary infratentorial haemorrhage) OR (primary infratentorial hemorrhage) OR (primary intracerebral haemorrhage) OR (primary intracerebral hemorrhage) OR (primary intracranial haematoma) OR (primary intracranial haemorrhage) OR (primary intracranial hematoma) OR (primary intracranial hemorrhage) OR (primary supratentorial haemorrhage) OR (primary supratentorial hemorrhage) OR (spontaneous cerebral haematoma) OR (spontaneous cerebral haemorrhage) OR (spontaneous cerebral hematoma) OR (spontaneous cerebral hemorrhage) OR (spontaneous hemispheric ICH) OR (spontaneous infratentorial haemorrhage) OR (spontaneous infratentorial hemorrhage) OR (spontaneous intracerebral haemorrhage) OR (spontaneous intracerebral hemorrhage) OR (spontaneous parenchymal haematoma) OR (spontaneous parenchymal haemorrhage) OR (spontaneous parenchymal hematoma) OR (spontaneous parenchymal hemorrhage) OR (spontaneous supratentorial haemorrhage) OR (spontaneous supratentorial hemorrhage)) AND ((Editorial[ptyp] OR Letter[ptyp] OR Review[ptyp] OR Comment[ptyp]))))

Notes:

i) “OR”, “AND” and “NOT” are Boolean Operators.

ii) “*phrase*”[Mesh] denotes that the term was searched as a Medical Subject Heading.

iii) *word*[ptyp] denotes Publication Type.

Limits Used:

Humans, All Adult: 19+ years, Adolescent: 13-18 years, Young Adult: 19-24 years, Adult: 19-44 years, Middle Aged: 45-64 years, Middle Aged + Aged: 45+ years, Aged: 65+ years, 80 and over: 80+ years

Cochrane Library:

The Cochrane Library is comprised of multiple databases. The Cochrane Central Register of Controlled Trials and The Cochrane Methodology Register were searched independently using the following keywords and phrases:

1. bleeding stroke.tw.

2. brain haematoma.tw.

3. brain haemorrhage.tw.

4. brain hematoma.tw.

5. brain hemorrhage.tw.

6. cerebral haematoma.tw.

7. cerebral haemorrhage.tw.

8. cerebral hematoma.tw.

9. cerebral hemorrhage.tw.

10. haemorrhagic stroke.tw.

11. hypertensive cerebral haematoma.tw.

12. hypertensive cerebral haemorrhage.tw.

13. hypertensive cerebral hematoma.tw.

14. hypertensive cerebral hemorrhage.tw.

15. hypertensive ICH.tw.

16. hypertensive infratentorial haemorrhage.tw.

17. hypertensive infratentorial hemorrhage.tw.

18. hypertensive intracerebral haemorrhage.tw.

19. hypertensive intracerebral hemorrhage.tw.

20. hypertensive parenchymal haematoma.tw.

21. hypertensive parenchymal haemorrhage.tw.

22. hypertensive parenchymal hematoma.tw.

23. hypertensive parenchymal hemorrhage.tw.

24. ICH.tw.

25. intracerebral haemorrhage.tw.

26. intracerebral hemorrhage.tw.

27. intracranial haemorrhage.tw.

28. intracranial hematoma.tw.

29. intracranial hemorrhage.tw.

30. intracranial haemorrhage.tw.

31. parenchymal haemorrhage.tw.

32. parenchymal hemorrhage.tw.

33. pICH.tw.

34. PICH.tw.

35. primary cerebral haematoma.tw.

36. primary cerebral haemorrhage.tw.

37. primary cerebral hematoma.tw.

38. primary cerebral hemorrhage.tw.

39. primary infratentorial haemorrhage.tw.

40. primary infratentorial hemorrhage.tw.

41. primary intracerebral haemorrhage.tw.

42. primary intracerebral hemorrhage.tw.

43. primary intracranial haematoma.tw.

44. primary intracranial haemorrhage.tw.

45. primary intracranial hematoma.tw.

46. primary intracranial hemorrhage.tw.

47. primary supratentorial haemorrhage.tw.

48. primary supratentorial hemorrhage.tw.

49. spontaneous cerebral haematoma.tw.

50. spontaneous cerebral haemorrhage.tw.

51. spontaneous cerebral hematoma.tw.

52. spontaneous cerebral hemorrhage.tw.

53. spontaneous hemispheric ICH.tw.

54. spontaneous infratentorial haemorrhage.tw.

55. spontaneous infratentorial hemorrhage.tw.

56. spontaneous intracerebral haemorrhage.tw.

57. spontaneous intracerebral hemorrhage.tw.

58. spontaneous parenchymal haematoma.tw.

59. spontaneous parenchymal haemorrhage.tw.

60. spontaneous parenchymal hematoma.tw.

61. spontaneous parenchymal hemorrhage.tw.

62. spontaneous supratentorial haemorrhage.tw.

63. spontaneous supratentorial hemorrhage.tw.

64. 1 OR 2 OR 3 OR 4 OR 5 OR 6 OR 7 OR 8 OR 9 OR 10 OR 11 OR 12 OR 13 OR 14 OR 15 OR 16 OR 17 OR 18 OR 19 OR 20 OR 21 OR 22 OR 23 OR 24 OR 25 OR 26 OR 27 OR 28 OR 29 OR 30 OR 31 OR 32 OR 33 OR 34 OR 35 OR 36 OR 37 OR 38 OR 39 OR 40 OR 41 OR 42 OR 43 OR 44 OR 45 OR 46 OR 47 OR 48 OR 49 OR 50 OR 51 OR 52 OR 53 OR 54 OR 55 OR 56 OR 57 OR 58 OR 59 OR 60 OR 61 OR 62 OR 63

65. Canadian Neurological Scale.tw.

66. Canadian Stroke Scale.tw.

67. CNS.tw.

68. CSS.tw.

69. ESS.tw.

70. European Stroke Scale.tw.

71. Hemispheric Stroke Scale.tw.

72. HSS.tw.

73. Hunt & Hess Scale.tw.

74. ICH Scale.tw.

75. ICH Score.tw.

76. Mathew Stroke Scale.tw.

77. MCA Scale.tw.

78. MCA Score.tw.

79. Middle Cerebral Artery Scale.tw.

80. mNIHSS.tw.

81. MNIHSS.tw.

82. modified national institutes of health stroke scale.tw.

83. national institutes of health stroke scale.tw.

84. NIH Stroke Scale.tw.

85. NIHSS.tw.

86. Orgogozo Stroke Scale.tw.

87. Oxfordshire Community Stroke Project Classification.tw.

88. Scandinavian Stroke Scale.tw.

89. severity of ICH.tw.

90. SSS.tw.

91. stroke severity.tw.

92. early deterioration.tw.

93. early neurologic deterioration.tw.

94. consciousness.tw.

95. GCS.tw.

96. glasgow coma scale.tw.

97. glasgow coma score.tw.

98. level of consciousness.tw.

99. deteriorated.tw.

100. deterioration.tw.

101. all-cause mortality.tw.

102. case-fatality.tw.

103. case-mortality.tw.

104. dead.tw.

105. death.tw.

106. death rate.tw.

107. fatality.tw.

108. in-hospital death.tw.

109. in-hospital fatality.tw.

110. in-hospital mortality.tw.

111. mortality.tw.

112. survival.tw.

113. survival rate.tw.

114. 65 OR 66 OR 67 OR 68 OR 69 OR 70 OR 71 OR 72 OR 73 OR 74 OR 75 OR 76 OR 77 OR 78 OR 79 OR 80 OR 81 OR 82 OR 83 OR 84 OR 85 OR 86 OR 87 OR 88 OR 89 OR 90 OR 91 OR 92 OR 93 OR 94 OR 95 OR 96 OR 97 OR 98 OR 99 OR 100 OR 101 OR 102 OR 103 OR 104 OR 105 OR 106 OR 107 OR 108 OR 109 OR 110 OR 111 OR 112 OR 113

115. cox hazard.tw.

116. cox proportional hazard.tw.

117. empirical.tw.

118. empirical research.tw.

119. epidemiology.tw.

120. frequency.tw.

121. hazard.tw.

122. hazard estimate.tw.

123. hazard ratio.tw.

124. Kaplan-Meiers estimate.tw.

125. linear models.tw.

126. logistic models.tw.

127. multivariable.tw.

128. multivariate.tw.

129. multivariable analysis.tw.

130. multivariate analysis.tw.

131. occurrence.tw.

132. odds.tw.

133. odds estimate.tw.

134. odds ratio.tw.

135. outcome.tw.

136. outcomes.tw.

137. prediction.tw.

138. predictor.tw.

139. predictors.tw.

140. prognosis.tw.

141. prognosis.tw.

142. prognostic.tw.

143. regression analysis.tw.

144. relative risk.tw.

145. risk.tw.

146. risk assessment.tw.

147. risk estimate.tw.

148. risk ratio.tw.

149. risks.tw.

150. sensitivity.tw.

151. specificity.tw.

152. ROC Curve.tw.

153. treatment failure.tw.

154. treatment outcome.tw.

155. incidence.tw.

156. 115 OR 116 OR 117 OR 118 OR 119 OR 120 OR 121 OR 122 OR 123 OR 124 OR 125 OR 126 OR 127 OR 128 OR 129 OR 130 OR 131 OR 132 OR 133 OR 134 OR 135 OR 136 OR 137 OR 138 OR 139 OR 140 OR 141 OR 142 OR 143 OR 144 OR 145 OR 146 OR 147 OR 148 OR 149 OR 150 OR 151 OR 152 OR 153 OR 154 OR 155

157. 64 AND 114 AND 156

158. limit 157 to animals

159. 157 NOT 158

160. limit 159 to animal studies

161. 159 NOT 160

162. limit 161 to (book OR book series OR editorial OR letter OR note OR proceeding OR "review" OR short survey)

163. 161 NOT 162

164. limit 163 to (embryo OR infant OR child OR preschool child < 1 to 6 years > OR school child < 7 to 12 years > OR adolescent < 13 to 17 years >)

165. 163 NOT 164

Notes:

i) “.tw” denotes the term was searched as a “text word”.

ii) The number preceding the terms denotes when the term was searched; such that “1” was first, “2” was second etc.

iii) “OR”, “AND” and “NOT” are Boolean Operators.

The Global Health Library:

The Global Health Library is comprised of multiple regional online libraries which were searched simultaneously using the following keywords and phrases:

((Canadian Neurological Scale) OR (Canadian Stroke Scale) OR CNS OR CSS OR ESS OR ESS OR (European Stroke Scale) OR (Hemispheric Stroke Scale) OR (HSS ) OR (Hunt & Hess Scale ) OR (HSS ) OR (ICH Scale) OR (ICH Score) OR (Mathew Stroke Scale ) OR (MCA Scale) OR (MCA Score) OR (Middle Cerebral Artery Scale) OR mNIHSS OR MNIHSS OR (modified national institutes of health stroke scale) OR (national institutes of health stroke scale) OR (NIH Stroke Scale) OR NIHSS OR (Orgogozo Stroke Scale) OR (Oxfordshire Community Stroke Project Classification Bamford) OR (Scandinavian Stroke Scale) OR (severity of ICH ) OR SSS OR (stroke severity) OR (early deterioration) OR (early neurologic deterioration) OR consciousness OR GCS OR (glasgow coma scale) OR (glasgow coma scale) OR (glasgow coma score) OR (level of consciousness) OR (all-cause mortality) OR case-fatality OR case-mortality OR dead OR death OR death OR (death rate) OR fatality OR (in-hospital death) OR (in-hospital fatality) OR (in-hospital mortality) OR (in-hospital mortality) OR mortality OR mortality OR survival OR (survival rate) OR (survival rate) OR (survival rate)) AND ((bleeding stroke) OR (brain haematoma) OR (brain haemorrhage) OR (brain haemorrhage) OR (brain hematoma) OR (brain hemorrhage) OR (cerebral haematoma) OR (cerebral haemorrhage) OR (hypertensive cerebral hematoma) OR (hypertensive cerebral hemorrhage) OR (hypertensive ICH) OR (hypertensive infratentorial haemorrhage) OR (hypertensive infratentorial hemorrhage) OR (hypertensive intracerebral haemorrhage) OR (hypertensive intracerebral hemorrhage) OR (hypertensive parenchymal haematoma) OR (hypertensive parenchymal haemorrhage) OR (hypertensive parenchymal hematoma) OR (hypertensive parenchymal hemorrhage) OR ICH OR (intracerebral hemorrhage) OR (intracranial hemorrhages) OR (intracranial haemorrhage) OR (intracranial hemorrhage) OR (parenchymal haemorrhage) OR (parenchymal hemorrhage) OR pICH OR PICH OR (primary cerebral haematoma) OR (primary cerebral haemorrhage) OR (primary cerebral haemorrhage) OR (primary cerebral hematoma) OR (primary cerebral hemorrhage) OR (primary infratentorial haemorrhage) OR (primary infratentorial hemorrhage) OR (primary intracerebral haemorrhage) OR (primary intracerebral hemorrhage) OR (primary intracranial haematoma) OR (primary intracranial haemorrhage) OR (primary intracranial hematoma) OR (primary intracranial hemorrhage) OR (primary supratentorial haemorrhage) OR (primary supratentorial hemorrhage) OR (spontaneous cerebral haematoma) OR (spontaneous cerebral haemorrhage) OR (spontaneous cerebral hematoma) OR (spontaneous cerebral hemorrhage) OR (spontaneous hemispheric ICH ) OR (spontaneous infratentorial haemorrhage) OR (spontaneous infratentorial hemorrhage) OR (spontaneous intracerebral haemorrhage) OR (spontaneous intracerebral hemorrhage) OR (spontaneous parenchymal haematoma) OR (spontaneous parenchymal haemorrhage) OR (spontaneous parenchymal hematoma) OR (spontaneous parenchymal hemorrhage) OR (spontaneous parenchymal haemorrhage) OR (spontaneous parenchymal hemorrhage) OR (spontaneous supratentorial haemorrhage) OR (spontaneous supratentorial hemorrhage) OR (spontaneous supratentorial hemorrhage) OR (spontaneous supratentorial haemorrhage))

Notes:

i) The Global Health Library could not handle complex searches so only keywords for ICH and END were searched. This resulted in a less specific search compared to the ones used for PubMed, EMBASE, and the Cochrane Library.

Limits Used:

“Articles”

**Step 2: Full-Text Computer Screening**

The following are the synonyms / keywords / phrases for early neurologic deterioration and/or death within 1 week after ICH which were searched within full-text papers using PDF Converter Pro 8.0 software. Only one term needed to be found to retain the paper for further review.

Notes:

i) Phases/words in brackets were searched in the order they appeared in the brackets.

ii) “|” denotes Boolean Operator “OR”.

iii) Search was not case-sensitive.

iv) PDF Converter Pro 8.0 could not handle all of the phases at once so they were searched in parts.

v) Duplicate citations were removed.

vi) The term “unconscious” was deliberately not searched as it was felt it referred to baseline ICH severity rather than early neurologic deterioration specifically.

(acute decline)|(acute improvement)|(acute worsening)|(acutely improved)|(acutely improving)|(acutely worsened)|(change in CNS)|(change in ESS)|(change in Glasgow Coma Scale)|(change in Glasgow Coma Score)|(change in HSS)|(change in ICH Scale)|(change in ICH Score)|(change in Intracerebral Haemorrhage Scale)|(change in Intracerebral Haemorrhage Score)|(change in Intracerebral Hemorrhage Scale)|(change in Intracerebral Hemorrhage Score)|(change in Mathew Scale)|(change in Mathew Score)|(change in MCA Scale)|(change in MCA Score)|(change in mNIHSS)|(change in NIHSS)|(change in ONS)|(change in severity)|(change in SSS)|(change in TSS)|(clinical change)|(clinical deterioration)|(clinical improvement)|(CNS change)|(decline in Canadian Neurological Scale)|(decline in Canadian Neurological Score)|(decline in European Stroke Scale)|(decline in European Stroke Score)|(decline in Hemispheric Stroke Scale)|(decline in Mathew Scale)|(decline in Mathew Score)|(decline in Middle Cerebral Artery Scale)|(decline in Modified National Institutes of Health Stroke Scale)|(decline in Modified National Institutes of Health Stroke Score)|(decline in National Institutes of Health Stroke Scale)|(decline in National Institutes of Health Stroke Score)|(decline in neurologic function)|(decline in neurological function)|(decline in Orgogozo Neurological Scale)|(decline in Scandinavian Stroke Scale)|(decline in Scandinavian Stroke Score)|(decline in Toronto Stroke Scale)|(decrease in neurologic function)|(deteriorated clinically)|(deteriorated prior to discharge)|(deteriorated quickly)|(deteriorated rapidly)|(deteriorating neurologically)|(deterioration prior to discharge)|(drop in neurologic function)|(drop in neurologic function)|(drop in neurological function)|(early deterioration)|(early improvement)|(early neurologic decline)|(early neurologic deterioration)|(early neurologic improvement)|(early neurological deterioration)|(early neurological improvement)|(ESS change)|(GCS change)|(hospital deterioration)|(hospital improvement)|(HSS change)|(ICH Scale change)|(ICH Score change)|(improved clinically)|(improved prior to discharge)|(improved quickly)|(improved rapidly)|(improvement in Canadian Neurological Scale)|(improvement in Canadian Neurological Score)|(improvement in European Stroke Scale)|(improvement in European Stroke Score)|(improvement in Hemispheric Stroke Scale)|(improvement in Hemispheric Stroke Score)|(improvement in Mathew Scale)|(improvement in Mathew Score)|(improvement in Middle Cerebral Artery Scale)|(improvement in Middle Cerebral Artery Score)|(improvement in Modified National Institutes of Health Stroke Scale)|(improvement in Modified National Institutes of Health Stroke Score)|(improvement in Modified NIH Stroke Scale)|(improvement in Modified NIH Stroke Score)|(improvement in National Institutes of Health Stroke Scale)|(improvement in National Institutes of Health Stroke Score)|(improvement in neurologic function)|(improvement in NIH Stroke Scale)|(improvement in NIH Stroke Score)|(improvement in Orgogozo Neurological Scale)|(improvement in Orgogozo Neurological Score)|(improvement in Scandinavian Stroke Scale)|(improvement in Scandinavian Stroke Score)|(improvement in Toronto Stroke Scale)|(improvement in Toronto Stroke Score)|(improvement prior to discharge)|(improving Glasgow Coma Scale)|(improving Glasgow Coma Score)|(improving neurologically)|(improving of Canadian Neurological Scale)|(improving of Canadian Neurological Score)|(improving of CNS)|(improving of ESS)|(improving of European Stroke Scale)|(improving of European Stroke Score)|(improving of Hemispheric Stroke Scale)|(improving of Hemispheric Stroke Score)|(improving of HSS)|(improving of ICH Scale)|(improving of ICH Score)|(improving of Intracerebral Haemorrhage Scale)|(improving of Intracerebral Haemorrhage Score)|(improving of Intracerebral Hemorrhage Scale)|(improving of Intracerebral Hemorrhage Score)|(improving of Mathew Scale)|(improving of Mathew Score)|(improving of MCA Scale)|(improving of MCA Score)|(improving of Middle Cerebral Artery Scale)|(improving of Middle Cerebral Artery Score)|(improving of mNIHSS)|(improving of Modified National Institutes of Health Stroke Scale)|(improving of Modified National Institutes of Health Stroke Score)|(improving of Modified NIH Stroke Scale)|(improving of Modified NIH Stroke Score)|(improving of National Institutes of Health Stroke Scale)|(improving of National Institutes of Health Stroke Score)|(improving of NIH Stroke Scale)|(improving of NIH Stroke Score)|(improving of NIHSS)|(improving of ONS)|(improving of Orgogozo Neurological Scale)|(improving of Orgogozo Neurological Score)|(improving of Scandinavian Stroke Scale)|(improving of Scandinavian Stroke Score)|(improving of SSS)|(improving of Toronto Stroke Scale)|(improving of Toronto Stroke Score)|(improving of TSS)|(increase in neurologic function)|(Intracerebral Haemorrhage Scale change)|(Intracerebral Haemorrhage Score change)|(Intracerebral Hemorrhage Scale change)|(Intracerebral Hemorrhage Score change)|(MCA Scale change)|(MCA Score change)|(mNIHSS change)|(neurologic decline)|(neurologic improvement)|(neurological decline)|(neurological improvement)|(neurologically deteriorated)|(neurologically improved)|(neurologically worsened)|(NIHSS change)|(ONS change)|(pre-hospital deterioration)|(pre-hospital improvement)|(quickly deteriorated)|(quickly improved)|(quickly worsened)|(rapid deterioration)|(rapid improvement)|(rapidly deteriorated)|(rapidly improved)|(rapidly worsened)|(rise in neurologic function)|(rise in neurological function)|(short term outcome)|(short-term outcome)|(SSS change)|(TSS change)|(worsened clinically)|(worsened prior to discharge)|(worsened quickly)|(worsened rapidly)|(worsening GCS)|(worsening Glasgow Coma Scale)|(worsening Glasgow Coma Score)|(worsening of Canadian Neurological Scale)|(worsening of Canadian Neurological Score)|(worsening of CNS)|(worsening of ESS)|(worsening of European Stroke Scale)|(worsening of European Stroke Score)|(worsening of Hemispheric Stroke Scale)|(worsening of Hemispheric Stroke Score)|(worsening of HSS)|(worsening of ICH Scale)|(worsening of ICH Score)|(worsening of Intracerebral Haemorrhage Scale)|(worsening of Intracerebral Haemorrhage Score)|(worsening of Intracerebral Hemorrhage Scale)|(worsening of Intracerebral Hemorrhage Score)|(worsening of Mathew Scale)|(worsening of Mathew Score)|(worsening of MCA Scale)|(worsening of MCA Score)|(worsening of Middle Cerebral Artery Scale)|(worsening of Middle Cerebral Artery Score)|(worsening of mNIHSS)|(worsening of Modified National Institutes of Health Stroke Scale)|(worsening of Modified National Institutes of Health Stroke Score)|(worsening of Modified NIH Stroke Scale)|(worsening of Modified NIH Stroke Score)|(worsening of National Institutes of Health Stroke Scale)|(worsening of National Institutes of Health Stroke Score)|(worsening of NIH Stroke Scale)|(worsening of NIH Stroke Score)|(worsening of NIHSS)|(worsening of ONS)|(worsening of Orgogozo Neurological Scale)|(worsening of Orgogozo Neurological Score)|(worsening of Scandinavian Stroke Scale)|(worsening of Scandinavian Stroke Score)|(worsening of SSS)|(worsening of Toronto Stroke Scale)|(worsening of Toronto Stroke Score)|(worsening of TSS)|(1-week death)|(1-week deceased)|(1-week died)|(1-week dying)|(1-week fatality)|(1-week mortality)|(1wk death)|(1-wk death)|(1wk deceased)|(1-wk deceased)|(1wk died)|(1-wk died)|(1wk dying)|(1-wk dying)|(1wk fatality)|(1-wk fatality)|(1wk mortality)|(1-wk mortality)|(7 days death)|(7 days deceased)|(7 days died)|(7 days dying)|(7 days fatality)|(7 days mortality)|(7days death)|(7days deceased)|(7days died)|(7days dying)|(7days fatality)|(7days mortality)|(7th day death)|(7th day deceased)|(7th day died)|(7th day dying)|(7th day fatality)|(7th day mortality)|(death ahead of the 2nd week)|(death ahead of the second week)|(death at 1 week)|(death at 168 hours)|(death at 168 hrs)|(death at 168hrs)|(death at one week)|(death at the 168th hour)|(death at the 168th hr)|(death at the 168thhr)|(death at the 1st week)|(death at the eighth day)|(death at the first week)|(death before 8 days)|(death before the 2nd week)|(death before the 8th day)|(death before the eighth day)|(death before the second week)|(death by 1 week)|(death by one week)|(death by the 2nd week)|(death by the end of the 1st week)|(death by the end of the first week)|(death by the second week)|(death during 0 to 7 days)|(death during 0 to seven days )|(death during 1 week )|(death during 168hrs)|(death during 1st 7 days)|(death during advance of the 2nd week)|(death during advance of the second week)|(death during one hundred and sixty eight hours)|(death during one week)|(death during one-week)|(death during one-wk)|(death during seven days)|(death during the 1st 168 hours)|(death during the 1st 168 hrs)|(death during the 1st 168hrs)|(death during the 1st seven days )|(death during the 1st week)|(death during the first 168 hours)|(death during the first 168hrs)|(death during the first 7 days)|(death during the first seven days)|(death during the first week)|(death during zero to 7 days)|(death during zero to seven days)|(death earlier than the 2nd week)|(death earlier than the second week)|(death equal to 7 days)|(death in 0 to 7 days)|(death in 0 to seven days )|(death in 1 week )|(death in 168 hrs)|(death in 168hrs)|(death in 1st 7 days)|(death in advance of the 2nd week)|(death in advance of the second week)|(death in one hundred and sixty eight hours)|(death in one week)|(death in one-week)|(death in one-wk)|(death in seven days)|(death in the 1st 168 hours)|(death in the 1st 168hrs)|(death in the 1st seven days )|(death in the 1st week)|(death in the first 168 hours)|(death in the first 168 hrs)|(death in the first 168hrs)|(death in the first 7 days)|(death in the first seven days)|(death in the first week)|(death in zero to 7 days)|(death in zero to seven days)|(death less than 1 week)|(death less than 169 hours)|(death less than 8 days)|(death less than one week)|(death less than or equal to 7 days)|(death less than or equal to seven days)|(death less than the 1st week)|(death less than the first week)|(death on the 7th day)|(death on the seventh day)|(death over 1 week)|(death over one week)|(death previous to the 2nd week)|(death previous to the second week)|(death prior to the 2nd week)|(death prior to the second week)|(death sooner than the 2nd week)|(death sooner than the second week)|(death up to 7 days)|(death up to day 7)|(death up to day seven)|(death up to seven days)|(death up to the 7th day)|(death up to the seventh day)|(death within 0 to 7 days)|(death within 0 to seven days )|(death within 1 week )|(death within 168 hours)|(death within 168 hrs)|(death within 168hrs)|(death within 1st 7 days)|(death within 7 days)|(death within a week)|(death within one hundred and sixty eight hours)|(death within one week)|(death within one-week)|(death within one-wk)|(death within seven days)|(death within the 1st 168 hours)|(death within the 1st 168 hrs)|(death within the 1st 168hrs)|(death within the 1st seven days)|(death within the 1st week)|(death within the first 168 hours)|(death within the first 168hrs)|(death within the first 7 days)|(death within the first seven days)|(death within the first week)|(death within zero to 7 days)|(death within zero to seven days)|(deceased ahead of the 2nd week)|(deceased ahead of the second week)|(deceased at 1 week)|(deceased at 168 hours)|(deceased at 168 hrs)|(deceased at 168hrs)|(deceased at one week)|(deceased at the 168th hour)|(deceased at the 168th hr)|(deceased at the 168thhr)|(deceased at the 1st week)|(deceased at the eighth day)|(deceased at the first week)|(deceased before 8 days)|(deceased before the 2nd week)|(deceased before the 8th day)|(deceased before the eighth day)|(deceased before the second week)|(deceased by 1 week)|(deceased by one week)|(deceased by the 2nd week)|(deceased by the end of the 1st week)|(deceased by the end of the first week)|(deceased by the second week)|(deceased during 0 to 7 days)|(deceased during 0 to seven days )|(deceased during 1 week)|(deceased during 1 week )|(deceased during 168 hrs)|(deceased during 168hrs)|(deceased during 1st 7 days)|(deceased during advance of the 2nd week)|(deceased during advance of the second week)|(deceased during one hundred and sixty eight hours)|(deceased during one week)|(deceased during one-week)|(deceased during one-wk)|(deceased during seven days)|(deceased during the 1st 168 hours)|(deceased during the 1st 168 hrs)|(deceased during the 1st 168hrs)|(deceased during the 1st seven days )|(deceased during the 1st week)|(deceased during the first 168 hours)|(deceased during the first 168 hrs)|(deceased during the first 168hrs)|(deceased during the first 7 days)|(deceased during the first seven days)|(deceased during the first week)|(deceased during zero to 7 days)|(deceased during zero to seven days)|(deceased earlier than the 2nd week)|(deceased earlier than the second week)|(deceased equal to 7 days)|(deceased in 0 to 7 days)|(deceased in 0 to seven days)|(deceased in 1 week)|(deceased in 1 week )|(deceased in 168 hrs)|(deceased in 168hrs)|(deceased in 1st 7 days)|(deceased in advance of the 2nd week)|(deceased in advance of the second week)|(deceased in one hundred and sixty eight hours)|(deceased in one week)|(deceased in one-week)|(deceased in one-wk)|(deceased in seven days)|(deceased in the 1st 168 hours)|(deceased in the 1st 168 hrs)|(deceased in the 1st 168hrs)|(deceased in the 1st seven days )|(deceased in the 1st week)|(deceased in the first 168 hours)|(deceased in the first 168 hrs)|(deceased in the first 168hrs)|(deceased in the first 7 days)|(deceased in the first seven days)|(deceased in the first week)|(deceased in zero to 7 days)|(deceased in zero to seven days)|(deceased less than 1 week)|(deceased less than 169 hours)|(deceased less than 8 days)|(deceased less than one week)|(deceased less than or equal to 7 days)|(deceased less than or equal to seven days)|(deceased less than the 1st week)|(deceased less than the first week)|(deceased on the 7th day)|(deceased on the seventh day)|(deceased over 1 week)|(deceased over one week)|(deceased previous to the 2nd week)|(deceased previous to the second week)|(deceased prior to the 2nd week)|(deceased prior to the second week)|(deceased sooner than the 2nd week)|(deceased sooner than the second week)|(deceased up to 7 days)|(deceased up to day 7)|(deceased up to day seven)|(deceased up to seven days)|(deceased up to the 7th day)|(deceased up to the seventh day)|(deceased within 0 to 7 days)|(deceased within 0 to seven days )|(deceased within 1 week)|(deceased within 1 week )|(deceased within 168 hours)|(deceased within 168 hrs)|(deceased within 168hrs)|(deceased within 1st 7 days)|(deceased within 7 days)|(deceased within a week)|(deceased within one hundred and sixty eight hours)|(deceased within one week)|(deceased within one-week)|(deceased within one-wk)|(deceased within seven days)|(deceased within the 1st 168 hours)|(deceased within the 1st 168 hrs)|(deceased within the 1st 168hrs)|(deceased within the 1st seven days)|(deceased within the 1st week)|(deceased within the first 168 hours)|(deceased within the first 168 hrs)|(deceased within the first 168hrs)|(deceased within the first 7 days)|(deceased within the first seven days)|(deceased within the first week)|(deceased within zero to 7 days)|(deceased within zero to seven days)|(died ahead of the 2nd week)|(died ahead of the second week)|(died at 1 week)|(died at 168 hours)|(died at 168 hrs)|(died at 168hrs)|(died at one week)|(died at the 168th hour)|(died at the 168th hr)|(died at the 168thhr )|(died at the 1st week)|(died at the eighth day)|(died at the first week)|(died before 8 days)|(died before the 2nd week)|(died before the 8th day)|(died before the eighth day)|(died before the second week)|(died by 1 week)|(died by one week)|(died by the 2nd week)|(died by the end of the 1st week)|(died by the end of the first week)|(died by the second week)|(died during 0 to 7 days)|(died during 0 to seven days )|(died during 1 week)|(died during 1 week )|(died during 168 hrs)|(died during 168hrs)|(died during 1st 7 days)|(died during advance of the 2nd week)|(died during advance of the second week)|(died during one hundred and sixty eight hours)|(died during one week)|(died during one-week)|(died during one-wk)|(died during seven days)|(died during the 1st 168 hours)|(died during the 1st 168 hrs)|(died during the 1st 168hrs)|(died during the 1st seven days )|(died during the 1st week)|(died during the first 168 hours)|(died during the first 168 hrs)|(died during the first 168hrs)|(died during the first 7 days)|(died during the first seven days)|(died during the first week)|(died during zero to 7 days)|(died during zero to seven days)|(died earlier than the 2nd week)|(died earlier than the second week)|(died equal to 7 days)|(died in 0 to 7 days)|(died in 0 to seven days )|(died in 1 week)|(died in 1 week )|(died in 168 hrs)|(died in 168hrs)|(died in 1st 7 days)|(died in advance of the 2nd week)|(died in advance of the second week)|(died in one hundred and sixty eight hours)|(died in one week)|(died in one-week)|(died in one-wk)|(died in seven days)|(died in the 1st 168 hours)|(died in the 1st 168 hrs)|(died in the 1st 168hrs)|(died in the 1st seven days )|(died in the 1st week)|(died in the first 168 hours)|(died in the first 168 hrs)|(died in the first 168hrs)|(died in the first 7 days)|(died in the first seven days)|(died in the first week)|(died in zero to 7 days)|(died in zero to seven days)|(died less than 1 week)|(died less than 169 hours)|(died less than 8 days)|(died less than one week)|(died less than or equal to 7 days)|(died less than or equal to seven days)|(died less than the 1st week)|(died less than the first week)|(died on the 7th day)|(died on the seventh day)|(died over 1 week)|(died over one week)|(died previous to the 2nd week)|(died previous to the second week)|(died prior to the 2nd week)|(died prior to the second week)|(died sooner than the 2nd week)|(died sooner than the second week)|(died up to 7 days)|(died up to day 7)|(died up to day seven)|(died up to seven days)|(died up to the 7th day)|(died up to the seventh day)|(died within 0 to 7 days)|(died within 0 to seven days )|(died within 1 week)|(died within 1 week )|(died within 168 hours)|(died within 168 hrs)|(died within 168hrs)|(died within 1st 7 days)|(died within 7 days)|(died within a week)|(died within one hundred and sixty eight hours)|(died within one week)|(died within one-week)|(died within one-wk)|(died within seven days)|(died within the 1st 168 hours)|(died within the 1st 168 hrs)|(died within the 1st 168hrs)|(died within the 1st seven days)|(died within the 1st week)|(died within the first 168 hours)|(died within the first 168 hrs)|(died within the first 168hrs)|(died within the first 7 days)|(died within the first seven days)|(died within the first week)|(died within zero to 7 days)|(died within zero to seven days)|(dying ahead of the 2nd week)|(dying ahead of the second week)|(dying at 1 week)|(dying at 168 hours)|(dying at 168 hrs)|(dying at 168hrs)|(dying at one week)|(dying at the 168th hour)|(dying at the 168th hr)|(dying at the 168thhr )|(dying at the 1st week)|(dying at the eighth day)|(dying at the first week)|(dying before 8 days)|(dying before the 2nd week)|(dying before the 8th day)|(dying before the eighth day)|(dying before the second week)|(dying by 1 week)|(dying by one week)|(dying by the 2nd week)|(dying by the end of the first week)|(dying by the second week)|(dying during 0 to 7 days)|(dying during 0 to seven days )|(dying during 1 week)|(dying during 1 week )|(dying during 168 hrs)|(dying during 168hrs)|(dying during 1st 7 days)|(dying during advance of the 2nd week)|(dying during advance of the second week)|(dying during one hundred and sixty eight hours)|(dying during one week)|(dying during one-week)|(dying during one-wk)|(dying during seven days)|(dying during the 1st 168 hours)|(dying during the 1st 168 hrs)|(dying during the 1st 168hrs)|(dying during the 1st seven days )|(dying during the 1st week)|(dying during the first 168 hours)|(dying during the first 168 hrs)|(dying during the first 168hrs)|(dying during the first 7 days)|(dying during the first seven days)|(dying during the first week)|(dying during zero to 7 days)|(dying during zero to seven days)|(dying earlier than the 2nd week)|(dying earlier than the second week)|(dying equal to 7 days)|(dying in 0 to 7 days)|(dying in 0 to seven days )|(dying in 1 week)|(dying in 1 week)|(dying in 168 hrs)|(dying in 168hrs)|(dying in 1st 7 days)|(dying in advance of the 2nd week)|(dying in advance of the second week)|(dying in one hundred and sixty eight hours)|(dying in one week)|(dying in one-week)|(dying in one-wk)|(dying in seven days)|(dying in the 1st 168 hours)|(dying in the 1st 168 hrs)|(dying in the 1st 168hrs)|(dying in the 1st seven days )|(dying in the 1st week)|(dying in the first 168 hours)|(dying in the first 168 hrs)|(dying in the first 168hrs)|(dying in the first 7 days)|(dying in the first seven days)|(dying in the first week)|(dying in zero to 7 days)|(dying in zero to seven days)|(dying less than 1 week)|(dying less than 169 hours)|(dying less than 8 days)|(dying less than one week)|(dying less than or equal to 7 days)|(dying less than or equal to seven days)|(dying less than the 1st week)|(dying less than the first week)|(dying on the 7th day)|(dying on the seventh day)|(dying over 1 week)|(dying over one week)|(dying previous to the 2nd week)|(dying previous to the second week)|(dying prior to the 2nd week)|(dying prior to the second week)|(dying sooner than the 2nd week)|(dying sooner than the second week)|(dying up to 7 days)|(dying up to day 7)|(dying up to day seven)|(dying up to seven days)|(dying up to the 7th day)|(dying up to the seventh day)|(dying within 0 to 7 days)|(dying within 0 to seven days)|(dying within 1 week)|(dying within 1 week )|(dying within 168 hours)|(dying within 168 hrs)|(dying within 168hrs)|(dying within 1st 7 days)|(dying within 7 days)|(dying within a week)|(dying within one hundred and sixty eight hours)|(dying within one week)|(dying within one-week)|(dying within one-wk)|(dying within seven days)|(dying within the 1st 168 hours)|(dying within the 1st 168 hrs)|(dying within the 1st 168hrs)|(dying within the 1st seven days)|(dying within the 1st week)|(dying within the first 168 hours)|(dying within the first 168 hrs)|(dying within the first 168hrs)|(dying within the first 7 days)|(dying within the first seven days)|(dying within the first week)|(dying within zero to 7 days)|(dying within zero to seven days)|(fatality ahead of the 2nd week)|(fatality ahead of the second week)|(fatality at 1 week)|(fatality at 168 hours)|(fatality at 168 hrs)|(fatality at 168hrs)|(fatality at one week)|(fatality at the 168th hour)|(fatality at the 168th hr)|(fatality at the 168thhr )|(fatality at the 1st week)|(fatality at the eighth day)|(fatality at the first week)|(fatality before 8 days)|(fatality before the 2nd week)|(fatality before the 8th day)|(fatality before the eighth day)|(fatality before the second week)|(fatality by 1 week)|(fatality by one week)|(fatality by the 2nd week)|(fatality by the end of the 1st week)|(fatality by the end of the first week)|(fatality by the second week)|(fatality during 0 to 7 days)|(fatality during 0 to seven days )|(fatality during 1 week)|(fatality during 1 week )|(fatality during 168 hrs)|(fatality during 168hrs)|(fatality during 1st 7 days)|(fatality during advance of the 2nd week)|(fatality during advance of the second week)|(fatality during one hundred and sixty eight hours)|(fatality during one week)|(fatality during one-week)|(fatality during one-wk)|(fatality during seven days)|(fatality during the 1st 168 hours)|(fatality during the 1st 168 hrs)|(fatality during the 1st 168hrs)|(fatality during the 1st seven days )|(fatality during the 1st week)|(fatality during the first 168 hours)|(fatality during the first 168 hrs)|(fatality during the first 168hrs)|(fatality during the first 7 days)|(fatality during the first seven days)|(fatality during the first week)|(fatality during zero to 7 days)|(fatality during zero to seven days)|(fatality earlier than the 2nd week)|(fatality earlier than the second week)|(fatality equal to 7 days)|(fatality in 0 to 7 days)|(fatality in 0 to seven days )|(fatality in 1 week)|(fatality in 1 week )|(fatality in 168 hrs)|(fatality in 168hrs)|(fatality in 1st 7 days)|(fatality in advance of the 2nd week)|(fatality in advance of the second week)|(fatality in one hundred and sixty eight hours)|(fatality in one week)|(fatality in one-week)|(fatality in one-wk)|(fatality in seven days)|(fatality in the 1st 168 hours)|(fatality in the 1st 168 hrs)|(fatality in the 1st 168hrs)|(fatality in the 1st seven days )|(fatality in the 1st week)|(fatality in the first 168 hours)|(fatality in the first 168 hrs)|(fatality in the first 168hrs)|(fatality in the first 7 days)|(fatality in the first seven days)|(fatality in the first week)|(fatality in zero to 7 days)|(fatality in zero to seven days)|(fatality less than 1 week)|(fatality less than 169 hours)|(fatality less than 8 days)|(fatality less than one week)|(fatality less than or equal to 7 days)|(fatality less than or equal to seven days)|(fatality less than the 1st week)|(fatality less than the first week)|(fatality on the 7th day)|(fatality on the seventh day)|(fatality over 1 week)|(fatality over one week)|(fatality previous to the 2nd week)|(fatality previous to the second week)|(fatality prior to the 2nd week)|(fatality prior to the second week)|(fatality sooner than the 2nd week)|(fatality sooner than the second week)|(fatality up to 7 days)|(fatality up to day 7)|(fatality up to day seven)|(fatality up to seven days)|(fatality up to the 7th day)|(fatality up to the seventh day)|(fatality within 0 to 7 days)|(fatality within 0 to seven days )|(fatality within 1 week)|(fatality within 1 week )|(fatality within 168 hours)|(fatality within 168 hrs)|(fatality within 168hrs)|(fatality within 1st 7 days)|(fatality within 7 days)|(fatality within a week)|(fatality within one hundred and sixty eight hours)|(fatality within one week)|(fatality within one-week)|(fatality within one-wk)|(fatality within seven days)|(fatality within the 1st 168 hours)|(fatality within the 1st 168 hrs)|(fatality within the 1st 168hrs)|(fatality within the 1st seven days)|(fatality within the 1st week)|(fatality within the first 168 hours)|(fatality within the first 168 hrs)|(fatality within the first 168hrs)|(fatality within the first 7 days)|(fatality within the first seven days)|(fatality within the first week)|(fatality within zero to 7 days)|(fatality within zero to seven days)|(mortality ahead of the 2nd week)|(mortality ahead of the second wee)|(mortality at 1 week)|(mortality at 168 hours)|(mortality at 168 hrs)|(mortality at 168hrs)|(mortality at one week)|(mortality at the 168th hour)|(mortality at the 168th hr)|(mortality at the 168thhr )|(mortality at the 1st week)|(mortality at the eighth day)|(mortality at the first week)|(mortality before 8 days)|(mortality before the 2nd week)|(mortality before the 8th day)|(mortality before the eighth day)|(mortality before the second week)|(mortality by 1 week)|(mortality by one week)|(mortality by the 2nd week)|(mortality by the end of the 1st week)|(mortality by the end of the first week)|(mortality by the second week)|(mortality during 0 to 7 days)|(mortality during 0 to seven days)|(mortality during 1 week)|(mortality during 1 week )|(mortality during 168 hrs)|(mortality during 168hrs)|(mortality during 1st 7 days)|(mortality during advance of the 2nd week)|(mortality during advance of the second week)|(mortality during one hundred and sixty eight hours)|(mortality during one week)|(mortality during one-week)|(mortality during one-wk)|(mortality during seven days)|(mortality during the 1st 168 hours)|(mortality during the 1st 168 hrs)|(mortality during the 1st 168hrs)|(mortality during the 1st seven days )|(mortality during the 1st week)|(mortality during the first 168 hours)|(mortality during the first 168 hrs)|(mortality during the first 168hrs)|(mortality during the first 7 days)|(mortality during the first seven days)|(mortality during the first week)|(mortality during zero to 7 days)|(mortality during zero to seven days)|(mortality earlier than the 2nd week)|(mortality earlier than the second week)|(mortality equal to 7 days)|(mortality in 0 to 7 days)|(mortality in 0 to seven days)|(mortality in 1 week)|(mortality in 1 week )|(mortality in 168 hrs)|(mortality in 168hrs)|(mortality in 1st 7 days)|(mortality in advance of the 2nd week)|(mortality in advance of the second week)|(mortality in one hundred and sixty eight hours)|(mortality in one week)|(mortality in one-week)|(mortality in one-wk)|(mortality in seven days)|(mortality in the 1st 168 hours)|(mortality in the 1st 168 hrs)|(mortality in the 1st 168hrs)|(mortality in the 1st seven days )|(mortality in the 1st week)|(mortality in the first 168 hours)|(mortality in the first 168 hrs)|(mortality in the first 168hrs)|(mortality in the first 7 days)|(mortality in the first seven days)|(mortality in the first week)|(mortality in zero to 7 days)|(mortality in zero to seven days)|(mortality less than 1 week)|(mortality less than 169 hours)|(mortality less than 8 days)|(mortality less than one week)|(mortality less than or equal to 7 days)|(mortality less than or equal to seven days)|(mortality less than the 1st week)|(mortality less than the first week)|(mortality on the 7th day)|(mortality on the seventh day)|(mortality over 1 week)|(mortality over one week)|(mortality previous to the 2nd week)|(mortality previous to the second week)|(mortality prior to the 2nd week)|(mortality prior to the second week)|(mortality sooner than the 2nd week)|(mortality sooner than the second week)|(mortality up to 7 days)|(mortality up to day 7)|(mortality up to day seven)|(mortality up to seven days)|(mortality up to the 7th day)|(mortality up to the seventh day)|(mortality within 0 to 7 days)|(mortality within 0 to seven days )|(mortality within 1 week)|(mortality within 1 week )|(mortality within 168 hours)|(mortality within 168 hrs)|(mortality within 168hrs)|(mortality within 1st 7 days)|(mortality within 7 days)|(mortality within a week)|(mortality within one hundred and sixty eight hours)|(mortality within one week)|(mortality within one-week)|(mortality within one-wk)|(mortality within seven days)|(mortality within the 1st 168 hours)|(mortality within the 1st 168 hrs)|(mortality within the 1st 168hrs)|(mortality within the 1st seven days)|(mortality within the 1st week)|(mortality within the first 168 hours)|(mortality within the first 168 hrs)|(mortality within the first 168hrs)|(mortality within the first 7 days)|(mortality within the first seven days)|(mortality within the first week)|(mortality within zero to 7 days)|(mortality within zero to seven days)|(mortality sooner than the first week)|(mortality sooner than the 1^st^ week)|(mortality sooner than the 1^st^ wk)|(mortality sooner than 7 days)|(mortality sooner than seven days)|(mortality sooner than the 7^th^ day)|(mortality sooner than the seventh day)|(24 hour mortality)|(24-hour mortality)|(24-hr mortality)|(24hr mortality)|(24 hr mortality)|(twenty four hour mortality)|(twenty-four hour mortality)|(mortality at one day)|(mortality at 1 day)|(mortality in one day)|(mortality in 1 day)|(mortality at 24 hours)|(mortality in 24 hours)|(mortality in twenty four hours)|(mortality in twenty-four hours)|(mortality at twenty-four hours)|(mortality at twenty four hours)|(48 hour mortality)|(48-hour mortality)|(48-hr mortality)|(48hr mortality)|(48 hr mortality)|(forty eight hour mortality)|(forty-eight hour mortality)|(mortality at two days)|(mortality at 2 days)|(mortality in two days)|(mortality in 2 days)|(mortality at 48 hours)|(mortality in 48 hours)|(mortality in forty eight hours)|(mortality in forty-eight hours)|(mortality at forty eight hours)|(mortality at forty-eight hours)|(72 hour mortality)|(72-hour mortality)|(72-hr mortality)|(72hr mortality)|(72 hr mortality)|(seventy two hour mortality)|(seventy-two hour mortality)|(mortality at three days)|(mortality at 3 days)|(mortality in three days)|(mortality in 3 days)|(mortality at 72 hours)|(mortality in 72 hours)|(mortality in seventy two hours)|(mortality in seventy-two hours)|(mortality at seventy two hours)|(mortality at seventy-two hours)|(96 hour mortality)|(96-hour mortality)|(96-hr mortality)|(96hr mortality)|(96 hr mortality)|(ninety six hour mortality)|(ninety-six hour mortality)|(mortality at 4 days)|(mortality at 4 days)|(mortality in four days)|(mortality in 4 days)|(mortality at 96 hours)|(mortality in 96 hours)|(mortality in ninety six hours)|(mortality in ninety-six hours)|(mortality at ninety six hours)|(mortality at ninety-six hours)|(120 hour mortality)|(120-hour mortality)|(120-hr mortality)|(120hr mortality)|(120 hr mortality)|(one hundred and twenty hour mortality)|(mortality at five days)|(mortality at 5 days)|(mortality in five days)|(mortality in 5 days)|(mortality at one hundred and twenty hours)|(mortality in one hundred and twenty hours)|(mortality at one hundred and twenty hours)|(144 hour mortality)|(144-hour mortality)|(144-hr mortality)|(144hr mortality)|(144 hr mortality)|(one hundred and forty four hour mortality)|(one hundred and forty-four hour mortality)|(mortality at six days)|(mortality at 6 days)|(mortality in six days)|(mortality in 6 days)|(mortality at 144 hours)|(mortality in 144 hours)|(mortality in one hundred and forty four hours)|(mortality in one hundred and forty-four hours)|(mortality at one hundred and forty four hours)|(mortality at one hundred and forty-four hours)|(168 hour mortality)|(168-hour mortality)|(168-hr mortality)|(168hr mortality)|(168 hr mortality)|(one hundred and sixty eight hour mortality)|(one hundred and sixty-eight hour mortality)|(mortality at seven days)|(mortality at 7 days)|(mortality in seven days)|(mortality in 7 days)|(mortality at 168 hours)|(mortality in 168 hours)|(mortality in one hundred and sixty eight hours)|(mortality in one hundred and sixty-eight hours)|(mortality at one hundred and sixty eight hours)|(mortality at one hundred and sixty-eight hours)|(fatality sooner than the first week)|(fatality sooner than the 1^st^ week)|(fatality sooner than the 1^st^ wk)|(fatality sooner than 7 days)|(fatality sooner than seven days)|(fatality sooner than the 7^th^ day)|(fatality sooner than the seventh day)|(24 hour fatality)|(24-hour fatality)|(24-hr fatality)|(24hr fatality)|(24 hr fatality)|(twenty four hour fatality)|(twenty-four hour fatality)|(fatality at one day)|(fatality at 1 day)|(fatality in one day)|(fatality in 1 day)|(fatality at 24 hours)|(fatality in 24 hours)|(fatality in twenty four hours)|(fatality in twenty-four hours)|(fatality at twenty-four hours)|(fatality at twenty four hours)|(48 hour fatality)|(48-hour fatality)|(48-hr fatality)|(48hr fatality)|(48 hr fatality)|(forty eight hour fatality)|(forty-eight hour fatality)|(fatality at two days)|(fatality at 2 days)|(fatality in two days)|(fatality in 2 days)|(fatality at 48 hours)|(fatality in 48 hours)|(fatality in forty eight hours)|(fatality in forty-eight hours)|(fatality at forty eight hours)|(fatality at forty-eight hours)|(72 hour fatality)|(72-hour fatality)|(72-hr fatality)|(72hr fatality)|(72 hr fatality)|(seventy two hour fatality)|(seventy-two hour fatality)|(fatality at three days)|(fatality at 3 days)|(fatality in three days)|(fatality in 3 days)|(fatality at 72 hours)|(fatality in 72 hours)|(fatality in seventy two hours)|(fatality in seventy-two hours)|(fatality at seventy two hours)|(fatality at seventy-two hours)|(96 hour fatality)|(96-hour fatality)|(96-hr fatality)|(96hr fatality)|(96 hr fatality)|(ninety six hour fatality)|(ninety-six hour fatality)|(fatality at 4 days)|(fatality at 4 days)|(fatality in four days)|(fatality in 4 days)|(fatality at 96 hours)|(fatality in 96 hours)|(fatality in ninety six hours)|(fatality in ninety-six hours)|(fatality at ninety six hours)|(fatality at ninety-six hours)|(120 hour fatality)|(120-hour fatality)|(120-hr fatality)|(120hr fatality)|(120 hr fatality)|(one hundred and twenty hour fatality)|(fatality at five days)|(fatality at 5 days)|(fatality in five days)|(fatality in 5 days)|(fatality at one hundred and twenty hours)|(fatality in one hundred and twenty hours)|(fatality at one hundred and twenty hours)|(144 hour fatality)|(144-hour fatality)|(144-hr fatality)|(144hr fatality)|(144 hr fatality)|(one hundred and forty four hour fatality)|(one hundred and forty-four hour fatality)|(fatality at six days)|(fatality at 6 days)|(fatality in six days)|(fatality in 6 days)|(fatality at 144 hours)|(fatality in 144 hours)|(fatality in one hundred and forty four hours)|(fatality in one hundred and forty-four hours)|(fatality at one hundred and forty four hours)|(fatality at one hundred and forty-four hours)|(168 hour fatality)|(168-hour fatality)|(168-hr fatality)|(168hr fatality)|(168 hr fatality)|(one hundred and sixty eight hour fatality)|(one hundred and sixty-eight hour fatality)|(fatality at seven days)|(fatality at 7 days)|(fatality in seven days)|(fatality in 7 days)|(fatality at 168 hours)|(fatality in 168 hours)|(fatality in one hundred and sixty eight hours)|(fatality in one hundred and sixty-eight hours)|(fatality at one hundred and sixty eight hours)|(fatality at one hundred and sixty-eight hours)|(death sooner than the first week)|(death sooner than the 1^st^ week)|(death sooner than the 1^st^ wk)|(death sooner than 7 days)|(death sooner than seven days)|(death sooner than the 7^th^ day)|(death sooner than the seventh day)|(24 hour death)|(24-hour death)|(24-hr death)|(24hr death)|(24 hr death)|(twenty four hour death)|(twenty-four hour death)|(death at one day)|(death at 1 day)|(death in one day)|(death in 1 day)|(death at 24 hours)|(death in 24 hours)|(death in twenty four hours)|(death in twenty-four hours)|(death at twenty-four hours)|(death at twenty four hours)|(48 hour death)|(48-hour death)|(48-hr death)|(48hr death)|(48 hr death)|(forty eight hour death)|(forty-eight hour death)|(death at two days)|(death at 2 days)|(death in two days)|(death in 2 days)|(death at 48 hours)|(death in 48 hours)|(death in forty eight hours)|(death in forty-eight hours)|(death at forty eight hours)|(death at forty-eight hours)|(72 hour death)|(72-hour death)|(72-hr death)|(72hr death)|(72 hr death)|(seventy two hour death)|(seventy-two hour death)|(death at three days)|(death at 3 days)|(death in three days)|(death in 3 days)|(death at 72 hours)|(death in 72 hours)|(death in seventy two hours)|(death in seventy-two hours)|(death at seventy two hours)|(death at seventy-two hours)|(96 hour death)|(96-hour death)|(96-hr death)|(96hr death)|(96 hr death)|(ninety six hour death)|(ninety-six hour death)|(death at 4 days)|(death at 4 days)|(death in four days)|(death in 4 days)|(death at 96 hours)|(death in 96 hours)|(death in ninety six hours)|(death in ninety-six hours)|(death at ninety six hours)|(death at ninety-six hours)|(120 hour death)|(120-hour death)|(120-hr death)|(120hr death)|(120 hr death)|(one hundred and twenty hour death)|(death at five days)|(death at 5 days)|(death in five days)|(death in 5 days)|(death at one hundred and twenty hours)|(death in one hundred and twenty hours)|(death at one hundred and twenty hours)|(144 hour death)|(144-hour death)|(144-hr death)|(144hr death)|(144 hr death)|(one hundred and forty four hour death)|(one hundred and forty-four hour death)|(death at six days)|(death at 6 days)|(death in six days)|(death in 6 days)|(death at 144 hours)|(death in 144 hours)|(death in one hundred and forty four hours)|(death in one hundred and forty-four hours)|(death at one hundred and forty four hours)|(death at one hundred and forty-four hours)|(168 hour death)|(168-hour death)|(168-hr death)|(168hr death)|(168 hr death)|(one hundred and sixty eight hour death)|(one hundred and sixty-eight hour death)|(death at seven days)|(death at 7 days)|(death in seven days)|(death in 7 days)|(death at 168 hours)|(death in 168 hours)|(death in one hundred and sixty eight hours)|(death in one hundred and sixty-eight hours)|(death at one hundred and sixty eight hours)|(death at one hundred and sixty-eight hours)|(died sooner than the first week)|(died sooner than the 1^st^ week)|(died sooner than the 1^st^ wk)|(died sooner than 7 days)|(died sooner than seven days)|(died sooner than the 7^th^ day)|(died sooner than the seventh day)|(24 hour died)|(24-hour died)|(24-hr died)|(24hr died)|(24 hr died)|(twenty four hour died)|(twenty-four hour died)|(died at one day)|(died at 1 day)|(died in one day)|(died in 1 day)|(died at 24 hours)|(died in 24 hours)|(died in twenty four hours)|(died in twenty-four hours)|(died at twenty-four hours)|(died at twenty four hours)|(48 hour died)|(48-hour died)|(48-hr died)|(48hr died)|(48 hr died)|(forty eight hour died)|(forty-eight hour died)|(died at two days)|(died at 2 days)|(died in two days)|(died in 2 days)|(died at 48 hours)|(died in 48 hours)|(died in forty eight hours)|(died in forty-eight hours)|(died at forty eight hours)|(died at forty-eight hours)|(72 hour died)|(72-hour died)|(72-hr died)|(72hr died)|(72 hr died)|(seventy two hour died)|(seventy-two hour died)|(died at three days)|(died at 3 days)|(died in three days)|(died in 3 days)|(died at 72 hours)|(died in 72 hours)|(died in seventy two hours)|(died in seventy-two hours)|(died at seventy two hours)|(died at seventy-two hours)|(96 hour died)|(96-hour died)|(96-hr died)|(96hr died)|(96 hr died)|(ninety six hour died)|(ninety-six hour died)|(died at 4 days)|(died at 4 days)|(died in four days)|(died in 4 days)|(died at 96 hours)|(died in 96 hours)|(died in ninety six hours)|(died in ninety-six hours)|(died at ninety six hours)|(died at ninety-six hours)|(120 hour died)|(120-hour died)|(120-hr died)|(120hr died)|(120 hr died)|(one hundred and twenty hour died)|(died at five days)|(died at 5 days)|(died in five days)|(died in 5 days)|(died at one hundred and twenty hours)|(died in one hundred and twenty hours)|(died at one hundred and twenty hours)|(144 hour died)|(144-hour died)|(144-hr died)|(144hr died)|(144 hr died)|(one hundred and forty four hour died)|(one hundred and forty-four hour died)|(died at six days)|(died at 6 days)|(died in six days)|(died in 6 days)|(died at 144 hours)|(died in 144 hours)|(died in one hundred and forty four hours)|(died in one hundred and forty-four hours)|(died at one hundred and forty four hours)|(died at one hundred and forty-four hours)|(168 hour died)|(168-hour died)|(168-hr died)|(168hr died)|(168 hr died)|(one hundred and sixty eight hour died)|(one hundred and sixty-eight hour died)|(died at seven days)|(died at 7 days)|(died in seven days)|(died in 7 days)|(died at 168 hours)|(died in 168 hours)|(died in one hundred and sixty eight hours)|(died in one hundred and sixty-eight hours)|(died at one hundred and sixty eight hours)|(died at one hundred and sixty-eight hours)|(deceased sooner than the first week)|(deceased sooner than the 1^st^ week)|(deceased sooner than the 1^st^ wk)|(deceased sooner than 7 days)|(deceased sooner than seven days)|(deceased sooner than the 7^th^ day)|(deceased sooner than the seventh day)|(24 hour deceased)|(24-hour deceased)|(24-hr deceased)|(24hr deceased)|(24 hr deceased)|(twenty four hour deceased)|(twenty-four hour deceased)|(deceased at one day)|(deceased at 1 day)|(deceased in one day)|(deceased in 1 day)|(deceased at 24 hours)|(deceased in 24 hours)|(deceased in twenty four hours)|(deceased in twenty-four hours)|(deceased at twenty-four hours)|(deceased at twenty four hours)|(48 hour deceased)|(48-hour deceased)|(48-hr deceased)|(48hr deceased)|(48 hr deceased)|(forty eight hour deceased)|(forty-eight hour deceased)|(deceased at two days)|(deceased at 2 days)|(deceased in two days)|(deceased in 2 days)|(deceased at 48 hours)|(deceased in 48 hours)|(deceased in forty eight hours)|(deceased in forty-eight hours)|(deceased at forty eight hours)|(deceased at forty-eight hours)|(72 hour deceased)|(72-hour deceased)|(72-hr deceased)|(72hr deceased)|(72 hr deceased)|(seventy two hour deceased)|(seventy-two hour deceased)|(deceased at three days)|(deceased at 3 days)|(deceased in three days)|(deceased in 3 days)|(deceased at 72 hours)|(deceased in 72 hours)|(deceased in seventy two hours)|(deceased in seventy-two hours)|(deceased at seventy two hours)|(deceased at seventy-two hours)|(96 hour deceased)|(96-hour deceased)|(96-hr deceased)|(96hr deceased)|(96 hr deceased)|(ninety six hour deceased)|(ninety-six hour deceased)|(deceased at 4 days)|(deceased at 4 days)|(deceased in four days)|(deceased in 4 days)|(deceased at 96 hours)|(deceased in 96 hours)|(deceased in ninety six hours)|(deceased in ninety-six hours)|(deceased at ninety six hours)|(deceased at ninety-six hours)|(120 hour deceased)|(120-hour deceased)|(120-hr deceased)|(120hr deceased)|(120 hr deceased)|(one hundred and twenty hour deceased)|(deceased at five days)|(deceased at 5 days)|(deceased in five days)|(deceased in 5 days)|(deceased at one hundred and twenty hours)|(deceased in one hundred and twenty hours)|(deceased at one hundred and twenty hours)|(144 hour deceased)|(144-hour deceased)|(144-hr deceased)|(144hr deceased)|(144 hr deceased)|(one hundred and forty four hour deceased)|(one hundred and forty-four hour deceased)|(deceased at six days)|(deceased at 6 days)|(deceased in six days)|(deceased in 6 days)|(deceased at 144 hours)|(deceased in 144 hours)|(deceased in one hundred and forty four hours)|(deceased in one hundred and forty-four hours)|(deceased at one hundred and forty four hours)|(deceased at one hundred and forty-four hours)|(168 hour deceased)|(168-hour deceased)|(168-hr deceased)|(168hr deceased)|(168 hr deceased)|(one hundred and sixty eight hour deceased)|(one hundred and sixty-eight hour deceased)|(deceased at seven days)|(deceased at 7 days)|(deceased in seven days)|(deceased in 7 days)|(deceased at 168 hours)|(deceased in 168 hours)|(deceased in one hundred and sixty eight hours)|(deceased in one hundred and sixty-eight hours)|(deceased at one hundred and sixty eight hours)|(deceased at one hundred and sixty-eight hours)|(1-week death)|(1-week deceased)|(1-week died)|(1-week dying)|(1-week case fatality)|(1-week mortality)|(1wk death)|(1-wk death)|(1wk deceased)|(1-wk deceased)|(1wk died)|(1-wk died)|(1wk dying)|(1-wk dying)|(1wk case fatality)|(1-wk case fatality)|(1wk mortality)|(1-wk mortality)|(7 days death)|(7 days deceased)|(7 days died)|(7 days dying)|(7 days case fatality)|(7 days mortality)|(7days death)|(7days deceased)|(7days died)|(7days dying)|(7days case fatality)|(7days mortality)|(7th day death)|(7th day deceased)|(7th day died)|(7th day dying)|(7th day case fatality)|(7th day mortality)|(death ahead of the 2nd week)|(death ahead of the second week)|(death at 1 week)|(death at 168 hours)|(death at 168 hrs)|(death at 168hrs)|(death at one week)|(death at the 168th hour)|(death at the 168th hr)|(death at the 168thhr )|(death at the 1st week)|(death at the eighth day)|(death at the first week)|(death before 8 days)|(1-week death)|(1-week deceased)|(1-week died)|(1-week dying)|(1-week case-fatality)|(1-week mortality)|(1wk death)|(1-wk death)|(1wk deceased)|(1-wk deceased)|(1wk died)|(1-wk died)|(1wk dying)|(1-wk dying)|(1wk case-fatality)|(1-wk case-fatality)|(1wk mortality)|(1-wk mortality)|(7 days death)|(7 days deceased)|(7 days died)|(7 days dying)|(7 days case-fatality)|(7 days mortality)|(7days death)|(7days deceased)|(7days died)|(7days dying)|(7days case-fatality)|(7days mortality)|(7th day death)|(7th day deceased)|(7th day died)|(7th day dying)|(7th day case-fatality)|(7th day mortality)|(death ahead of the 2nd week)|(death ahead of the second week)|(death at 1 week)|(death at 168 hours)|(death at 168 hrs)|(death at 168hrs)|(death at one week)|(death at the 168th hour)|(death at the 168th hr)|(death at the 168thhr)|(death at the 1st week)|(death at the eighth day)|(death at the first week)|(death before 8 days)|(1-week death)|(1-week deceased)|(1-week died)|(1-week dying)|(1-week acute fatality)|(1-week mortality)|(1wk death)|(1-wk death)|(1wk deceased)|(1-wk deceased)|(1wk died)|(1-wk died)|(1wk dying)|(1-wk dying)|(1wk acute fatality)|(1-wk acute fatality)|(1wk mortality)|(1-wk mortality)|(7 days death)|(7 days deceased)|(7 days died)|(7 days dying)|(7 days acute fatality)|(7 days mortality)|(7days death)|(7days deceased)|(7days died)|(7days dying)|(7days acute fatality)|(7days mortality)|(7th day death)|(7th day deceased)|(7th day died)|(7th day dying)|(7th day acute fatality)|(7th day mortality)|(death ahead of the 2nd week)|(death ahead of the second week)|(death at 1 week)|(death at 168 hours)|(death at 168 hrs)|(death at 168hrs)|(death at one week)|(death at the 168th hour)|(death at the 168th hr)|(death at the 168thhr )|(death at the 1st week)|(death at the eighth day)|(death at the first week)|(death before 8 days)|(1-week death)|(1-week deceased)|(1-week died)|(1-week dying)|(1-week acute-fatality)|(1-week mortality)|(1wk death)|(1-wk death)|(1wk deceased)|(1-wk deceased)|(1wk died)|(1-wk died)|(1wk dying)|(1-wk dying)|(1wk acute-fatality)|(1-wk acute-fatality)|(1wk mortality)|(1-wk mortality)|(7 days death)|(7 days deceased)|(7 days died)|(7 days dying)|(7 days acute-fatality)|(7 days mortality)|(7days death)|(7days deceased)|(7days died)|(7days dying)|(7days acute-fatality)|(7days mortality)|(7th day death)|(7th day deceased)|(7th day died)|(7th day dying)|(7th day acute-fatality)|(7th day mortality)|(death ahead of the 2nd week)|(death ahead of the second week)|(death at 1 week)|(death at 168 hours)|(death at 168 hrs)|(death at 168hrs)|(death at one week)|(death at the 168th hour)|(death at the 168th hr)|(death at the 168thhr )|(death at the 1st week)|(death at the eighth day)|(death at the first week)|(death before 8 days)|(dying within the first 168 hrs)|(dying within the first 168hrs)|(dying within the first 7 days)|(dying within the first seven days)|(dying within the first week)|(dying within zero to 7 days)|(dying within zero to seven days)|(case fatality ahead of the 2nd week)|(case fatality ahead of the second week)|(case fatality at 1 week)|(case fatality at 168 hours)|(case fatality at 168 hrs)|(case fatality at 168hrs)|(case fatality at one week)|(case fatality at the 168th hour)|(case fatality at the 168th hr)|(case fatality at the 168thhr )|(case fatality at the 1st week)|(case fatality at the eighth day)|(case fatality at the first week)|(case fatality before 8 days)|(case fatality before the 2nd week)|(case fatality before the 8th day)|(case fatality before the eighth day)|(case fatality before the second week)|(case fatality by 1 week)|(case fatality by one week)|(case fatality by the 2nd week)|(case fatality by the end of the 1st week)|(case fatality by the end of the first week)|(case fatality by the second week)|(case fatality during 0 to 7 days)|(case fatality during 0 to seven days )|(case fatality during 1 week)|(case fatality during 1 week )|(case fatality during 168 hrs)|(case fatality during 168hrs)|(case fatality during 1st 7 days)|(case fatality during advance of the 2nd week)|(case fatality during advance of the second week)|(case fatality during one hundred and sixty eight hours)|(case fatality during one week)|(case fatality during one-week)|(dying within the first 168 hrs)|(dying within the first 168hrs)|(dying within the first 7 days)|(dying within the first seven days)|(dying within the first week)|(dying within zero to 7 days)|(dying within zero to seven days)|(case-fatality ahead of the 2nd week)|(case-fatality ahead of the second week)|(case-fatality at 1 week)|(case-fatality at 168 hours)|(case-fatality at 168 hrs)|(case-fatality at 168hrs)|(case-fatality at one week)|(case-fatality at the 168th hour)|(case-fatality at the 168th hr)|(case-fatality at the 168thhr )|(case-fatality at the 1st week)|(case-fatality at the eighth day)|(case-fatality at the first week)|(case-fatality before 8 days)|(case-fatality before the 2nd week)|(case-fatality before the 8th day)|(case-fatality before the eighth day)|(case-fatality before the second week)|(case-fatality by 1 week)|(case-fatality by one week)|(case-fatality by the 2nd week)|(case-fatality by the end of the 1st week)|(case-fatality by the end of the first week)|(case-fatality by the second week)|(case-fatality during 0 to 7 days)|(case-fatality during 0 to seven days )|(case-fatality during 1 week)|(case-fatality during 1 week )|(case-fatality during 168 hrs)|(case-fatality during 168hrs)|(case-fatality during 1st 7 days)|(case-fatality during advance of the 2nd week)|(case-fatality during advance of the second week)|(case-fatality during one hundred and sixty eight hours)|(case-fatality during one week)|(case-fatality during one-week)|(dying within the first 168 hrs)|(dying within the first 168hrs)|(dying within the first 7 days)|(dying within the first seven days)|(dying within the first week)|(dying within zero to 7 days)|(dying within zero to seven days)|(acute fatality ahead of the 2nd week)|(acute fatality ahead of the second week)|(acute fatality at 1 week)|(acute fatality at 168 hours)|(acute fatality at 168 hrs)|(acute fatality at 168hrs)|(acute fatality at one week)|(acute fatality at the 168th hour)|(acute fatality at the 168th hr)|(acute fatality at the 168thhr )|(acute fatality at the 1st week)|(acute fatality at the eighth day)|(acute fatality at the first week)|(acute fatality before 8 days)|(acute fatality before the 2nd week)|(acute fatality before the 8th day)|(acute fatality before the eighth day)|(acute fatality before the second week)|(acute fatality by 1 week)|(acute fatality by one week)|(acute fatality by the 2nd week)|(acute fatality by the end of the 1st week)|(acute fatality by the end of the first week)|(acute fatality by the second week)|(acute fatality during 0 to 7 days)|(acute fatality during 0 to seven days )|(acute fatality during 1 week)|(acute fatality during 1 week )|(acute fatality during 168 hrs)|(acute fatality during 168hrs)|(acute fatality during 1st 7 days)|(acute fatality during advance of the 2nd week)|(acute fatality during advance of the second week)|(acute fatality during one hundred and sixty eight hours)|(acute fatality during one week)|(acute fatality during one-week)|(dying within the first 168 hrs)|(dying within the first 168hrs)|(dying within the first 7 days)|(dying within the first seven days)|(dying within the first week)|(dying within zero to 7 days)|(dying within zero to seven days)|(acute-fatality ahead of the 2nd week)|(acute-fatality ahead of the second week)|(acute-fatality at 1 week)|(acute-fatality at 168 hours)|(acute-fatality at 168 hrs)|(acute-fatality at 168hrs)|(acute-fatality at one week)|(acute-fatality at the 168th hour)|(acute-fatality at the 168th hr)|(acute-fatality at the 168thhr )|(acute-fatality at the 1st week)|(acute-fatality at the eighth day)|(acute-fatality at the first week)|(acute-fatality before 8 days)|(acute-fatality before the 2nd week)|(acute-fatality before the 8th day)|(acute-fatality before the eighth day)|(acute-fatality before the second week)|(acute-fatality by 1 week)|(acute-fatality by one week)|(acute-fatality by the 2nd week)|(acute-fatality by the end of the 1st week)|(acute-fatality by the end of the first week)|(acute-fatality by the second week)|(acute-fatality during 0 to 7 days)|(acute-fatality during 0 to seven days )|(acute-fatality during 1 week)|(acute-fatality during 1 week )|(acute-fatality during 168 hrs)|(acute-fatality during 168hrs)|(acute-fatality during 1st 7 days)|(acute-fatality during advance of the 2nd week)|(acute-fatality during advance of the second week)|(acute-fatality during one hundred and sixty eight hours)|(acute-fatality during one week)|(acute-fatality during one-week)|(case fatality during one-wk)|(case fatality during seven days)|(case fatality during the 1st 168 hours)|(case fatality during the 1st 168 hrs)|(case fatality during the 1st 168hrs)|(case fatality during the 1st seven days )|(case fatality during the 1st week)|(case fatality during the first 168 hours)|(case fatality during the first 168 hrs)|(case fatality during the first 168hrs)|(case fatality during the first 7 days)|(case fatality during the first seven days)|(case fatality during the first week)|(case fatality during zero to 7 days)|(case fatality during zero to seven days)|(case fatality earlier than the 2nd week)|(case fatality earlier than the second week)|(case fatality equal to 7 days)|(case fatality in 0 to 7 days)|(case fatality in 0 to seven days )|(case fatality in 1 week)|(case fatality in 1 week )|(case fatality in 168 hrs)|(case fatality in 168hrs)|(case fatality in 1st 7 days)|(case fatality in advance of the 2nd week)|(case fatality in advance of the second week)|(case fatality in one hundred and sixty eight hours)|(case fatality in one week)|(case fatality in one-week)|(case fatality in one-wk)|(case fatality in seven days)|(case fatality in the 1st 168 hours)|(case fatality in the 1st 168 hrs)|(case fatality in the 1st 168hrs)|(case fatality in the 1st seven days )|(case fatality in the 1st week)|(case fatality in the first 168 hours)|(case-fatality during one-wk)|(case-fatality during seven days)|(case-fatality during the 1st 168 hours)|(case-fatality during the 1st 168 hrs)|(case-fatality during the 1st 168hrs)|(case-fatality during the 1st seven days )|(case-fatality during the 1st week)|(case-fatality during the first 168 hours)|(case-fatality during the first 168 hrs)|(case-fatality during the first 168hrs)|(case-fatality during the first 7 days)|(case-fatality during the first seven days)|(case-fatality during the first week)|(case-fatality during zero to 7 days)|(case-fatality during zero to seven days)|(case-fatality earlier than the 2nd week)|(case-fatality earlier than the second week)|(case-fatality equal to 7 days)|(case-fatality in 0 to 7 days)|(case-fatality in 0 to seven days )|(case-fatality in 1 week)|(case-fatality in 1 week )|(case-fatality in 168 hrs)|(case-fatality in 168hrs)|(case-fatality in 1st 7 days)|(case-fatality in advance of the 2nd week)|(case-fatality in advance of the second week)|(case-fatality in one hundred and sixty eight hours)|(case-fatality in one week)|(case-fatality in one-week)|(case-fatality in one-wk)|(case-fatality in seven days)|(case-fatality in the 1st 168 hours)|(case-fatality in the 1st 168 hrs)|(case-fatality in the 1st 168hrs)|(case-fatality in the 1st seven days )|(case-fatality in the 1st week)|(case-fatality in the first 168 hours)|(acute fatality during one-wk)|(acute fatality during seven days)|(acute fatality during the 1st 168 hours)|(acute fatality during the 1st 168 hrs)|(acute fatality during the 1st 168hrs)|(acute fatality during the 1st seven days )|(acute fatality during the 1st week)|(acute fatality during the first 168 hours)|(acute fatality during the first 168 hrs)|(acute fatality during the first 168hrs)|(acute fatality during the first 7 days)|(acute fatality during the first seven days)|(acute fatality during the first week)|(acute fatality during zero to 7 days)|(acute fatality during zero to seven days)|(acute fatality earlier than the 2nd week)|(acute fatality earlier than the second week)|(acute fatality equal to 7 days)|(acute fatality in 0 to 7 days)|(acute fatality in 0 to seven days )|(acute fatality in 1 week)|(acute fatality in 1 week )|(acute fatality in 168 hrs)|(acute fatality in 168hrs)|(acute fatality in 1st 7 days)|(acute fatality in advance of the 2nd week)|(acute fatality in advance of the second week)|(acute fatality in one hundred and sixty eight hours)|(acute fatality in one week)|(acute fatality in one-week)|(acute fatality in one-wk)|(acute fatality in seven days)|(acute fatality in the 1st 168 hours)|(acute fatality in the 1st 168 hrs)|(acute fatality in the 1st 168hrs)|(acute fatality in the 1st seven days )|(acute fatality in the 1st week)|(acute fatality in the first 168 hours)|(acute-fatality during one-wk)|(acute-fatality during seven days)|(acute-fatality during the 1st 168 hours)|(acute-fatality during the 1st 168 hrs)|(acute-fatality during the 1st 168hrs)|(acute-fatality during the 1st seven days )|(acute-fatality during the 1st week)|(acute-fatality during the first 168 hours)|(acute-fatality during the first 168 hrs)|(acute-fatality during the first 168hrs)|(acute-fatality during the first 7 days)|(acute-fatality during the first seven days)|(acute-fatality during the first week)|(acute-fatality during zero to 7 days)|(acute-fatality during zero to seven days)|(acute-fatality earlier than the 2nd week)|(acute-fatality earlier than the second week)|(acute-fatality equal to 7 days)|(acute-fatality in 0 to 7 days)|(acute-fatality in 0 to seven days )|(acute-fatality in 1 week)|(acute-fatality in 1 week )|(acute-fatality in 168 hrs)|(acute-fatality in 168hrs)|(acute-fatality in 1st 7 days)|(acute-fatality in advance of the 2nd week)|(acute-fatality in advance of the second week)|(acute-fatality in one hundred and sixty eight hours)|(acute-fatality in one week)|(acute-fatality in one-week)|(acute-fatality in one-wk)|(acute-fatality in seven days)|(acute-fatality in the 1st 168 hours)|(acute-fatality in the 1st 168 hrs)|(acute-fatality in the 1st 168hrs)|(acute-fatality in the 1st seven days )|(acute-fatality in the 1st week)|(acute-fatality in the first 168 hours)|(case fatality in the first 168 hrs)|(case fatality in the first 168hrs)|(case fatality in the first 7 days)|(case fatality in the first seven days)|(case fatality in the first week)|(case fatality in zero to 7 days)|(case fatality in zero to seven days)|(case fatality less than 1 week)|(case fatality less than 169 hours)|(case fatality less than 8 days)|(case fatality less than one week)|(case fatality less than or equal to 7 days)|(case fatality less than or equal to seven days)|(case fatality less than the 1st week)|(case fatality less than the first week)|(case fatality on the 7th day)|(case fatality on the seventh day)|(case fatality over 1 week)|(case fatality over one week)|(case fatality previous to the 2nd week)|(case fatality previous to the second week)|(case fatality prior to the 2nd week)|(case fatality prior to the second week)|(case fatality sooner than the 2nd week)|(case fatality sooner than the second week)|(case fatality up to 7 days)|(case fatality up to day 7)|(case fatality up to day seven)|(case fatality up to seven days)|(case fatality up to the 7th day)|(case fatality up to the seventh day)|(case fatality within 0 to 7 days)|(case-fatality in the first 168 hrs)|(case-fatality in the first 168hrs)|(case-fatality in the first 7 days)|(case-fatality in the first seven days)|(case-fatality in the first week)|(case-fatality in zero to 7 days)|(case-fatality in zero to seven days)|(case-fatality less than 1 week)|(case-fatality less than 169 hours)|(case-fatality less than 8 days)|(case-fatality less than one week)|(case-fatality less than or equal to 7 days)|(case-fatality less than or equal to seven days)|(case-fatality less than the 1st week)|(case-fatality less than the first week)|(case-fatality on the 7th day)|(case-fatality on the seventh day)|(case-fatality over 1 week)|(case-fatality over one week)|(case-fatality previous to the 2nd week)|(case-fatality previous to the second week)|(case-fatality prior to the 2nd week)|(case-fatality prior to the second week)|(case-fatality sooner than the 2nd week)|(case-fatality sooner than the second week)|(case-fatality up to 7 days)|(case-fatality up to day 7)|(case-fatality up to day seven)|(case-fatality up to seven days)|(case-fatality up to the 7th day)|(case-fatality up to the seventh day)|(case-fatality within 0 to 7 days)|(acute fatality in the first 168 hrs)|(acute fatality in the first 168hrs)|(acute fatality in the first 7 days)|(acute fatality in the first seven days)|(acute fatality in the first week)|(acute fatality in zero to 7 days)|(acute fatality in zero to seven days)|(acute fatality less than 1 week)|(acute fatality less than 169 hours)|(acute fatality less than 8 days)|(acute fatality less than one week)|(acute fatality less than or equal to 7 days)|(acute fatality less than or equal to seven days)|(acute fatality less than the 1st week)|(acute fatality less than the first week)|(acute fatality on the 7th day)|(acute fatality on the seventh day)|(acute fatality over 1 week)|(acute fatality over one week)|(acute fatality previous to the 2nd week)|(acute fatality previous to the second week)|(acute fatality prior to the 2nd week)|(acute fatality prior to the second week)|(acute fatality sooner than the 2nd week)|(acute fatality sooner than the second week)|(acute fatality up to 7 days)|(acute fatality up to day 7)|(acute fatality up to day seven)|(acute fatality up to seven days)|(acute fatality up to the 7th day)|(acute fatality up to the seventh day)|(acute fatality within 0 to 7 days)|(acute-fatality in the first 168 hrs)|(acute-fatality in the first 168hrs)|(acute-fatality in the first 7 days)|(acute-fatality in the first seven days)|(acute-fatality in the first week)|(acute-fatality in zero to 7 days)|(acute-fatality in zero to seven days)|(acute-fatality less than 1 week)|(acute-fatality less than 169 hours)|(acute-fatality less than 8 days)|(acute-fatality less than one week)|(acute-fatality less than or equal to 7 days)|(acute-fatality less than or equal to seven days)|(acute-fatality less than the 1st week)|(acute-fatality less than the first week)|(acute-fatality on the 7th day)|(acute-fatality on the seventh day)|(acute-fatality over 1 week)|(acute-fatality over one week)|(acute-fatality previous to the 2nd week)|(acute-fatality previous to the second week)|(acute-fatality prior to the 2nd week)|(acute-fatality prior to the second week)|(acute-fatality sooner than the 2nd week)|(acute-fatality sooner than the second week)|(acute-fatality up to 7 days)|(acute-fatality up to day 7)|(acute-fatality up to day seven)|(acute-fatality up to seven days)|(acute-fatality up to the 7th day)|(acute-fatality up to the seventh day)|(acute-fatality within 0 to 7 days)|(case fatality within 0 to seven days )|(case fatality within 1 week)|(case fatality within 1 week)|(case fatality within 168 hours)|(case fatality within 168 hrs)|(case fatality within 168hrs)|(case fatality within 1st 7 days)|(case fatality within 7 days)|(case fatality within a week)|(case fatality within one hundred and sixty eight hours)|(case fatality within one week)|(case fatality within one-week)|(case fatality within one-wk)|(case fatality within seven days)|(case fatality within the 1st 168 hours)|(case fatality within the 1st 168 hrs)|(case fatality within the 1st 168hrs)|(case fatality within the 1st seven days)|(case fatality within the 1st week)|(case fatality within the first 168 hours)|(case fatality within the first 168 hrs)|(case fatality within the first 168hrs)|(case fatality within the first 7 days)|(case fatality within the first seven days)|(case fatality within the first week)|(case fatality within zero to 7 days)|(case fatality within zero to seven days)|(mortality ahead of the 2nd week)|(mortality ahead of the second wee)|(mortality at 1 week)|(mortality at 168 hours)|(mortality at 168 hrs)|(mortality at 168hrs)|(mortality at one week)|(case-fatality within 0 to seven days )|(case-fatality within 1 week)|(case-fatality within 1 week)|(case-fatality within 168 hours)|(case-fatality within 168 hrs)|(case-fatality within 168hrs)|(case-fatality within 1st 7 days)|(case-fatality within 7 days)|(case-fatality within a week)|(case-fatality within one hundred and sixty eight hours)|(case-fatality within one week)|(case-fatality within one-week)|(case-fatality within one-wk)|(case-fatality within seven days)|(case-fatality within the 1st 168 hours)|(case-fatality within the 1st 168 hrs)|(case-fatality within the 1st 168hrs)|(case-fatality within the 1st seven days)|(case-fatality within the 1st week)|(case-fatality within the first 168 hours)|(case-fatality within the first 168 hrs)|(case-fatality within the first 168hrs)|(case-fatality within the first 7 days)|(case-fatality within the first seven days)|(case-fatality within the first week)|(case-fatality within zero to 7 days)|(case-fatality within zero to seven days)|(mortality ahead of the 2nd week)|(mortality ahead of the second wee)|(mortality at 1 week)|(mortality at 168 hours)|(mortality at 168 hrs)|(mortality at 168hrs)|(mortality at one week)|(acute fatality within 0 to seven days )|(acute fatality within 1 week)|(acute fatality within 1 week)|(acute fatality within 168 hours)|(acute fatality within 168 hrs)|(acute fatality within 168hrs)|(acute fatality within 1st 7 days)|(acute fatality within 7 days)|(acute fatality within a week)|(acute fatality within one hundred and sixty eight hours)|(acute fatality within one week)|(acute fatality within one-week)|(acute fatality within one-wk)|(acute fatality within seven days)|(acute fatality within the 1st 168 hours)|(acute fatality within the 1st 168 hrs)|(acute fatality within the 1st 168hrs)|(acute fatality within the 1st seven days)|(acute fatality within the 1st week)|(acute fatality within the first 168 hours)|(acute fatality within the first 168 hrs)|(acute fatality within the first 168hrs)|(acute fatality within the first 7 days)|(acute fatality within the first seven days)|(acute fatality within the first week)|(acute fatality within zero to 7 days)|(acute fatality within zero to seven days)|(mortality ahead of the 2nd week)|(mortality ahead of the second wee)|(mortality at 1 week)|(mortality at 168 hours)|(mortality at 168 hrs)|(mortality at 168hrs)|(mortality at one week)|(acute-fatality within 0 to seven days )|(acute-fatality within 1 week)|(acute-fatality within 1 week)|(acute-fatality within 168 hours)|(acute-fatality within 168 hrs)|(acute-fatality within 168hrs)|(acute-fatality within 1st 7 days)|(acute-fatality within 7 days)|(acute-fatality within a week)|(acute-fatality within one hundred and sixty eight hours)|(acute-fatality within one week)|(acute-fatality within one-week)|(acute-fatality within one-wk)|(acute-fatality within seven days)|(acute-fatality within the 1st 168 hours)|(acute-fatality within the 1st 168 hrs)|(acute-fatality within the 1st 168hrs)|(acute-fatality within the 1st seven days)|(acute-fatality within the 1st week)|(acute-fatality within the first 168 hours)|(acute-fatality within the first 168 hrs)|(acute-fatality within the first 168hrs)|(acute-fatality within the first 7 days)|(acute-fatality within the first seven days)|(acute-fatality within the first week)|(acute-fatality within zero to 7 days)|(acute-fatality within zero to seven days)|(mortality ahead of the 2nd week)|(mortality ahead of the second wee)|(mortality at 1 week)|(mortality at 168 hours)|(mortality at 168 hrs)|(mortality at 168hrs)|(mortality at one week)|(mortality in one hundred and forty four hours)|(mortality in one hundred and forty-four hours)|(mortality at one hundred and forty four hours)|(mortality at one hundred and forty-four hours)|(168 hour mortality)|(168-hour mortality)|(168-hr mortality)|(168hr mortality)|(168 hr mortality)|(one hundred and sixty eight hour mortality)|(one hundred and sixty-eight hour mortality)|(mortality at seven days)|(mortality at 7 days)|(mortality in seven days)|(mortality in 7 days)|(mortality at 168 hours)|(mortality in 168 hours)|(mortality in one hundred and sixty eight hours)|(mortality in one hundred and sixty-eight hours)|(mortality at one hundred and sixty eight hours)|(mortality at one hundred and sixty-eight hours)|(case fatality sooner than the first week)|(case fatality sooner than the 1^st^ week)|(case fatality sooner than the 1^st^ wk)|(mortality in one hundred and forty four hours)|(mortality in one hundred and forty-four hours)|(mortality at one hundred and forty four hours)|(mortality at one hundred and forty-four hours)|(168 hour mortality)|(168-hour mortality)|(168-hr mortality)|(168hr mortality)|(168 hr mortality)|(one hundred and sixty eight hour mortality)|(one hundred and sixty-eight hour mortality)|(mortality at seven days)|(mortality at 7 days)|(mortality in seven days)|(mortality in 7 days)|(mortality at 168 hours)|(mortality in 168 hours)|(mortality in one hundred and sixty eight hours)|(mortality in one hundred and sixty-eight hours)|(mortality at one hundred and sixty eight hours)|(mortality at one hundred and sixty-eight hours)|(case-fatality sooner than the first week)|(case-fatality sooner than the 1^st^ week)|(case-fatality sooner than the 1^st^ wk)|(mortality in one hundred and forty four hours)|(mortality in one hundred and forty-four hours)|(mortality at one hundred and forty four hours)|(mortality at one hundred and forty-four hours)|(168 hour mortality)|(168-hour mortality)|(168-hr mortality)|(168hr mortality)|(168 hr mortality)|(one hundred and sixty eight hour mortality)|(one hundred and sixty-eight hour mortality)|(mortality at seven days)|(mortality at 7 days)|(mortality in seven days)|(mortality in 7 days)|(mortality at 168 hours)|(mortality in 168 hours)|(mortality in one hundred and sixty eight hours)|(mortality in one hundred and sixty-eight hours)|(mortality at one hundred and sixty eight hours)|(mortality at one hundred and sixty-eight hours)|(acute fatality sooner than the first week)|(acute fatality sooner than the 1^st^ week)|(acute fatality sooner than the 1^st^ wk)|(mortality in one hundred and forty four hours)|(mortality in one hundred and forty-four hours)|(mortality at one hundred and forty four hours)|(mortality at one hundred and forty-four hours)|(168 hour mortality)|(168-hour mortality)|(168-hr mortality)|(168hr mortality)|(168 hr mortality)|(one hundred and sixty eight hour mortality)|(one hundred and sixty-eight hour mortality)|(mortality at seven days)|(mortality at 7 days)|(mortality in seven days)|(mortality in 7 days)|(mortality at 168 hours)|(mortality in 168 hours)|(mortality in one hundred and sixty eight hours)|(mortality in one hundred and sixty-eight hours)|(mortality at one hundred and sixty eight hours)|(mortality at one hundred and sixty-eight hours)|(acute-fatality sooner than the first week)|(acute-fatality sooner than the 1^st^ week)|(acute-fatality sooner than the 1^st^ wk)|(case fatality sooner than 7 days)|(case fatality sooner than seven days)|(case fatality sooner than the 7^th^ day)|(case fatality sooner than the seventh day)|(24 hour case fatality)|(24-hour case fatality)|(24-hr case fatality)|(24hr case fatality)|(24 hr case fatality)|(twenty four hour case fatality)|(twenty-four hour case fatality)|(case fatality at one day)|(case fatality at 1 day)|(case fatality in one day)|(case fatality in 1 day)|(case fatality at 24 hours)|(case fatality in 24 hours)|(case fatality in twenty four hours)|(case fatality in twenty-four hours)|(case fatality at twenty-four hours)|(case fatality at twenty four hours)|(48 hour case fatality)|(48-hour case fatality)|(48-hr case fatality)|(48hr case fatality)|(48 hr case fatality)|(forty eight hour case fatality)|(forty-eight hour case fatality)|(case fatality at two days)|(case fatality at 2 days)|(case fatality in two days)|(case fatality in 2 days)|(case fatality at 48 hours)|(case fatality in 48 hours)|(case fatality in forty eight hours)|(case fatality in forty-eight hours)|(case fatality at forty eight hours)|(case fatality at forty-eight hours)|(72 hour case fatality)|(72-hour case fatality)|(72-hr case fatality)|(72hr case fatality)|(72 hr case fatality)|(seventy two hour case fatality)|(seventy-two hour case fatality)|(case fatality at three days)|(case fatality at 3 days)|(case fatality in three days)|(case-fatality sooner than 7 days)|(case-fatality sooner than seven days)|(case-fatality sooner than the 7^th^ day)|(case-fatality sooner than the seventh day)|(24 hour case-fatality)|(24-hour case-fatality)|(24-hr case-fatality)|(24hr case-fatality)|(24 hr case-fatality)|(twenty four hour case-fatality)|(twenty-four hour case-fatality)|(case-fatality at one day)|(case-fatality at 1 day)|(case-fatality in one day)|(case-fatality in 1 day)|(case-fatality at 24 hours)|(case-fatality in 24 hours)|(case-fatality in twenty four hours)|(case-fatality in twenty-four hours)|(case-fatality at twenty-four hours)|(case-fatality at twenty four hours)|(48 hour case-fatality)|(48-hour case-fatality)|(48-hr case-fatality)|(48hr case-fatality)|(48 hr case-fatality)|(forty eight hour case-fatality)|(forty-eight hour case-fatality)|(case-fatality at two days)|(case-fatality at 2 days)|(case-fatality in two days)|(case-fatality in 2 days)|(case-fatality at 48 hours)|(case-fatality in 48 hours)|(case-fatality in forty eight hours)|(case-fatality in forty-eight hours)|(case-fatality at forty eight hours)|(case-fatality at forty-eight hours)|(72 hour case-fatality)|(72-hour case-fatality)|(72-hr case-fatality)|(72hr case-fatality)|(72 hr case-fatality)|(seventy two hour case-fatality)|(seventy-two hour case-fatality)|(case-fatality at three days)|(case-fatality at 3 days)|(case-fatality in three days)|(acute fatality sooner than 7 days)|(acute fatality sooner than seven days)|(acute fatality sooner than the 7^th^ day)|(acute fatality sooner than the seventh day)|(24 hour acute fatality)|(24-hour acute fatality)|(24-hr acute fatality)|(24hr acute fatality)|(24 hr acute fatality)|(twenty four hour acute fatality)|(twenty-four hour acute fatality)|(acute fatality at one day)|(acute fatality at 1 day)|(acute fatality in one day)|(acute fatality in 1 day)|(acute fatality at 24 hours)|(acute fatality in 24 hours)|(acute fatality in twenty four hours)|(acute fatality in twenty-four hours)|(acute fatality at twenty-four hours)|(acute fatality at twenty four hours)|(48 hour acute fatality)|(48-hour acute fatality)|(48-hr acute fatality)|(48hr acute fatality)|(48 hr acute fatality)|(forty eight hour acute fatality)|(forty-eight hour acute fatality)|(acute fatality at two days)|(acute fatality at 2 days)|(acute fatality in two days)|(acute fatality in 2 days)|(acute fatality at 48 hours)|(acute fatality in 48 hours)|(acute fatality in forty eight hours)|(acute fatality in forty-eight hours)|(acute fatality at forty eight hours)|(acute fatality at forty-eight hours)|(72 hour acute fatality)|(72-hour acute fatality)|(72-hr acute fatality)|(72hr acute fatality)|(72 hr acute fatality)|(seventy two hour acute fatality)|(seventy-two hour acute fatality)|(acute fatality at three days)|(acute fatality at 3 days)|(acute fatality in three days)|(acute-fatality sooner than 7 days)|(acute-fatality sooner than seven days)|(acute-fatality sooner than the 7^th^ day)|(acute-fatality sooner than the seventh day)|(24 hour acute-fatality)|(24-hour acute-fatality)|(24-hr acute-fatality)|(24hr acute-fatality)|(24 hr acute-fatality)|(twenty four hour acute-fatality)|(twenty-four hour acute-fatality)|(acute-fatality at one day)|(acute-fatality at 1 day)|(acute-fatality in one day)|(acute-fatality in 1 day)|(acute-fatality at 24 hours)|(acute-fatality in 24 hours)|(acute-fatality in twenty four hours)|(acute-fatality in twenty-four hours)|(acute-fatality at twenty-four hours)|(acute-fatality at twenty four hours)|(48 hour acute-fatality)|(48-hour acute-fatality)|(48-hr acute-fatality)|(48hr acute-fatality)|(48 hr acute-fatality)|(forty eight hour acute-fatality)|(forty-eight hour acute-fatality)|(acute-fatality at two days)|(acute-fatality at 2 days)|(acute-fatality in two days)|(acute-fatality in 2 days)|(acute-fatality at 48 hours)|(acute-fatality in 48 hours)|(acute-fatality in forty eight hours)|(acute-fatality in forty-eight hours)|(acute-fatality at forty eight hours)|(acute-fatality at forty-eight hours)|(72 hour acute-fatality)|(72-hour acute-fatality)|(72-hr acute-fatality)|(72hr acute-fatality)|(72 hr acute-fatality)|(seventy two hour acute-fatality)|(seventy-two hour acute-fatality)|(acute-fatality at three days)|(acute-fatality at 3 days)|(acute-fatality in three days)|(case fatality in 3 days)|(case fatality at 72 hours)|(case fatality in 72 hours)|(case fatality in seventy two hours)|(case fatality in seventy-two hours)|(case fatality at seventy two hours)|(case fatality at seventy-two hours)|(96 hour case fatality)|(96-hour case fatality)|(96-hr case fatality)|(96hr case fatality)|(96 hr case fatality)|(ninety six hour case fatality)|(ninety-six hour case fatality)|(case fatality at 4 days)|(case fatality at 4 days)|(case fatality in four days)|(case fatality in 4 days)|(case fatality at 96 hours)|(case fatality in 96 hours)|(case fatality in ninety six hours)|(case fatality in ninety-six hours)|(case fatality at ninety six hours)|(case fatality at ninety-six hours)|(120 hour case fatality)|(120-hour case fatality)|(120-hr case fatality)|(120hr case fatality)|(120 hr case fatality)|(one hundred and twenty hour case fatality)|(case fatality at five days)|(case fatality at 5 days)|(case fatality in five days)|(case fatality in 5 days)|(case fatality at one hundred and twenty hours)|(case fatality in one hundred and twenty hours)|(case fatality at one hundred and twenty hours)|(144 hour case fatality)|(case-fatality in 3 days)|(case-fatality at 72 hours)|(case-fatality in 72 hours)|(case-fatality in seventy two hours)|(case-fatality in seventy-two hours)|(case-fatality at seventy two hours)|(case-fatality at seventy-two hours)|(96 hour case-fatality)|(96-hour case-fatality)|(96-hr case-fatality)|(96hr case-fatality)|(96 hr case-fatality)|(ninety six hour case-fatality)|(ninety-six hour case-fatality)|(case-fatality at 4 days)|(case-fatality at 4 days)|(case-fatality in four days)|(case-fatality in 4 days)|(case-fatality at 96 hours)|(case-fatality in 96 hours)|(case-fatality in ninety six hours)|(case-fatality in ninety-six hours)|(case-fatality at ninety six hours)|(case-fatality at ninety-six hours)|(120 hour case-fatality)|(120-hour case-fatality)|(120-hr case-fatality)|(120hr case-fatality)|(120 hr case-fatality)|(one hundred and twenty hour case-fatality)|(case-fatality at five days)|(case-fatality at 5 days)|(case-fatality in five days)|(case-fatality in 5 days)|(case-fatality at one hundred and twenty hours)|(case-fatality in one hundred and twenty hours)|(case-fatality at one hundred and twenty hours)|(144 hour case-fatality)|(acute fatality in 3 days)|(acute fatality at 72 hours)|(acute fatality in 72 hours)|(acute fatality in seventy two hours)|(acute fatality in seventy-two hours)|(acute fatality at seventy two hours)|(acute fatality at seventy-two hours)|(96 hour acute fatality)|(96-hour acute fatality)|(96-hr acute fatality)|(96hr acute fatality)|(96 hr acute fatality)|(ninety six hour acute fatality)|(ninety-six hour acute fatality)|(acute fatality at 4 days)|(acute fatality at 4 days)|(acute fatality in four days)|(acute fatality in 4 days)|(acute fatality at 96 hours)|(acute fatality in 96 hours)|(acute fatality in ninety six hours)|(acute fatality in ninety-six hours)|(acute fatality at ninety six hours)|(acute fatality at ninety-six hours)|(120 hour acute fatality)|(120-hour acute fatality)|(120-hr acute fatality)|(120hr acute fatality)|(120 hr acute fatality)|(one hundred and twenty hour acute fatality)|(acute fatality at five days)|(acute fatality at 5 days)|(acute fatality in five days)|(acute fatality in 5 days)|(acute fatality at one hundred and twenty hours)|(acute fatality in one hundred and twenty hours)|(acute fatality at one hundred and twenty hours)|(144 hour acute fatality)|(acute-fatality in 3 days)|(acute-fatality at 72 hours)|(acute-fatality in 72 hours)|(acute-fatality in seventy two hours)|(acute-fatality in seventy-two hours)|(acute-fatality at seventy two hours)|(acute-fatality at seventy-two hours)|(96 hour acute-fatality)|(96-hour acute-fatality)|(96-hr acute-fatality)|(96hr acute-fatality)|(96 hr acute-fatality)|(ninety six hour acute-fatality)|(ninety-six hour acute-fatality)|(acute-fatality at 4 days)|(acute-fatality at 4 days)|(acute-fatality in four days)|(acute-fatality in 4 days)|(acute-fatality at 96 hours)|(acute-fatality in 96 hours)|(acute-fatality in ninety six hours)|(acute-fatality in ninety-six hours)|(acute-fatality at ninety six hours)|(acute-fatality at ninety-six hours)|(120 hour acute-fatality)|(120-hour acute-fatality)|(120-hr acute-fatality)|(120hr acute-fatality)|(120 hr acute-fatality)|(one hundred and twenty hour acute-fatality)|(acute-fatality at five days)|(acute-fatality at 5 days)|(acute-fatality in five days)|(acute-fatality in 5 days)|(acute-fatality at one hundred and twenty hours)|(acute-fatality in one hundred and twenty hours)|(acute-fatality at one hundred and twenty hours)|(144 hour acute-fatality)|(144-hour case fatality)|(144-hr case fatality)|(144hr case fatality)|(144 hr case fatality)|(one hundred and forty four hour case fatality)|(one hundred and forty-four hour case fatality)|(case fatality at six days)|(case fatality at 6 days)|(case fatality in six days)|(case fatality in 6 days)|(case fatality at 144 hours)|(case fatality in 144 hours)|(case fatality in one hundred and forty four hours)|(case fatality in one hundred and forty-four hours)|(case fatality at one hundred and forty four hours)|(case fatality at one hundred and forty-four hours)|(168 hour case fatality)|(168-hour case fatality)|(168-hr case fatality)|(168hr case fatality)|(168 hr case fatality)|(one hundred and sixty eight hour case fatality)|(one hundred and sixty-eight hour case fatality)|(case fatality at seven days)|(case fatality at 7 days)|(case fatality in seven days)|(case fatality in 7 days)|(case fatality at 168 hours)|(case fatality in 168 hours)|(144-hour case-fatality)|(144-hr case-fatality)|(144hr case-fatality)|(144 hr case-fatality)|(one hundred and forty four hour case-fatality)|(one hundred and forty-four hour case-fatality)|(case-fatality at six days)|(case-fatality at 6 days)|(case-fatality in six days)|(case-fatality in 6 days)|(case-fatality at 144 hours)|(case-fatality in 144 hours)|(case-fatality in one hundred and forty four hours)|(case-fatality in one hundred and forty-four hours)|(case-fatality at one hundred and forty four hours)|(case-fatality at one hundred and forty-four hours)|(168 hour case-fatality)|(168-hour case-fatality)|(168-hr case-fatality)|(168hr case-fatality)|(168 hr case-fatality)|(one hundred and sixty eight hour case-fatality)|(one hundred and sixty-eight hour case-fatality)|(case-fatality at seven days)|(case-fatality at 7 days)|(case-fatality in seven days)|(case-fatality in 7 days)|(case-fatality at 168 hours)|(case-fatality in 168 hours)|(144-hour acute fatality)|(144-hr acute fatality)|(144hr acute fatality)|(144 hr acute fatality)|(one hundred and forty four hour acute fatality)|(one hundred and forty-four hour acute fatality)|(acute fatality at six days)|(acute fatality at 6 days)|(acute fatality in six days)|(acute fatality in 6 days)|(acute fatality at 144 hours)|(acute fatality in 144 hours)|(acute fatality in one hundred and forty four hours)|(acute fatality in one hundred and forty-four hours)|(acute fatality at one hundred and forty four hours)|(acute fatality at one hundred and forty-four hours)|(168 hour acute fatality)|(168-hour acute fatality)|(168-hr acute fatality)|(168hr acute fatality)|(168 hr acute fatality)|(one hundred and sixty eight hour acute fatality)|(one hundred and sixty-eight hour acute fatality)|(acute fatality at seven days)|(acute fatality at 7 days)|(acute fatality in seven days)|(acute fatality in 7 days)|(acute fatality at 168 hours)|(acute fatality in 168 hours)|(144-hour acute-fatality)|(144-hr acute-fatality)|(144hr acute-fatality)|(144 hr acute-fatality)|(one hundred and forty four hour acute-fatality)|(one hundred and forty-four hour acute-fatality)|(acute-fatality at six days)|(acute-fatality at 6 days)|(acute-fatality in six days)|(acute-fatality in 6 days)|(acute-fatality at 144 hours)|(acute-fatality in 144 hours)|(acute-fatality in one hundred and forty four hours)|(acute-fatality in one hundred and forty-four hours)|(acute-fatality at one hundred and forty four hours)|(acute-fatality at one hundred and forty-four hours)|(168 hour acute-fatality)|(168-hour acute-fatality)|(168-hr acute-fatality)|(168hr acute-fatality)|(168 hr acute-fatality)|(one hundred and sixty eight hour acute-fatality)|(one hundred and sixty-eight hour acute-fatality)|(acute-fatality at seven days)|(acute-fatality at 7 days)|(acute-fatality in seven days)|(acute-fatality in 7 days)|(acute-fatality at 168 hours)|(acute-fatality in 168 hours)|(case fatality in one hundred and sixty eight hours)|(case fatality in one hundred and sixty-eight hours)|(case fatality at one hundred and sixty eight hours)|(case fatality at one hundred and sixty-eight hours)|(death sooner than the first week)|(death sooner than the 1^st^ week)|(death sooner than the 1^st^ wk)|(death sooner than 7 days)|(death sooner than seven days)|(death sooner than the 7^th^ day)|(death sooner than the seventh day)|(24 hour death)|(24-hour death)|(24-hr death)|(24hr death)|(24 hr death)|(twenty four hour death)|(twenty-four hour death)|(death at one day)|(death at 1 day)|(death in one day)|(death in 1 day)|(death at 24 hours)|(death in 24 hours)|(death in twenty four hours)|(death in twenty-four hours)|(death at twenty-four hours)|(death at twenty four hours)|(48 hour death)|(48-hour death)|(48-hr death)|(48hr death)|(48 hr death)|(forty eight hour death)|(forty-eight hour death)|(death at two days)|(death at 2 days)|(death in two days)|(death in 2 days)|(death at 48 hours)|(case-fatality in one hundred and sixty eight hours)|(case-fatality in one hundred and sixty-eight hours)|(case-fatality at one hundred and sixty eight hours)|(case-fatality at one hundred and sixty-eight hours)|(death sooner than the first week)|(death sooner than the 1^st^ week)|(death sooner than the 1^st^ wk)|(death sooner than 7 days)|(death sooner than seven days)|(death sooner than the 7^th^ day)|(death sooner than the seventh day)|(24 hour death)|(24-hour death)|(24-hr death)|(24hr death)|(24 hr death)|(twenty four hour death)|(twenty-four hour death)|(death at one day)|(death at 1 day)|(death in one day)|(death in 1 day)|(death at 24 hours)|(death in 24 hours)|(death in twenty four hours)|(death in twenty-four hours)|(death at twenty-four hours)|(death at twenty four hours)|(48 hour death)|(48-hour death)|(48-hr death)|(48hr death)|(48 hr death)|(forty eight hour death)|(forty-eight hour death)|(death at two days)|(death at 2 days)|(death in two days)|(death in 2 days)|(death at 48 hours)|(acute fatality in one hundred and sixty eight hours)|(acute fatality in one hundred and sixty-eight hours)|(acute fatality at one hundred and sixty eight hours)|(acute fatality at one hundred and sixty-eight hours)|(death sooner than the first week)|(death sooner than the 1^st^ week)|(death sooner than the 1^st^ wk)|(death sooner than 7 days)|(death sooner than seven days)|(death sooner than the 7^th^ day)|(death sooner than the seventh day)|(24 hour death)|(24-hour death)|(24-hr death)|(24hr death)|(24 hr death)|(twenty four hour death)|(twenty-four hour death)|(death at one day)|(death at 1 day)|(death in one day)|(death in 1 day)|(death at 24 hours)|(death in 24 hours)|(death in twenty four hours)|(death in twenty-four hours)|(death at twenty-four hours)|(death at twenty four hours)|(48 hour death)|(48-hour death)|(48-hr death)|(48hr death)|(48 hr death)|(forty eight hour death)|(forty-eight hour death)|(death at two days)|(death at 2 days)|(death in two days)|(death in 2 days)|(death at 48 hours)|(acute-fatality in one hundred and sixty eight hours)|(acute-fatality in one hundred and sixty-eight hours)|(acute-fatality at one hundred and sixty eight hours)|(acute-fatality at one hundred and sixty-eight hours)|(death sooner than the first week)|(death sooner than the 1^st^ week)|(death sooner than the 1^st^ wk)|(death sooner than 7 days)|(death sooner than seven days)|(death sooner than the 7^th^ day)|(death sooner than the seventh day)|(24 hour death)|(24-hour death)|(24-hr death)|(24hr death)|(24 hr death)|(twenty four hour death)|(twenty-four hour death)|(death at one day)|(death at 1 day)|(death in one day)|(death in 1 day)|(death at 24 hours)|(death in 24 hours)|(death in twenty four hours)|(death in twenty-four hours)|(death at twenty-four hours)|(death at twenty four hours)|(48 hour death)|(48-hour death)|(48-hr death)|(48hr death)|(48 hr death)|(forty eight hour death)|(forty-eight hour death)|(death at two days)|(death at 2 days)|(death in two days)|(death in 2 days)|(death at 48 hours)|(1-week death)|(1-week deceased)|(1-week died)|(1-week dying)|(1-week fatality)|(1-week case mortality)|(1wk death)|(1-wk death)|(1wk deceased)|(1-wk deceased)|(1wk died)|(1-wk died)|(1wk dying)|(1-wk dying)|(1wk fatality)|(1-wk fatality)|(1wk case mortality)|(1-wk case mortality)|(7 days death)|(7 days deceased)|(7 days died)|(7 days dying)|(7 days fatality)|(7 days case mortality)|(7days death)|(7days deceased)|(7days died)|(7days dying)|(7days fatality)|(7days case mortality)|(7th day death)|(7th day deceased)|(7th day died)|(7th day dying)|(7th day fatality)|(7th day case mortality)|(death ahead of the 2nd week)|(death ahead of the second week)|(death at 1 week)|(death at 168 hours)|(death at 168 hrs)|(death at 168hrs)|(death at one week)|(death at the 168th hour)|(death at the 168th hr)|(death at the 168thhr )|(death at the 1st week)|(death at the eighth day)|(death at the first week)|(death before 8 days)|(1-week death)|(1-week deceased)|(1-week died)|(1-week dying)|(1-week fatality)|(1-week case-mortality)|(1wk death)|(1-wk death)|(1wk deceased)|(1-wk deceased)|(1wk died)|(1-wk died)|(1wk dying)|(1-wk dying)|(1wk fatality)|(1-wk fatality)|(1wk case-mortality)|(1-wk case-mortality)|(7 days death)|(7 days deceased)|(7 days died)|(7 days dying)|(7 days fatality)|(7 days case-mortality)|(7days death)|(7days deceased)|(7days died)|(7days dying)|(7days fatality)|(7days case-mortality)|(7th day death)|(7th day deceased)|(7th day died)|(7th day dying)|(7th day fatality)|(7th day case-mortality)|(death ahead of the 2nd week)|(death ahead of the second week)|(death at 1 week)|(death at 168 hours)|(death at 168 hrs)|(death at 168hrs)|(death at one week)|(death at the 168th hour)|(death at the 168th hr)|(death at the 168thhr )|(death at the 1st week)|(death at the eighth day)|(death at the first week)|(death before 8 days)|(1-week death)|(1-week deceased)|(1-week died)|(1-week dying)|(1-week fatality)|(1-week acute mortality)|(1wk death)|(1-wk death)|(1wk deceased)|(1-wk deceased)|(1wk died)|(1-wk died)|(1wk dying)|(1-wk dying)|(1wk fatality)|(1-wk fatality)|(1wk acute mortality)|(1-wk acute mortality)|(7 days death)|(7 days deceased)|(7 days died)|(7 days dying)|(7 days fatality)|(7 days acute mortality)|(7days death)|(7days deceased)|(7days died)|(7days dying)|(7days fatality)|(7days acute mortality)|(7th day death)|(7th day deceased)|(7th day died)|(7th day dying)|(7th day fatality)|(7th day acute mortality)|(death ahead of the 2nd week)|(death ahead of the second week)|(death at 1 week)|(death at 168 hours)|(death at 168 hrs)|(death at 168hrs)|(death at one week)|(death at the 168th hour)|(death at the 168th hr)|(death at the 168thhr )|(death at the 1st week)|(death at the eighth day)|(death at the first week)|(death before 8 days)|(1-week death)|(1-week deceased)|(1-week died)|(1-week dying)|(1-week fatality)|(1-week acute-mortality)|(1wk death)|(1-wk death)|(1wk deceased)|(1-wk deceased)|(1wk died)|(1-wk died)|(1wk dying)|(1-wk dying)|(1wk fatality)|(1-wk fatality)|(1wk acute-mortality)|(1-wk acute-mortality)|(7 days death)|(7 days deceased)|(7 days died)|(7 days dying)|(7 days fatality)|(7 days acute-mortality)|(7days death)|(7days deceased)|(7days died)|(7days dying)|(7days fatality)|(7days acute-mortality)|(7th day death)|(7th day deceased)|(7th day died)|(7th day dying)|(7th day fatality)|(7th day acute-mortality)|(death ahead of the 2nd week)|(death ahead of the second week)|(death at 1 week)|(death at 168 hours)|(death at 168 hrs)|(death at 168hrs)|(death at one week)|(death at the 168th hour)|(death at the 168th hr)|(death at the 168thhr )|(death at the 1st week)|(death at the eighth day)|(death at the first week)|(death before 8 days)|(fatality within 0 to seven days )|(fatality within 1 week)|(fatality within 1 week)|(fatality within 168 hours)|(fatality within 168 hrs)|(fatality within 168hrs)|(fatality within 1st 7 days)|(fatality within 7 days)|(fatality within a week)|(fatality within one hundred and sixty eight hours)|(fatality within one week)|(fatality within one-week)|(fatality within one-wk)|(fatality within seven days)|(fatality within the 1st 168 hours)|(fatality within the 1st 168 hrs)|(fatality within the 1st 168hrs)|(fatality within the 1st seven days)|(fatality within the 1st week)|(fatality within the first 168 hours)|(fatality within the first 168 hrs)|(fatality within the first 168hrs)|(fatality within the first 7 days)|(fatality within the first seven days)|(fatality within the first week)|(fatality within zero to 7 days)|(fatality within zero to seven days)|(case mortality ahead of the 2nd week)|(case mortality ahead of the second wee)|(case mortality at 1 week)|(case mortality at 168 hours)|(case mortality at 168 hrs)|(case mortality at 168hrs)|(case mortality at one week)|(fatality within 0 to seven days )|(fatality within 1 week)|(fatality within 1 week)|(fatality within 168 hours)|(fatality within 168 hrs)|(fatality within 168hrs)|(fatality within 1st 7 days)|(fatality within 7 days)|(fatality within a week)|(fatality within one hundred and sixty eight hours)|(fatality within one week)|(fatality within one-week)|(fatality within one-wk)|(fatality within seven days)|(fatality within the 1st 168 hours)|(fatality within the 1st 168 hrs)|(fatality within the 1st 168hrs)|(fatality within the 1st seven days)|(fatality within the 1st week)|(fatality within the first 168 hours)|(fatality within the first 168 hrs)|(fatality within the first 168hrs)|(fatality within the first 7 days)|(fatality within the first seven days)|(fatality within the first week)|(fatality within zero to 7 days)|(fatality within zero to seven days)|(case-mortality ahead of the 2nd week)|(case-mortality ahead of the second wee)|(case-mortality at 1 week)|(case-mortality at 168 hours)|(case-mortality at 168 hrs)|(case-mortality at 168hrs)|(case-mortality at one week)|(fatality within 0 to seven days )|(fatality within 1 week)|(fatality within 1 week)|(fatality within 168 hours)|(fatality within 168 hrs)|(fatality within 168hrs)|(fatality within 1st 7 days)|(fatality within 7 days)|(fatality within a week)|(fatality within one hundred and sixty eight hours)|(fatality within one week)|(fatality within one-week)|(fatality within one-wk)|(fatality within seven days)|(fatality within the 1st 168 hours)|(fatality within the 1st 168 hrs)|(fatality within the 1st 168hrs)|(fatality within the 1st seven days)|(fatality within the 1st week)|(fatality within the first 168 hours)|(fatality within the first 168 hrs)|(fatality within the first 168hrs)|(fatality within the first 7 days)|(fatality within the first seven days)|(fatality within the first week)|(fatality within zero to 7 days)|(fatality within zero to seven days)|(acute mortality ahead of the 2nd week)|(acute mortality ahead of the second wee)|(acute mortality at 1 week)|(acute mortality at 168 hours)|(acute mortality at 168 hrs)|(acute mortality at 168hrs)|(acute mortality at one week)|(fatality within 0 to seven days )|(fatality within 1 week)|(fatality within 1 week)|(fatality within 168 hours)|(fatality within 168 hrs)|(fatality within 168hrs)|(fatality within 1st 7 days)|(fatality within 7 days)|(fatality within a week)|(fatality within one hundred and sixty eight hours)|(fatality within one week)|(fatality within one-week)|(fatality within one-wk)|(fatality within seven days)|(fatality within the 1st 168 hours)|(fatality within the 1st 168 hrs)|(fatality within the 1st 168hrs)|(fatality within the 1st seven days)|(fatality within the 1st week)|(fatality within the first 168 hours)|(fatality within the first 168 hrs)|(fatality within the first 168hrs)|(fatality within the first 7 days)|(fatality within the first seven days)|(fatality within the first week)|(fatality within zero to 7 days)|(fatality within zero to seven days)|(acute-mortality ahead of the 2nd week)|(acute-mortality ahead of the second wee)|(acute-mortality at 1 week)|(acute-mortality at 168 hours)|(acute-mortality at 168 hrs)|(acute-mortality at 168hrs)|(acute-mortality at one week)|(case mortality at the 168th hour)|(case mortality at the 168th hr)|(case mortality at the 168thhr)|(case mortality at the 1st week)|(case mortality at the eighth day)|(case mortality at the first week)|(case mortality before 8 days)|(case mortality before the 2nd week)|(case mortality before the 8th day)|(case mortality before the eighth day)|(case mortality before the second week)|(case mortality by 1 week)|(case mortality by one week)|(case mortality by the 2nd week)|(case mortality by the end of the 1st week)|(case mortality by the end of the first week)|(case mortality by the second week)|(case mortality during 0 to 7 days)|(case mortality during 0 to seven days)|(case mortality during 1 week)|(case mortality during 1 week )|(case mortality during 168 hrs)|(case mortality during 168hrs)|(case mortality during 1st 7 days)|(case mortality during advance of the 2nd week)|(case mortality during advance of the second week)|(case mortality during one hundred and sixty eight hours)|(case mortality during one week)|(case mortality during one-week)|(case-mortality at the 168th hour)|(case-mortality at the 168th hr)|(case-mortality at the 168thhr)|(case-mortality at the 1st week)|(case-mortality at the eighth day)|(case-mortality at the first week)|(case-mortality before 8 days)|(case-mortality before the 2nd week)|(case-mortality before the 8th day)|(case-mortality before the eighth day)|(case-mortality before the second week)|(case-mortality by 1 week)|(case-mortality by one week)|(case-mortality by the 2nd week)|(case-mortality by the end of the 1st week)|(case-mortality by the end of the first week)|(case-mortality by the second week)|(case-mortality during 0 to 7 days)|(case-mortality during 0 to seven days)|(case-mortality during 1 week)|(case-mortality during 1 week )|(case-mortality during 168 hrs)|(case-mortality during 168hrs)|(case-mortality during 1st 7 days)|(case-mortality during advance of the 2nd week)|(case-mortality during advance of the second week)|(case-mortality during one hundred and sixty eight hours)|(case-mortality during one week)|(case-mortality during one-week)|(acute mortality at the 168th hour)|(acute mortality at the 168th hr)|(acute mortality at the 168thhr)|(acute mortality at the 1st week)|(acute mortality at the eighth day)|(acute mortality at the first week)|(acute mortality before 8 days)|(acute mortality before the 2nd week)|(acute mortality before the 8th day)|(acute mortality before the eighth day)|(acute mortality before the second week)|(acute mortality by 1 week)|(acute mortality by one week)|(acute mortality by the 2nd week)|(acute mortality by the end of the 1st week)|(acute mortality by the end of the first week)|(acute mortality by the second week)|(acute mortality during 0 to 7 days)|(acute mortality during 0 to seven days)|(acute mortality during 1 week)|(acute mortality during 1 week )|(acute mortality during 168 hrs)|(acute mortality during 168hrs)|(acute mortality during 1st 7 days)|(acute mortality during advance of the 2nd week)|(acute mortality during advance of the second week)|(acute mortality during one hundred and sixty eight hours)|(acute mortality during one week)|(acute mortality during one-week)|(acute-mortality at the 168th hour)|(acute-mortality at the 168th hr)|(acute-mortality at the 168thhr)|(acute-mortality at the 1st week)|(acute-mortality at the eighth day)|(acute-mortality at the first week)|(acute-mortality before 8 days)|(acute-mortality before the 2nd week)|(acute-mortality before the 8th day)|(acute-mortality before the eighth day)|(acute-mortality before the second week)|(acute-mortality by 1 week)|(acute-mortality by one week)|(acute-mortality by the 2nd week)|(acute-mortality by the end of the 1st week)|(acute-mortality by the end of the first week)|(acute-mortality by the second week)|(acute-mortality during 0 to 7 days)|(acute-mortality during 0 to seven days)|(acute-mortality during 1 week)|(acute-mortality during 1 week )|(acute-mortality during 168 hrs)|(acute-mortality during 168hrs)|(acute-mortality during 1st 7 days)|(acute-mortality during advance of the 2nd week)|(acute-mortality during advance of the second week)|(acute-mortality during one hundred and sixty eight hours)|(acute-mortality during one week)|(acute-mortality during one-week)|(case mortality during one-wk)|(case mortality during seven days)|(case mortality during the 1st 168 hours)|(case mortality during the 1st 168 hrs)|(case mortality during the 1st 168hrs)|(case mortality during the 1st seven days )|(case mortality during the 1st week)|(case mortality during the first 168 hours)|(case mortality during the first 168 hrs)|(case mortality during the first 168hrs)|(case mortality during the first 7 days)|(case mortality during the first seven days)|(case mortality during the first week)|(case mortality during zero to 7 days)|(case mortality during zero to seven days)|(case mortality earlier than the 2nd week)|(case mortality earlier than the second week)|(case mortality equal to 7 days)|(case mortality in 0 to 7 days)|(case mortality in 0 to seven days)|(case mortality in 1 week)|(case mortality in 1 week )|(case mortality in 168 hrs)|(case mortality in 168hrs)|(case mortality in 1st 7 days)|(case mortality in advance of the 2nd week)|(case mortality in advance of the second week)|(case mortality in one hundred and sixty eight hours)|(case-mortality during one-wk)|(case-mortality during seven days)|(case-mortality during the 1st 168 hours)|(case-mortality during the 1st 168 hrs)|(case-mortality during the 1st 168hrs)|(case-mortality during the 1st seven days )|(case-mortality during the 1st week)|(case-mortality during the first 168 hours)|(case-mortality during the first 168 hrs)|(case-mortality during the first 168hrs)|(case-mortality during the first 7 days)|(case-mortality during the first seven days)|(case-mortality during the first week)|(case-mortality during zero to 7 days)|(case-mortality during zero to seven days)|(case-mortality earlier than the 2nd week)|(case-mortality earlier than the second week)|(case-mortality equal to 7 days)|(case-mortality in 0 to 7 days)|(case-mortality in 0 to seven days)|(case-mortality in 1 week)|(case-mortality in 1 week )|(case-mortality in 168 hrs)|(case-mortality in 168hrs)|(case-mortality in 1st 7 days)|(case-mortality in advance of the 2nd week)|(case-mortality in advance of the second week)|(case-mortality in one hundred and sixty eight hours)|(acute mortality during one-wk)|(acute mortality during seven days)|(acute mortality during the 1st 168 hours)|(acute mortality during the 1st 168 hrs)|(acute mortality during the 1st 168hrs)|(acute mortality during the 1st seven days )|(acute mortality during the 1st week)|(acute mortality during the first 168 hours)|(acute mortality during the first 168 hrs)|(acute mortality during the first 168hrs)|(acute mortality during the first 7 days)|(acute mortality during the first seven days)|(acute mortality during the first week)|(acute mortality during zero to 7 days)|(acute mortality during zero to seven days)|(acute mortality earlier than the 2nd week)|(acute mortality earlier than the second week)|(acute mortality equal to 7 days)|(acute mortality in 0 to 7 days)|(acute mortality in 0 to seven days)|(acute mortality in 1 week)|(acute mortality in 1 week )|(acute mortality in 168 hrs)|(acute mortality in 168hrs)|(acute mortality in 1st 7 days)|(acute mortality in advance of the 2nd week)|(acute mortality in advance of the second week)|(acute mortality in one hundred and sixty eight hours)|(acute-mortality during one-wk)|(acute-mortality during seven days)|(acute-mortality during the 1st 168 hours)|(acute-mortality during the 1st 168 hrs)|(acute-mortality during the 1st 168hrs)|(acute-mortality during the 1st seven days )|(acute-mortality during the 1st week)|(acute-mortality during the first 168 hours)|(acute-mortality during the first 168 hrs)|(acute-mortality during the first 168hrs)|(acute-mortality during the first 7 days)|(acute-mortality during the first seven days)|(acute-mortality during the first week)|(acute-mortality during zero to 7 days)|(acute-mortality during zero to seven days)|(acute-mortality earlier than the 2nd week)|(acute-mortality earlier than the second week)|(acute-mortality equal to 7 days)|(acute-mortality in 0 to 7 days)|(acute-mortality in 0 to seven days)|(acute-mortality in 1 week)|(acute-mortality in 1 week )|(acute-mortality in 168 hrs)|(acute-mortality in 168hrs)|(acute-mortality in 1st 7 days)|(acute-mortality in advance of the 2nd week)|(acute-mortality in advance of the second week)|(acute-mortality in one hundred and sixty eight hours)|(case mortality in one week)|(case mortality in one-week)|(case mortality in one-wk)|(case mortality in seven days)|(case mortality in the 1st 168 hours)|(case mortality in the 1st 168 hrs)|(case mortality in the 1st 168hrs)|(case mortality in the 1st seven days )|(case mortality in the 1st week)|(case mortality in the first 168 hours)|(case mortality in the first 168 hrs)|(case mortality in the first 168hrs)|(case mortality in the first 7 days)|(case mortality in the first seven days)|(case mortality in the first week)|(case mortality in zero to 7 days)|(case mortality in zero to seven days)|(case mortality less than 1 week)|(case mortality less than 169 hours)|(case mortality less than 8 days)|(case mortality less than one week)|(case mortality less than or equal to 7 days)|(case mortality less than or equal to seven days)|(case mortality less than the 1st week)|(case mortality less than the first week)|(case-mortality in one week)|(case-mortality in one-week)|(case-mortality in one-wk)|(case-mortality in seven days)|(case-mortality in the 1st 168 hours)|(case-mortality in the 1st 168 hrs)|(case-mortality in the 1st 168hrs)|(case-mortality in the 1st seven days )|(case-mortality in the 1st week)|(case-mortality in the first 168 hours)|(case-mortality in the first 168 hrs)|(case-mortality in the first 168hrs)|(case-mortality in the first 7 days)|(case-mortality in the first seven days)|(case-mortality in the first week)|(case-mortality in zero to 7 days)|(case-mortality in zero to seven days)|(case-mortality less than 1 week)|(case-mortality less than 169 hours)|(case-mortality less than 8 days)|(case-mortality less than one week)|(case-mortality less than or equal to 7 days)|(case-mortality less than or equal to seven days)|(case-mortality less than the 1st week)|(case-mortality less than the first week)|(acute mortality in one week)|(acute mortality in one-week)|(acute mortality in one-wk)|(acute mortality in seven days)|(acute mortality in the 1st 168 hours)|(acute mortality in the 1st 168 hrs)|(acute mortality in the 1st 168hrs)|(acute mortality in the 1st seven days )|(acute mortality in the 1st week)|(acute mortality in the first 168 hours)|(acute mortality in the first 168 hrs)|(acute mortality in the first 168hrs)|(acute mortality in the first 7 days)|(acute mortality in the first seven days)|(acute mortality in the first week)|(acute mortality in zero to 7 days)|(acute mortality in zero to seven days)|(acute mortality less than 1 week)|(acute mortality less than 169 hours)|(acute mortality less than 8 days)|(acute mortality less than one week)|(acute mortality less than or equal to 7 days)|(acute mortality less than or equal to seven days)|(acute mortality less than the 1st week)|(acute mortality less than the first week)|(acute-mortality in one week)|(acute-mortality in one-week)|(acute-mortality in one-wk)|(acute-mortality in seven days)|(acute-mortality in the 1st 168 hours)|(acute-mortality in the 1st 168 hrs)|(acute-mortality in the 1st 168hrs)|(acute-mortality in the 1st seven days )|(acute-mortality in the 1st week)|(acute-mortality in the first 168 hours)|(acute-mortality in the first 168 hrs)|(acute-mortality in the first 168hrs)|(acute-mortality in the first 7 days)|(acute-mortality in the first seven days)|(acute-mortality in the first week)|(acute-mortality in zero to 7 days)|(acute-mortality in zero to seven days)|(acute-mortality less than 1 week)|(acute-mortality less than 169 hours)|(acute-mortality less than 8 days)|(acute-mortality less than one week)|(acute-mortality less than or equal to 7 days)|(acute-mortality less than or equal to seven days)|(acute-mortality less than the 1st week)|(acute-mortality less than the first week)|(case mortality on the 7th day)|(case mortality on the seventh day)|(case mortality over 1 week)|(case mortality over one week)|(case mortality previous to the 2nd week)|(case mortality previous to the second week)|(case mortality prior to the 2nd week)|(case mortality prior to the second week)|(case mortality sooner than the 2nd week)|(case mortality sooner than the second week)|(case mortality up to 7 days)|(case mortality up to day 7)|(case mortality up to day seven)|(case mortality up to seven days)|(case mortality up to the 7th day)|(case mortality up to the seventh day)|(case mortality within 0 to 7 days)|(case mortality within 0 to seven days )|(case mortality within 1 week)|(case mortality within 1 week )|(case mortality within 168 hours)|(case mortality within 168 hrs)|(case mortality within 168hrs)|(case mortality within 1st 7 days)|(case mortality within 7 days)|(case mortality within a week)|(case mortality within one hundred and sixty eight hours)|(case mortality within one week)|(case mortality within one-week)|(case mortality within one-wk)|(case mortality within seven days)|(case mortality within the 1st 168 hours)|(case mortality within the 1st 168 hrs)|(case mortality within the 1st 168hrs)|(case mortality within the 1st seven days)|(case mortality within the 1st week)|(case mortality within the first 168 hours)|(case mortality within the first 168 hrs)|(case mortality within the first 168hrs)|(case mortality within the first 7 days)|(case mortality within the first seven days)|(case mortality within the first week)|(case mortality within zero to 7 days)|(case mortality within zero to seven days)|(case-mortality on the 7th day)|(case-mortality on the seventh day)|(case-mortality over 1 week)|(case-mortality over one week)|(case-mortality previous to the 2nd week)|(case-mortality previous to the second week)|(case-mortality prior to the 2nd week)|(case-mortality prior to the second week)|(case-mortality sooner than the 2nd week)|(case-mortality sooner than the second week)|(case-mortality up to 7 days)|(case-mortality up to day 7)|(case-mortality up to day seven)|(case-mortality up to seven days)|(case-mortality up to the 7th day)|(case-mortality up to the seventh day)|(case-mortality within 0 to 7 days)|(case-mortality within 0 to seven days )|(case-mortality within 1 week)|(case-mortality within 1 week )|(case-mortality within 168 hours)|(case-mortality within 168 hrs)|(case-mortality within 168hrs)|(case-mortality within 1st 7 days)|(case-mortality within 7 days)|(case-mortality within a week)|(case-mortality within one hundred and sixty eight hours)|(case-mortality within one week)|(case-mortality within one-week)|(case-mortality within one-wk)|(case-mortality within seven days)|(case-mortality within the 1st 168 hours)|(case-mortality within the 1st 168 hrs)|(case-mortality within the 1st 168hrs)|(case-mortality within the 1st seven days)|(case-mortality within the 1st week)|(case-mortality within the first 168 hours)|(case-mortality within the first 168 hrs)|(case-mortality within the first 168hrs)|(case-mortality within the first 7 days)|(case-mortality within the first seven days)|(case-mortality within the first week)|(case-mortality within zero to 7 days)|(case-mortality within zero to seven days)|(acute mortality on the 7th day)|(acute mortality on the seventh day)|(acute mortality over 1 week)|(acute mortality over one week)|(acute mortality previous to the 2nd week)|(acute mortality previous to the second week)|(acute mortality prior to the 2nd week)|(acute mortality prior to the second week)|(acute mortality sooner than the 2nd week)|(acute mortality sooner than the second week)|(acute mortality up to 7 days)|(acute mortality up to day 7)|(acute mortality up to day seven)|(acute mortality up to seven days)|(acute mortality up to the 7th day)|(acute mortality up to the seventh day)|(acute mortality within 0 to 7 days)|(acute mortality within 0 to seven days )|(acute mortality within 1 week)|(acute mortality within 1 week )|(acute mortality within 168 hours)|(acute mortality within 168 hrs)|(acute mortality within 168hrs)|(acute mortality within 1st 7 days)|(acute mortality within 7 days)|(acute mortality within a week)|(acute mortality within one hundred and sixty eight hours)|(acute mortality within one week)|(acute mortality within one-week)|(acute mortality within one-wk)|(acute mortality within seven days)|(acute mortality within the 1st 168 hours)|(acute mortality within the 1st 168 hrs)|(acute mortality within the 1st 168hrs)|(acute mortality within the 1st seven days)|(acute mortality within the 1st week)|(acute mortality within the first 168 hours)|(acute mortality within the first 168 hrs)|(acute mortality within the first 168hrs)|(acute mortality within the first 7 days)|(acute mortality within the first seven days)|(acute mortality within the first week)|(acute mortality within zero to 7 days)|(acute mortality within zero to seven days)|(acute-mortality on the 7th day)|(acute-mortality on the seventh day)|(acute-mortality over 1 week)|(acute-mortality over one week)|(acute-mortality previous to the 2nd week)|(acute-mortality previous to the second week)|(acute-mortality prior to the 2nd week)|(acute-mortality prior to the second week)|(acute-mortality sooner than the 2nd week)|(acute-mortality sooner than the second week)|(acute-mortality up to 7 days)|(acute-mortality up to day 7)|(acute-mortality up to day seven)|(acute-mortality up to seven days)|(acute-mortality up to the 7th day)|(acute-mortality up to the seventh day)|(acute-mortality within 0 to 7 days)|(acute-mortality within 0 to seven days )|(acute-mortality within 1 week)|(acute-mortality within 1 week )|(acute-mortality within 168 hours)|(acute-mortality within 168 hrs)|(acute-mortality within 168hrs)|(acute-mortality within 1st 7 days)|(acute-mortality within 7 days)|(acute-mortality within a week)|(acute-mortality within one hundred and sixty eight hours)|(acute-mortality within one week)|(acute-mortality within one-week)|(acute-mortality within one-wk)|(acute-mortality within seven days)|(acute-mortality within the 1st 168 hours)|(acute-mortality within the 1st 168 hrs)|(acute-mortality within the 1st 168hrs)|(acute-mortality within the 1st seven days)|(acute-mortality within the 1st week)|(acute-mortality within the first 168 hours)|(acute-mortality within the first 168 hrs)|(acute-mortality within the first 168hrs)|(acute-mortality within the first 7 days)|(acute-mortality within the first seven days)|(acute-mortality within the first week)|(acute-mortality within zero to 7 days)|(acute-mortality within zero to seven days)|(case mortality sooner than the first week)|(case mortality sooner than the 1^st^ week)|(case mortality sooner than the 1^st^ wk)|(case mortality sooner than 7 days)|(case mortality sooner than seven days)|(case mortality sooner than the 7^th^ day)|(case mortality sooner than the seventh day)|(24 hour case mortality)|(24-hour case mortality)|(24-hr case mortality)|(24hr case mortality)|(24 hr case mortality)|(twenty four hour case mortality)|(twenty-four hour case mortality)|(case mortality at one day)|(case mortality at 1 day)|(case mortality in one day)|(case mortality in 1 day)|(case mortality at 24 hours)|(case mortality in 24 hours)|(case mortality in twenty four hours)|(case mortality in twenty-four hours)|(case mortality at twenty-four hours)|(case mortality at twenty four hours)|(48 hour case mortality)|(48-hour case mortality)|(48-hr case mortality)|(48hr case mortality)|(48 hr case mortality)|(forty eight hour case mortality)|(forty-eight hour case mortality)|(case mortality at two days)|(case mortality at 2 days)|(case-mortality sooner than the first week)|(case-mortality sooner than the 1^st^ week)|(case-mortality sooner than the 1^st^ wk)|(case-mortality sooner than 7 days)|(case-mortality sooner than seven days)|(case-mortality sooner than the 7^th^ day)|(case-mortality sooner than the seventh day)|(24 hour case-mortality)|(24-hour case-mortality)|(24-hr case-mortality)|(24hr case-mortality)|(24 hr case-mortality)|(twenty four hour case-mortality)|(twenty-four hour case-mortality)|(case-mortality at one day)|(case-mortality at 1 day)|(case-mortality in one day)|(case-mortality in 1 day)|(case-mortality at 24 hours)|(case-mortality in 24 hours)|(case-mortality in twenty four hours)|(case-mortality in twenty-four hours)|(case-mortality at twenty-four hours)|(case-mortality at twenty four hours)|(48 hour case-mortality)|(48-hour case-mortality)|(48-hr case-mortality)|(48hr case-mortality)|(48 hr case-mortality)|(forty eight hour case-mortality)|(forty-eight hour case-mortality)|(case-mortality at two days)|(case-mortality at 2 days)|(acute mortality sooner than the first week)|(acute mortality sooner than the 1^st^ week)|(acute mortality sooner than the 1^st^ wk)|(acute mortality sooner than 7 days)|(acute mortality sooner than seven days)|(acute mortality sooner than the 7^th^ day)|(acute mortality sooner than the seventh day)|(24 hour acute mortality)|(24-hour acute mortality)|(24-hr acute mortality)|(24hr acute mortality)|(24 hr acute mortality)|(twenty four hour acute mortality)|(twenty-four hour acute mortality)|(acute mortality at one day)|(acute mortality at 1 day)|(acute mortality in one day)|(acute mortality in 1 day)|(acute mortality at 24 hours)|(acute mortality in 24 hours)|(acute mortality in twenty four hours)|(acute mortality in twenty-four hours)|(acute mortality at twenty-four hours)|(acute mortality at twenty four hours)|(48 hour acute mortality)|(48-hour acute mortality)|(48-hr acute mortality)|(48hr acute mortality)|(48 hr acute mortality)|(forty eight hour acute mortality)|(forty-eight hour acute mortality)|(acute mortality at two days)|(acute mortality at 2 days)|(acute-mortality sooner than the first week)|(acute-mortality sooner than the 1^st^ week)|(acute-mortality sooner than the 1^st^ wk)|(acute-mortality sooner than 7 days)|(acute-mortality sooner than seven days)|(acute-mortality sooner than the 7^th^ day)|(acute-mortality sooner than the seventh day)|(24 hour acute-mortality)|(24-hour acute-mortality)|(24-hr acute-mortality)|(24hr acute-mortality)|(24 hr acute-mortality)|(twenty four hour acute-mortality)|(twenty-four hour acute-mortality)|(acute-mortality at one day)|(acute-mortality at 1 day)|(acute-mortality in one day)|(acute-mortality in 1 day)|(acute-mortality at 24 hours)|(acute-mortality in 24 hours)|(acute-mortality in twenty four hours)|(acute-mortality in twenty-four hours)|(acute-mortality at twenty-four hours)|(acute-mortality at twenty four hours)|(48 hour acute-mortality)|(48-hour acute-mortality)|(48-hr acute-mortality)|(48hr acute-mortality)|(48 hr acute-mortality)|(forty eight hour acute-mortality)|(forty-eight hour acute-mortality)|(acute-mortality at two days)|(acute-mortality at 2 days)|(case mortality in two days)|(case mortality in 2 days)|(case mortality at 48 hours)|(case mortality in 48 hours)|(case mortality in forty eight hours)|(case mortality in forty-eight hours)|(case mortality at forty eight hours)|(case mortality at forty-eight hours)|(72 hour case mortality)|(72-hour case mortality)|(72-hr case mortality)|(72hr case mortality)|(72 hr case mortality)|(seventy two hour case mortality)|(seventy-two hour case mortality)|(case mortality at three days)|(case mortality at 3 days)|(case mortality in three days)|(case mortality in 3 days)|(case mortality at 72 hours)|(case mortality in 72 hours)|(case mortality in seventy two hours)|(case mortality in seventy-two hours)|(case mortality at seventy two hours)|(case mortality at seventy-two hours)|(96 hour case mortality)|(96-hour case mortality)|(96-hr case mortality)|(96hr case mortality)|(96 hr case mortality)|(ninety six hour case mortality)|(ninety-six hour case mortality)|(case-mortality in two days)|(case-mortality in 2 days)|(case-mortality at 48 hours)|(case-mortality in 48 hours)|(case-mortality in forty eight hours)|(case-mortality in forty-eight hours)|(case-mortality at forty eight hours)|(case-mortality at forty-eight hours)|(72 hour case-mortality)|(72-hour case-mortality)|(72-hr case-mortality)|(72hr case-mortality)|(72 hr case-mortality)|(seventy two hour case-mortality)|(seventy-two hour case-mortality)|(case-mortality at three days)|(case-mortality at 3 days)|(case-mortality in three days)|(case-mortality in 3 days)|(case-mortality at 72 hours)|(case-mortality in 72 hours)|(case-mortality in seventy two hours)|(case-mortality in seventy-two hours)|(case-mortality at seventy two hours)|(case-mortality at seventy-two hours)|(96 hour case-mortality)|(96-hour case-mortality)|(96-hr case-mortality)|(96hr case-mortality)|(96 hr case-mortality)|(ninety six hour case-mortality)|(ninety-six hour case-mortality)|(acute mortality in two days)|(acute mortality in 2 days)|(acute mortality at 48 hours)|(acute mortality in 48 hours)|(acute mortality in forty eight hours)|(acute mortality in forty-eight hours)|(acute mortality at forty eight hours)|(acute mortality at forty-eight hours)|(72 hour acute mortality)|(72-hour acute mortality)|(72-hr acute mortality)|(72hr acute mortality)|(72 hr acute mortality)|(seventy two hour acute mortality)|(seventy-two hour acute mortality)|(acute mortality at three days)|(acute mortality at 3 days)|(acute mortality in three days)|(acute mortality in 3 days)|(acute mortality at 72 hours)|(acute mortality in 72 hours)|(acute mortality in seventy two hours)|(acute mortality in seventy-two hours)|(acute mortality at seventy two hours)|(acute mortality at seventy-two hours)|(96 hour acute mortality)|(96-hour acute mortality)|(96-hr acute mortality)|(96hr acute mortality)|(96 hr acute mortality)|(ninety six hour acute mortality)|(ninety-six hour acute mortality)|(acute-mortality in two days)|(acute-mortality in 2 days)|(acute-mortality at 48 hours)|(acute-mortality in 48 hours)|(acute-mortality in forty eight hours)|(acute-mortality in forty-eight hours)|(acute-mortality at forty eight hours)|(acute-mortality at forty-eight hours)|(72 hour acute-mortality)|(72-hour acute-mortality)|(72-hr acute-mortality)|(72hr acute-mortality)|(72 hr acute-mortality)|(seventy two hour acute-mortality)|(seventy-two hour acute-mortality)|(acute-mortality at three days)|(acute-mortality at 3 days)|(acute-mortality in three days)|(acute-mortality in 3 days)|(acute-mortality at 72 hours)|(acute-mortality in 72 hours)|(acute-mortality in seventy two hours)|(acute-mortality in seventy-two hours)|(acute-mortality at seventy two hours)|(acute-mortality at seventy-two hours)|(96 hour acute-mortality)|(96-hour acute-mortality)|(96-hr acute-mortality)|(96hr acute-mortality)|(96 hr acute-mortality)|(ninety six hour acute-mortality)|(ninety-six hour acute-mortality)|(case mortality at 4 days)|(case mortality at 4 days)|(case mortality in four days)|(case mortality in 4 days)|(case mortality at 96 hours)|(case mortality in 96 hours)|(case mortality in ninety six hours)|(case mortality in ninety-six hours)|(case mortality at ninety six hours)|(case mortality at ninety-six hours)|(120 hour case mortality)|(120-hour case mortality)|(120-hr case mortality)|(120hr case mortality)|(120 hr case mortality)|(one hundred and twenty hour case mortality)|(case mortality at five days)|(case mortality at 5 days)|(case mortality in five days)|(case mortality in 5 days)|(case mortality at one hundred and twenty hours)|(case mortality in one hundred and twenty hours)|(case mortality at one hundred and twenty hours)|(144 hour case mortality)|(144-hour case mortality)|(144-hr case mortality)|(144hr case mortality)|(144 hr case mortality)|(one hundred and forty four hour case mortality)|(one hundred and forty-four hour case mortality)|(case mortality at six days)|(case mortality at 6 days)|(case mortality in six days)|(case mortality in 6 days)|(case mortality at 144 hours)|(case mortality in 144 hours)|(case-mortality at 4 days)|(case-mortality at 4 days)|(case-mortality in four days)|(case-mortality in 4 days)|(case-mortality at 96 hours)|(case-mortality in 96 hours)|(case-mortality in ninety six hours)|(case-mortality in ninety-six hours)|(case-mortality at ninety six hours)|(case-mortality at ninety-six hours)|(120 hour case-mortality)|(120-hour case-mortality)|(120-hr case-mortality)|(120hr case-mortality)|(120 hr case-mortality)|(one hundred and twenty hour case-mortality)|(case-mortality at five days)|(case-mortality at 5 days)|(case-mortality in five days)|(case-mortality in 5 days)|(case-mortality at one hundred and twenty hours)|(case-mortality in one hundred and twenty hours)|(case-mortality at one hundred and twenty hours)|(144 hour case-mortality)|(144-hour case-mortality)|(144-hr case-mortality)|(144hr case-mortality)|(144 hr case-mortality)|(one hundred and forty four hour case-mortality)|(one hundred and forty-four hour case-mortality)|(case-mortality at six days)|(case-mortality at 6 days)|(case-mortality in six days)|(case-mortality in 6 days)|(case-mortality at 144 hours)|(case-mortality in 144 hours)|(acute mortality at 4 days)|(acute mortality at 4 days)|(acute mortality in four days)|(acute mortality in 4 days)|(acute mortality at 96 hours)|(acute mortality in 96 hours)|(acute mortality in ninety six hours)|(acute mortality in ninety-six hours)|(acute mortality at ninety six hours)|(acute mortality at ninety-six hours)|(120 hour acute mortality)|(120-hour acute mortality)|(120-hr acute mortality)|(120hr acute mortality)|(120 hr acute mortality)|(one hundred and twenty hour acute mortality)|(acute mortality at five days)|(acute mortality at 5 days)|(acute mortality in five days)|(acute mortality in 5 days)|(acute mortality at one hundred and twenty hours)|(acute mortality in one hundred and twenty hours)|(acute mortality at one hundred and twenty hours)|(144 hour acute mortality)|(144-hour acute mortality)|(144-hr acute mortality)|(144hr acute mortality)|(144 hr acute mortality)|(one hundred and forty four hour acute mortality)|(one hundred and forty-four hour acute mortality)|(acute mortality at six days)|(acute mortality at 6 days)|(acute mortality in six days)|(acute mortality in 6 days)|(acute mortality at 144 hours)|(acute mortality in 144 hours)|(acute-mortality at 4 days)|(acute-mortality at 4 days)|(acute-mortality in four days)|(acute-mortality in 4 days)|(acute-mortality at 96 hours)|(acute-mortality in 96 hours)|(acute-mortality in ninety six hours)|(acute-mortality in ninety-six hours)|(acute-mortality at ninety six hours)|(acute-mortality at ninety-six hours)|(120 hour acute-mortality)|(120-hour acute-mortality)|(120-hr acute-mortality)|(120hr acute-mortality)|(120 hr acute-mortality)|(one hundred and twenty hour acute-mortality)|(acute-mortality at five days)|(acute-mortality at 5 days)|(acute-mortality in five days)|(acute-mortality in 5 days)|(acute-mortality at one hundred and twenty hours)|(acute-mortality in one hundred and twenty hours)|(acute-mortality at one hundred and twenty hours)|(144 hour acute-mortality)|(144-hour acute-mortality)|(144-hr acute-mortality)|(144hr acute-mortality)|(144 hr acute-mortality)|(one hundred and forty four hour acute-mortality)|(one hundred and forty-four hour acute-mortality)|(acute-mortality at six days)|(acute-mortality at 6 days)|(acute-mortality in six days)|(acute-mortality in 6 days)|(acute-mortality at 144 hours)|(acute-mortality in 144 hours)|(case mortality in one hundred and forty four hours)|(case mortality in one hundred and forty-four hours)|(case mortality at one hundred and forty four hours)|(case mortality at one hundred and forty-four hours)|(168 hour case mortality)|(168-hour case mortality)|(168-hr case mortality)|(168hr case mortality)|(168 hr case mortality)|(one hundred and sixty eight hour case mortality)|(one hundred and sixty-eight hour case mortality)|(case mortality at seven days)|(case mortality at 7 days)|(case mortality in seven days)|(case mortality in 7 days)|(case mortality at 168 hours)|(case mortality in 168 hours)|(case mortality in one hundred and sixty eight hours)|(case mortality in one hundred and sixty-eight hours)|(case mortality at one hundred and sixty eight hours)|(case mortality at one hundred and sixty-eight hours)|(fatality sooner than the first week)|(fatality sooner than the 1^st^ week)|(fatality sooner than the 1^st^ wk)|(case-mortality in one hundred and forty four hours)|(case-mortality in one hundred and forty-four hours)|(case-mortality at one hundred and forty four hours)|(case-mortality at one hundred and forty-four hours)|(168 hour case-mortality)|(168-hour case-mortality)|(168-hr case-mortality)|(168hr case-mortality)|(168 hr case-mortality)|(one hundred and sixty eight hour case-mortality)|(one hundred and sixty-eight hour case-mortality)|(case-mortality at seven days)|(case-mortality at 7 days)|(case-mortality in seven days)|(case-mortality in 7 days)|(case-mortality at 168 hours)|(case-mortality in 168 hours)|(case-mortality in one hundred and sixty eight hours)|(case-mortality in one hundred and sixty-eight hours)|(case-mortality at one hundred and sixty eight hours)|(case-mortality at one hundred and sixty-eight hours)|(fatality sooner than the first week)|(fatality sooner than the 1^st^ week)|(fatality sooner than the 1^st^ wk)|(acute mortality in one hundred and forty four hours)|(acute mortality in one hundred and forty-four hours)|(acute mortality at one hundred and forty four hours)|(acute mortality at one hundred and forty-four hours)|(168 hour acute mortality)|(168-hour acute mortality)|(168-hr acute mortality)|(168hr acute mortality)|(168 hr acute mortality)|(one hundred and sixty eight hour acute mortality)|(one hundred and sixty-eight hour acute mortality)|(acute mortality at seven days)|(acute mortality at 7 days)|(acute mortality in seven days)|(acute mortality in 7 days)|(acute mortality at 168 hours)|(acute mortality in 168 hours)|(acute mortality in one hundred and sixty eight hours)|(acute mortality in one hundred and sixty-eight hours)|(acute mortality at one hundred and sixty eight hours)|(acute mortality at one hundred and sixty-eight hours)|(fatality sooner than the first week)|(fatality sooner than the 1^st^ week)|(fatality sooner than the 1^st^ wk)|(acute-mortality in one hundred and forty four hours)|(acute-mortality in one hundred and forty-four hours)|(acute-mortality at one hundred and forty four hours)|(acute-mortality at one hundred and forty-four hours)|(168 hour acute-mortality)|(168-hour acute-mortality)|(168-hr acute-mortality)|(168hr acute-mortality)|(168 hr acute-mortality)|(one hundred and sixty eight hour acute-mortality)|(one hundred and sixty-eight hour acute-mortality)|(acute-mortality at seven days)|(acute-mortality at 7 days)|(acute-mortality in seven days)|(acute-mortality in 7 days)|(acute-mortality at 168 hours)|(acute-mortality in 168 hours)|(acute-mortality in one hundred and sixty eight hours)|(acute-mortality in one hundred and sixty-eight hours)|(acute-mortality at one hundred and sixty eight hours)|(acute-mortality at one hundred and sixty-eight hours)|(fatality sooner than the first week)|(fatality sooner than the 1^st^ week)|(fatality sooner than the 1^st^ wk)|(1-week death)|(1-week deceased)|(1-week died)|(1-week dying)|(1-week case fatality)|(1-week case mortality)|(1wk death)|(1-wk death)|(1wk deceased)|(1-wk deceased)|(1wk died)|(1-wk died)|(1wk dying)|(1-wk dying)|(1wk case fatality)|(1-wk case fatality)|(1wk case mortality)|(1-wk case mortality)|(7 days death)|(7 days deceased)|(7 days died)|(7 days dying)|(7 days case fatality)|(7 days case mortality)|(7days death)|(7days deceased)|(7days died)|(7days dying)|(7days case fatality)|(7days case mortality)|(7th day death)|(7th day deceased)|(7th day died)|(7th day dying)|(7th day case fatality)|(7th day case mortality)|(death ahead of the 2nd week)|(death ahead of the second week)|(death at 1 week)|(death at 168 hours)|(death at 168 hrs)|(death at 168hrs)|(death at one week)|(death at the 168th hour)|(death at the 168th hr)|(death at the 168thhr )|(death at the 1st week)|(death at the eighth day)|(death at the first week)|(death before 8 days)|(1-week death)|(1-week deceased)|(1-week died)|(1-week dying)|(1-week case fatality)|(1-week case-mortality)|(1wk death)|(1-wk death)|(1wk deceased)|(1-wk deceased)|(1wk died)|(1-wk died)|(1wk dying)|(1-wk dying)|(1wk case fatality)|(1-wk case fatality)|(1wk case-mortality)|(1-wk case-mortality)|(7 days death)|(7 days deceased)|(7 days died)|(7 days dying)|(7 days case fatality)|(7 days case-mortality)|(7days death)|(7days deceased)|(7days died)|(7days dying)|(7days case fatality)|(7days case-mortality)|(7th day death)|(7th day deceased)|(7th day died)|(7th day dying)|(7th day case fatality)|(7th day case-mortality)|(death ahead of the 2nd week)|(death ahead of the second week)|(death at 1 week)|(death at 168 hours)|(death at 168 hrs)|(death at 168hrs)|(death at one week)|(death at the 168th hour)|(death at the 168th hr)|(death at the 168thhr )|(death at the 1st week)|(death at the eighth day)|(death at the first week)|(death before 8 days)|(1-week death)|(1-week deceased)|(1-week died)|(1-week dying)|(1-week case fatality)|(1-week acute mortality)|(1wk death)|(1-wk death)|(1wk deceased)|(1-wk deceased)|(1wk died)|(1-wk died)|(1wk dying)|(1-wk dying)|(1wk case fatality)|(1-wk case fatality)|(1wk acute mortality)|(1-wk acute mortality)|(7 days death)|(7 days deceased)|(7 days died)|(7 days dying)|(7 days case fatality)|(7 days acute mortality)|(7days death)|(7days deceased)|(7days died)|(7days dying)|(7days case fatality)|(7days acute mortality)|(7th day death)|(7th day deceased)|(7th day died)|(7th day dying)|(7th day case fatality)|(7th day acute mortality)|(death ahead of the 2nd week)|(death ahead of the second week)|(death at 1 week)|(death at 168 hours)|(death at 168 hrs)|(death at 168hrs)|(death at one week)|(death at the 168th hour)|(death at the 168th hr)|(death at the 168thhr )|(death at the 1st week)|(death at the eighth day)|(death at the first week)|(death before 8 days)|(1-week death)|(1-week deceased)|(1-week died)|(1-week dying)|(1-week case fatality)|(1-week acute-mortality)|(1wk death)|(1-wk death)|(1wk deceased)|(1-wk deceased)|(1wk died)|(1-wk died)|(1wk dying)|(1-wk dying)|(1wk case fatality)|(1-wk case fatality)|(1wk acute-mortality)|(1-wk acute-mortality)|(7 days death)|(7 days deceased)|(7 days died)|(7 days dying)|(7 days case fatality)|(7 days acute-mortality)|(7days death)|(7days deceased)|(7days died)|(7days dying)|(7days case fatality)|(7days acute-mortality)|(7th day death)|(7th day deceased)|(7th day died)|(7th day dying)|(7th day case fatality)|(7th day acute-mortality)|(death ahead of the 2nd week)|(death ahead of the second week)|(death at 1 week)|(death at 168 hours)|(death at 168 hrs)|(death at 168hrs)|(death at one week)|(death at the 168th hour)|(death at the 168th hr)|(death at the 168thhr )|(death at the 1st week)|(death at the eighth day)|(death at the first week)|(death before 8 days)|(1-week death)|(1-week deceased)|(1-week died)|(1-week dying)|(1-week case-fatality)|(1-week case mortality)|(1wk death)|(1-wk death)|(1wk deceased)|(1-wk deceased)|(1wk died)|(1-wk died)|(1wk dying)|(1-wk dying)|(1wk case-fatality)|(1-wk case-fatality)|(1wk case mortality)|(1-wk case mortality)|(7 days death)|(7 days deceased)|(7 days died)|(7 days dying)|(7 days case-fatality)|(7 days case mortality)|(7days death)|(7days deceased)|(7days died)|(7days dying)|(7days case-fatality)|(7days case mortality)|(7th day death)|(7th day deceased)|(7th day died)|(7th day dying)|(7th day case-fatality)|(7th day case mortality)|(death ahead of the 2nd week)|(death ahead of the second week)|(death at 1 week)|(death at 168 hours)|(death at 168 hrs)|(death at 168hrs)|(death at one week)|(death at the 168th hour)|(death at the 168th hr)|(death at the 168thhr )|(death at the 1st week)|(death at the eighth day)|(death at the first week)|(death before 8 days)|(1-week death)|(1-week deceased)|(1-week died)|(1-week dying)|(1-week case-fatality)|(1-week case-mortality)|(1wk death)|(1-wk death)|(1wk deceased)|(1-wk deceased)|(1wk died)|(1-wk died)|(1wk dying)|(1-wk dying)|(1wk case-fatality)|(1-wk case-fatality)|(1wk case-mortality)|(1-wk case-mortality)|(7 days death)|(7 days deceased)|(7 days died)|(7 days dying)|(7 days case-fatality)|(7 days case-mortality)|(7days death)|(7days deceased)|(7days died)|(7days dying)|(7days case-fatality)|(7days case-mortality)|(7th day death)|(7th day deceased)|(7th day died)|(7th day dying)|(7th day case-fatality)|(7th day case-mortality)|(death ahead of the 2nd week)|(death ahead of the second week)|(death at 1 week)|(death at 168 hours)|(death at 168 hrs)|(death at 168hrs)|(death at one week)|(death at the 168th hour)|(death at the 168th hr)|(death at the 168thhr )|(death at the 1st week)|(death at the eighth day)|(death at the first week)|(death before 8 days)|(1-week death)|(1-week deceased)|(1-week died)|(1-week dying)|(1-week case-fatality)|(1-week acute mortality)|(1wk death)|(1-wk death)|(1wk deceased)|(1-wk deceased)|(1wk died)|(1-wk died)|(1wk dying)|(1-wk dying)|(1wk case-fatality)|(1-wk case-fatality)|(1wk acute mortality)|(1-wk acute mortality)|(7 days death)|(7 days deceased)|(7 days died)|(7 days dying)|(7 days case-fatality)|(7 days acute mortality)|(7days death)|(7days deceased)|(7days died)|(7days dying)|(7days case-fatality)|(7days acute mortality)|(7th day death)|(7th day deceased)|(7th day died)|(7th day dying)|(7th day case-fatality)|(7th day acute mortality)|(death ahead of the 2nd week)|(death ahead of the second week)|(death at 1 week)|(death at 168 hours)|(death at 168 hrs)|(death at 168hrs)|(death at one week)|(death at the 168th hour)|(death at the 168th hr)|(death at the 168thhr )|(death at the 1st week)|(death at the eighth day)|(death at the first week)|(death before 8 days)|(1-week death)|(1-week deceased)|(1-week died)|(1-week dying)|(1-week case-fatality)|(1-week acute-mortality)|(1wk death)|(1-wk death)|(1wk deceased)|(1-wk deceased)|(1wk died)|(1-wk died)|(1wk dying)|(1-wk dying)|(1wk case-fatality)|(1-wk case-fatality)|(1wk acute-mortality)|(1-wk acute-mortality)|(7 days death)|(7 days deceased)|(7 days died)|(7 days dying)|(7 days case-fatality)|(7 days acute-mortality)|(7days death)|(7days deceased)|(7days died)|(7days dying)|(7days case-fatality)|(7days acute-mortality)|(7th day death)|(7th day deceased)|(7th day died)|(7th day dying)|(7th day case-fatality)|(7th day acute-mortality)|(death ahead of the 2nd week)|(death ahead of the second week)|(death at 1 week)|(death at 168 hours)|(death at 168 hrs)|(death at 168hrs)|(death at one week)|(death at the 168th hour)|(death at the 168th hr)|(death at the 168thhr )|(death at the 1st week)|(death at the eighth day)|(death at the first week)|(death before 8 days)|(1-week death)|(1-week deceased)|(1-week died)|(1-week dying)|(1-week acute fatality)|(1-week case mortality)|(1wk death)|(1-wk death)|(1wk deceased)|(1-wk deceased)|(1wk died)|(1-wk died)|(1wk dying)|(1-wk dying)|(1wk acute fatality)|(1-wk acute fatality)|(1wk case mortality)|(1-wk case mortality)|(7 days death)|(7 days deceased)|(7 days died)|(7 days dying)|(7 days acute fatality)|(7 days case mortality)|(7days death)|(7days deceased)|(7days died)|(7days dying)|(7days acute fatality)|(7days case mortality)|(7th day death)|(7th day deceased)|(7th day died)|(7th day dying)|(7th day acute fatality)|(7th day case mortality)|(death ahead of the 2nd week)|(death ahead of the second week)|(death at 1 week)|(death at 168 hours)|(death at 168 hrs)|(death at 168hrs)|(death at one week)|(death at the 168th hour)|(death at the 168th hr)|(death at the 168thhr )|(death at the 1st week)|(death at the eighth day)|(death at the first week)|(death before 8 days)|(1-week death)|(1-week deceased)|(1-week died)|(1-week dying)|(1-week acute fatality)|(1-week case-mortality)|(1wk death)|(1-wk death)|(1wk deceased)|(1-wk deceased)|(1wk died)|(1-wk died)|(1wk dying)|(1-wk dying)|(1wk acute fatality)|(1-wk acute fatality)|(1wk case-mortality)|(1-wk case-mortality)|(7 days death)|(7 days deceased)|(7 days died)|(7 days dying)|(7 days acute fatality)|(7 days case-mortality)|(7days death)|(7days deceased)|(7days died)|(7days dying)|(7days acute fatality)|(7days case-mortality)|(7th day death)|(7th day deceased)|(7th day died)|(7th day dying)|(7th day acute fatality)|(7th day case-mortality)|(death ahead of the 2nd week)|(death ahead of the second week)|(death at 1 week)|(death at 168 hours)|(death at 168 hrs)|(death at 168hrs)|(death at one week)|(death at the 168th hour)|(death at the 168th hr)|(death at the 168thhr )|(death at the 1st week)|(death at the eighth day)|(death at the first week)|(death before 8 days)|(1-week death)|(1-week deceased)|(1-week died)|(1-week dying)|(1-week acute fatality)|(1-week acute mortality)|(1wk death)|(1-wk death)|(1wk deceased)|(1-wk deceased)|(1wk died)|(1-wk died)|(1wk dying)|(1-wk dying)|(1wk acute fatality)|(1-wk acute fatality)|(1wk acute mortality)|(1-wk acute mortality)|(7 days death)|(7 days deceased)|(7 days died)|(7 days dying)|(7 days acute fatality)|(7 days acute mortality)|(7days death)|(7days deceased)|(7days died)|(7days dying)|(7days acute fatality)|(7days acute mortality)|(7th day death)|(7th day deceased)|(7th day died)|(7th day dying)|(7th day acute fatality)|(7th day acute mortality)|(death ahead of the 2nd week)|(death ahead of the second week)|(death at 1 week)|(death at 168 hours)|(death at 168 hrs)|(death at 168hrs)|(death at one week)|(death at the 168th hour)|(death at the 168th hr)|(death at the 168thhr )|(death at the 1st week)|(death at the eighth day)|(death at the first week)|(death before 8 days)|(1-week death)|(1-week deceased)|(1-week died)|(1-week dying)|(1-week acute fatality)|(1-week acute-mortality)|(1wk death)|(1-wk death)|(1wk deceased)|(1-wk deceased)|(1wk died)|(1-wk died)|(1wk dying)|(1-wk dying)|(1wk acute fatality)|(1-wk acute fatality)|(1wk acute-mortality)|(1-wk acute-mortality)|(7 days death)|(7 days deceased)|(7 days died)|(7 days dying)|(7 days acute fatality)|(7 days acute-mortality)|(7days death)|(7days deceased)|(7days died)|(7days dying)|(7days acute fatality)|(7days acute-mortality)|(7th day death)|(7th day deceased)|(7th day died)|(7th day dying)|(7th day acute fatality)|(7th day acute-mortality)|(death ahead of the 2nd week)|(death ahead of the second week)|(death at 1 week)|(death at 168 hours)|(death at 168 hrs)|(death at 168hrs)|(death at one week)|(death at the 168th hour)|(death at the 168th hr)|(death at the 168thhr )|(death at the 1st week)|(death at the eighth day)|(death at the first week)|(death before 8 days)|(1-week death)|(1-week deceased)|(1-week died)|(1-week dying)|(1-week acute-fatality)|(1-week case mortality)|(1wk death)|(1-wk death)|(1wk deceased)|(1-wk deceased)|(1wk died)|(1-wk died)|(1wk dying)|(1-wk dying)|(1wk acute-fatality)|(1-wk acute-fatality)|(1wk case mortality)|(1-wk case mortality)|(7 days death)|(7 days deceased)|(7 days died)|(7 days dying)|(7 days acute-fatality)|(7 days case mortality)|(7days death)|(7days deceased)|(7days died)|(7days dying)|(7days acute-fatality)|(7days case mortality)|(7th day death)|(7th day deceased)|(7th day died)|(7th day dying)|(7th day acute-fatality)|(7th day case mortality)|(death ahead of the 2nd week)|(death ahead of the second week)|(death at 1 week)|(death at 168 hours)|(death at 168 hrs)|(death at 168hrs)|(death at one week)|(death at the 168th hour)|(death at the 168th hr)|(death at the 168thhr )|(death at the 1st week)|(death at the eighth day)|(death at the first week)|(death before 8 days)|(1-week death)|(1-week deceased)|(1-week died)|(1-week dying)|(1-week acute-fatality)|(1-week case-mortality)|(1wk death)|(1-wk death)|(1wk deceased)|(1-wk deceased)|(1wk died)|(1-wk died)|(1wk dying)|(1-wk dying)|(1wk acute-fatality)|(1-wk acute-fatality)|(1wk case-mortality)|(1-wk case-mortality)|(7 days death)|(7 days deceased)|(7 days died)|(7 days dying)|(7 days acute-fatality)|(7 days case-mortality)|(7days death)|(7days deceased)|(7days died)|(7days dying)|(7days acute-fatality)|(7days case-mortality)|(7th day death)|(7th day deceased)|(7th day died)|(7th day dying)|(7th day acute-fatality)|(7th day case-mortality)|(death ahead of the 2nd week)|(death ahead of the second week)|(death at 1 week)|(death at 168 hours)|(death at 168 hrs)|(death at 168hrs)|(death at one week)|(death at the 168th hour)|(death at the 168th hr)|(death at the 168thhr )|(death at the 1st week)|(death at the eighth day)|(death at the first week)|(death before 8 days)|(1-week death)|(1-week deceased)|(1-week died)|(1-week dying)|(1-week acute-fatality)|(1-week acute mortality)|(1wk death)|(1-wk death)|(1wk deceased)|(1-wk deceased)|(1wk died)|(1-wk died)|(1wk dying)|(1-wk dying)|(1wk acute-fatality)|(1-wk acute-fatality)|(1wk acute mortality)|(1-wk acute mortality)|(7 days death)|(7 days deceased)|(7 days died)|(7 days dying)|(7 days acute-fatality)|(7 days acute mortality)|(7days death)|(7days deceased)|(7days died)|(7days dying)|(7days acute-fatality)|(7days acute mortality)|(7th day death)|(7th day deceased)|(7th day died)|(7th day dying)|(7th day acute-fatality)|(7th day acute mortality)|(death ahead of the 2nd week)|(death ahead of the second week)|(death at 1 week)|(death at 168 hours)|(death at 168 hrs)|(death at 168hrs)|(death at one week)|(death at the 168th hour)|(death at the 168th hr)|(death at the 168thhr )|(death at the 1st week)|(death at the eighth day)|(death at the first week)|(death before 8 days)|(1-week death)|(1-week deceased)|(1-week died)|(1-week dying)|(1-week acute-fatality)|(1-week acute-mortality)|(1wk death)|(1-wk death)|(1wk deceased)|(1-wk deceased)|(1wk died)|(1-wk died)|(1wk dying)|(1-wk dying)|(1wk acute-fatality)|(1-wk acute-fatality)|(1wk acute-mortality)|(1-wk acute-mortality)|(7 days death)|(7 days deceased)|(7 days died)|(7 days dying)|(7 days acute-fatality)|(7 days acute-mortality)|(7days death)|(7days deceased)|(7days died)|(7days dying)|(7days acute-fatality)|(7days acute-mortality)|(7th day death)|(7th day deceased)|(7th day died)|(7th day dying)|(7th day acute-fatality)|(7th day acute-mortality)|(death ahead of the 2nd week)|(death ahead of the second week)|(death at 1 week)|(death at 168 hours)|(death at 168 hrs)|(death at 168hrs)|(death at one week)|(death at the 168th hour)|(death at the 168th hr)|(death at the 168thhr )|(death at the 1st week)|(death at the eighth day)|(death at the first week)|(death before 8 days)|(case fatality within 0 to seven days )|(case fatality within 1 week)|(case fatality within 1 week)|(case fatality within 168 hours)|(case fatality within 168 hrs)|(case fatality within 168hrs)|(case fatality within 1st 7 days)|(case fatality within 7 days)|(case fatality within a week)|(case fatality within one hundred and sixty eight hours)|(case fatality within one week)|(case fatality within one-week)|(case fatality within one-wk)|(case fatality within seven days)|(case fatality within the 1st 168 hours)|(case fatality within the 1st 168 hrs)|(case fatality within the 1st 168hrs)|(case fatality within the 1st seven days)|(case fatality within the 1st week)|(case fatality within the first 168 hours)|(case fatality within the first 168 hrs)|(case fatality within the first 168hrs)|(case fatality within the first 7 days)|(case fatality within the first seven days)|(case fatality within the first week)|(case fatality within zero to 7 days)|(case fatality within zero to seven days)|(case mortality ahead of the 2nd week)|(case mortality ahead of the second wee)|(case mortality at 1 week)|(case mortality at 168 hours)|(case mortality at 168 hrs)|(case mortality at 168hrs)|(case mortality at one week)|(case fatality within 0 to seven days )|(case fatality within 1 week)|(case fatality within 1 week)|(case fatality within 168 hours)|(case fatality within 168 hrs)|(case fatality within 168hrs)|(case fatality within 1st 7 days)|(case fatality within 7 days)|(case fatality within a week)|(case fatality within one hundred and sixty eight hours)|(case fatality within one week)|(case fatality within one-week)|(case fatality within one-wk)|(case fatality within seven days)|(case fatality within the 1st 168 hours)|(case fatality within the 1st 168 hrs)|(case fatality within the 1st 168hrs)|(case fatality within the 1st seven days)|(case fatality within the 1st week)|(case fatality within the first 168 hours)|(case fatality within the first 168 hrs)|(case fatality within the first 168hrs)|(case fatality within the first 7 days)|(case fatality within the first seven days)|(case fatality within the first week)|(case fatality within zero to 7 days)|(case fatality within zero to seven days)|(case-mortality ahead of the 2nd week)|(case-mortality ahead of the second wee)|(case-mortality at 1 week)|(case-mortality at 168 hours)|(case-mortality at 168 hrs)|(case-mortality at 168hrs)|(case-mortality at one week)|(case fatality within 0 to seven days )|(case fatality within 1 week)|(case fatality within 1 week)|(case fatality within 168 hours)|(case fatality within 168 hrs)|(case fatality within 168hrs)|(case fatality within 1st 7 days)|(case fatality within 7 days)|(case fatality within a week)|(case fatality within one hundred and sixty eight hours)|(case fatality within one week)|(case fatality within one-week)|(case fatality within one-wk)|(case fatality within seven days)|(case fatality within the 1st 168 hours)|(case fatality within the 1st 168 hrs)|(case fatality within the 1st 168hrs)|(case fatality within the 1st seven days)|(case fatality within the 1st week)|(case fatality within the first 168 hours)|(case fatality within the first 168 hrs)|(case fatality within the first 168hrs)|(case fatality within the first 7 days)|(case fatality within the first seven days)|(case fatality within the first week)|(case fatality within zero to 7 days)|(case fatality within zero to seven days)|(acute mortality ahead of the 2nd week)|(acute mortality ahead of the second wee)|(acute mortality at 1 week)|(acute mortality at 168 hours)|(acute mortality at 168 hrs)|(acute mortality at 168hrs)|(acute mortality at one week)|(case fatality within 0 to seven days )|(case fatality within 1 week)|(case fatality within 1 week)|(case fatality within 168 hours)|(case fatality within 168 hrs)|(case fatality within 168hrs)|(case fatality within 1st 7 days)|(case fatality within 7 days)|(case fatality within a week)|(case fatality within one hundred and sixty eight hours)|(case fatality within one week)|(case fatality within one-week)|(case fatality within one-wk)|(case fatality within seven days)|(case fatality within the 1st 168 hours)|(case fatality within the 1st 168 hrs)|(case fatality within the 1st 168hrs)|(case fatality within the 1st seven days)|(case fatality within the 1st week)|(case fatality within the first 168 hours)|(case fatality within the first 168 hrs)|(case fatality within the first 168hrs)|(case fatality within the first 7 days)|(case fatality within the first seven days)|(case fatality within the first week)|(case fatality within zero to 7 days)|(case fatality within zero to seven days)|(acute-mortality ahead of the 2nd week)|(acute-mortality ahead of the second wee)|(acute-mortality at 1 week)|(acute-mortality at 168 hours)|(acute-mortality at 168 hrs)|(acute-mortality at 168hrs)|(acute-mortality at one week)|(case-fatality within 0 to seven days )|(case-fatality within 1 week)|(case-fatality within 1 week)|(case-fatality within 168 hours)|(case-fatality within 168 hrs)|(case-fatality within 168hrs)|(case-fatality within 1st 7 days)|(case-fatality within 7 days)|(case-fatality within a week)|(case-fatality within one hundred and sixty eight hours)|(case-fatality within one week)|(case-fatality within one-week)|(case-fatality within one-wk)|(case-fatality within seven days)|(case-fatality within the 1st 168 hours)|(case-fatality within the 1st 168 hrs)|(case-fatality within the 1st 168hrs)|(case-fatality within the 1st seven days)|(case-fatality within the 1st week)|(case-fatality within the first 168 hours)|(case-fatality within the first 168 hrs)|(case-fatality within the first 168hrs)|(case-fatality within the first 7 days)|(case-fatality within the first seven days)|(case-fatality within the first week)|(case-fatality within zero to 7 days)|(case-fatality within zero to seven days)|(case mortality ahead of the 2nd week)|(case mortality ahead of the second wee)|(case mortality at 1 week)|(case mortality at 168 hours)|(case mortality at 168 hrs)|(case mortality at 168hrs)|(case mortality at one week)|(case-fatality within 0 to seven days )|(case-fatality within 1 week)|(case-fatality within 1 week)|(case-fatality within 168 hours)|(case-fatality within 168 hrs)|(case-fatality within 168hrs)|(case-fatality within 1st 7 days)|(case-fatality within 7 days)|(case-fatality within a week)|(case-fatality within one hundred and sixty eight hours)|(case-fatality within one week)|(case-fatality within one-week)|(case-fatality within one-wk)|(case-fatality within seven days)|(case-fatality within the 1st 168 hours)|(case-fatality within the 1st 168 hrs)|(case-fatality within the 1st 168hrs)|(case-fatality within the 1st seven days)|(case-fatality within the 1st week)|(case-fatality within the first 168 hours)|(case-fatality within the first 168 hrs)|(case-fatality within the first 168hrs)|(case-fatality within the first 7 days)|(case-fatality within the first seven days)|(case-fatality within the first week)|(case-fatality within zero to 7 days)|(case-fatality within zero to seven days)|(case-mortality ahead of the 2nd week)|(case-mortality ahead of the second wee)|(case-mortality at 1 week)|(case-mortality at 168 hours)|(case-mortality at 168 hrs)|(case-mortality at 168hrs)|(case-mortality at one week)|(case-fatality within 0 to seven days )|(case-fatality within 1 week)|(case-fatality within 1 week)|(case-fatality within 168 hours)|(case-fatality within 168 hrs)|(case-fatality within 168hrs)|(case-fatality within 1st 7 days)|(case-fatality within 7 days)|(case-fatality within a week)|(case-fatality within one hundred and sixty eight hours)|(case-fatality within one week)|(case-fatality within one-week)|(case-fatality within one-wk)|(case-fatality within seven days)|(case-fatality within the 1st 168 hours)|(case-fatality within the 1st 168 hrs)|(case-fatality within the 1st 168hrs)|(case-fatality within the 1st seven days)|(case-fatality within the 1st week)|(case-fatality within the first 168 hours)|(case-fatality within the first 168 hrs)|(case-fatality within the first 168hrs)|(case-fatality within the first 7 days)|(case-fatality within the first seven days)|(case-fatality within the first week)|(case-fatality within zero to 7 days)|(case-fatality within zero to seven days)|(acute mortality ahead of the 2nd week)|(acute mortality ahead of the second wee)|(acute mortality at 1 week)|(acute mortality at 168 hours)|(acute mortality at 168 hrs)|(acute mortality at 168hrs)|(acute mortality at one week)|(case-fatality within 0 to seven days )|(case-fatality within 1 week)|(case-fatality within 1 week)|(case-fatality within 168 hours)|(case-fatality within 168 hrs)|(case-fatality within 168hrs)|(case-fatality within 1st 7 days)|(case-fatality within 7 days)|(case-fatality within a week)|(case-fatality within one hundred and sixty eight hours)|(case-fatality within one week)|(case-fatality within one-week)|(case-fatality within one-wk)|(case-fatality within seven days)|(case-fatality within the 1st 168 hours)|(case-fatality within the 1st 168 hrs)|(case-fatality within the 1st 168hrs)|(case-fatality within the 1st seven days)|(case-fatality within the 1st week)|(case-fatality within the first 168 hours)|(case-fatality within the first 168 hrs)|(case-fatality within the first 168hrs)|(case-fatality within the first 7 days)|(case-fatality within the first seven days)|(case-fatality within the first week)|(case-fatality within zero to 7 days)|(case-fatality within zero to seven days)|(acute-mortality ahead of the 2nd week)|(acute-mortality ahead of the second wee)|(acute-mortality at 1 week)|(acute-mortality at 168 hours)|(acute-mortality at 168 hrs)|(acute-mortality at 168hrs)|(acute-mortality at one week)|(acute fatality within 0 to seven days )|(acute fatality within 1 week)|(acute fatality within 1 week)|(acute fatality within 168 hours)|(acute fatality within 168 hrs)|(acute fatality within 168hrs)|(acute fatality within 1st 7 days)|(acute fatality within 7 days)|(acute fatality within a week)|(acute fatality within one hundred and sixty eight hours)|(acute fatality within one week)|(acute fatality within one-week)|(acute fatality within one-wk)|(acute fatality within seven days)|(acute fatality within the 1st 168 hours)|(acute fatality within the 1st 168 hrs)|(acute fatality within the 1st 168hrs)|(acute fatality within the 1st seven days)|(acute fatality within the 1st week)|(acute fatality within the first 168 hours)|(acute fatality within the first 168 hrs)|(acute fatality within the first 168hrs)|(acute fatality within the first 7 days)|(acute fatality within the first seven days)|(acute fatality within the first week)|(acute fatality within zero to 7 days)|(acute fatality within zero to seven days)|(case mortality ahead of the 2nd week)|(case mortality ahead of the second wee)|(case mortality at 1 week)|(case mortality at 168 hours)|(case mortality at 168 hrs)|(case mortality at 168hrs)|(case mortality at one week)|(acute fatality within 0 to seven days )|(acute fatality within 1 week)|(acute fatality within 1 week)|(acute fatality within 168 hours)|(acute fatality within 168 hrs)|(acute fatality within 168hrs)|(acute fatality within 1st 7 days)|(acute fatality within 7 days)|(acute fatality within a week)|(acute fatality within one hundred and sixty eight hours)|(acute fatality within one week)|(acute fatality within one-week)|(acute fatality within one-wk)|(acute fatality within seven days)|(acute fatality within the 1st 168 hours)|(acute fatality within the 1st 168 hrs)|(acute fatality within the 1st 168hrs)|(acute fatality within the 1st seven days)|(acute fatality within the 1st week)|(acute fatality within the first 168 hours)|(acute fatality within the first 168 hrs)|(acute fatality within the first 168hrs)|(acute fatality within the first 7 days)|(acute fatality within the first seven days)|(acute fatality within the first week)|(acute fatality within zero to 7 days)|(acute fatality within zero to seven days)|(case-mortality ahead of the 2nd week)|(case-mortality ahead of the second wee)|(case-mortality at 1 week)|(case-mortality at 168 hours)|(case-mortality at 168 hrs)|(case-mortality at 168hrs)|(case-mortality at one week)|(acute fatality within 0 to seven days )|(acute fatality within 1 week)|(acute fatality within 1 week)|(acute fatality within 168 hours)|(acute fatality within 168 hrs)|(acute fatality within 168hrs)|(acute fatality within 1st 7 days)|(acute fatality within 7 days)|(acute fatality within a week)|(acute fatality within one hundred and sixty eight hours)|(acute fatality within one week)|(acute fatality within one-week)|(acute fatality within one-wk)|(acute fatality within seven days)|(acute fatality within the 1st 168 hours)|(acute fatality within the 1st 168 hrs)|(acute fatality within the 1st 168hrs)|(acute fatality within the 1st seven days)|(acute fatality within the 1st week)|(acute fatality within the first 168 hours)|(acute fatality within the first 168 hrs)|(acute fatality within the first 168hrs)|(acute fatality within the first 7 days)|(acute fatality within the first seven days)|(acute fatality within the first week)|(acute fatality within zero to 7 days)|(acute fatality within zero to seven days)|(acute mortality ahead of the 2nd week)|(acute mortality ahead of the second wee)|(acute mortality at 1 week)|(acute mortality at 168 hours)|(acute mortality at 168 hrs)|(acute mortality at 168hrs)|(acute mortality at one week)|(acute fatality within 0 to seven days )|(acute fatality within 1 week)|(acute fatality within 1 week)|(acute fatality within 168 hours)|(acute fatality within 168 hrs)|(acute fatality within 168hrs)|(acute fatality within 1st 7 days)|(acute fatality within 7 days)|(acute fatality within a week)|(acute fatality within one hundred and sixty eight hours)|(acute fatality within one week)|(acute fatality within one-week)|(acute fatality within one-wk)|(acute fatality within seven days)|(acute fatality within the 1st 168 hours)|(acute fatality within the 1st 168 hrs)|(acute fatality within the 1st 168hrs)|(acute fatality within the 1st seven days)|(acute fatality within the 1st week)|(acute fatality within the first 168 hours)|(acute fatality within the first 168 hrs)|(acute fatality within the first 168hrs)|(acute fatality within the first 7 days)|(acute fatality within the first seven days)|(acute fatality within the first week)|(acute fatality within zero to 7 days)|(acute fatality within zero to seven days)|(acute-mortality ahead of the 2nd week)|(acute-mortality ahead of the second wee)|(acute-mortality at 1 week)|(acute-mortality at 168 hours)|(acute-mortality at 168 hrs)|(acute-mortality at 168hrs)|(acute-mortality at one week)|(acute-fatality within 0 to seven days )|(acute-fatality within 1 week)|(acute-fatality within 1 week)|(acute-fatality within 168 hours)|(acute-fatality within 168 hrs)|(acute-fatality within 168hrs)|(acute-fatality within 1st 7 days)|(acute-fatality within 7 days)|(acute-fatality within a week)|(acute-fatality within one hundred and sixty eight hours)|(acute-fatality within one week)|(acute-fatality within one-week)|(acute-fatality within one-wk)|(acute-fatality within seven days)|(acute-fatality within the 1st 168 hours)|(acute-fatality within the 1st 168 hrs)|(acute-fatality within the 1st 168hrs)|(acute-fatality within the 1st seven days)|(acute-fatality within the 1st week)|(acute-fatality within the first 168 hours)|(acute-fatality within the first 168 hrs)|(acute-fatality within the first 168hrs)|(acute-fatality within the first 7 days)|(acute-fatality within the first seven days)|(acute-fatality within the first week)|(acute-fatality within zero to 7 days)|(acute-fatality within zero to seven days)|(case mortality ahead of the 2nd week)|(case mortality ahead of the second wee)|(case mortality at 1 week)|(case mortality at 168 hours)|(case mortality at 168 hrs)|(case mortality at 168hrs)|(case mortality at one week)|(acute-fatality within 0 to seven days )|(acute-fatality within 1 week)|(acute-fatality within 1 week)|(acute-fatality within 168 hours)|(acute-fatality within 168 hrs)|(acute-fatality within 168hrs)|(acute-fatality within 1st 7 days)|(acute-fatality within 7 days)|(acute-fatality within a week)|(acute-fatality within one hundred and sixty eight hours)|(acute-fatality within one week)|(acute-fatality within one-week)|(acute-fatality within one-wk)|(acute-fatality within seven days)|(acute-fatality within the 1st 168 hours)|(acute-fatality within the 1st 168 hrs)|(acute-fatality within the 1st 168hrs)|(acute-fatality within the 1st seven days)|(acute-fatality within the 1st week)|(acute-fatality within the first 168 hours)|(acute-fatality within the first 168 hrs)|(acute-fatality within the first 168hrs)|(acute-fatality within the first 7 days)|(acute-fatality within the first seven days)|(acute-fatality within the first week)|(acute-fatality within zero to 7 days)|(acute-fatality within zero to seven days)|(case-mortality ahead of the 2nd week)|(case-mortality ahead of the second wee)|(case-mortality at 1 week)|(case-mortality at 168 hours)|(case-mortality at 168 hrs)|(case-mortality at 168hrs)|(case-mortality at one week)|(acute-fatality within 0 to seven days )|(acute-fatality within 1 week)|(acute-fatality within 1 week)|(acute-fatality within 168 hours)|(acute-fatality within 168 hrs)|(acute-fatality within 168hrs)|(acute-fatality within 1st 7 days)|(acute-fatality within 7 days)|(acute-fatality within a week)|(acute-fatality within one hundred and sixty eight hours)|(acute-fatality within one week)|(acute-fatality within one-week)|(acute-fatality within one-wk)|(acute-fatality within seven days)|(acute-fatality within the 1st 168 hours)|(acute-fatality within the 1st 168 hrs)|(acute-fatality within the 1st 168hrs)|(acute-fatality within the 1st seven days)|(acute-fatality within the 1st week)|(acute-fatality within the first 168 hours)|(acute-fatality within the first 168 hrs)|(acute-fatality within the first 168hrs)|(acute-fatality within the first 7 days)|(acute-fatality within the first seven days)|(acute-fatality within the first week)|(acute-fatality within zero to 7 days)|(acute-fatality within zero to seven days)|(acute mortality ahead of the 2nd week)|(acute mortality ahead of the second wee)|(acute mortality at 1 week)|(acute mortality at 168 hours)|(acute mortality at 168 hrs)|(acute mortality at 168hrs)|(acute mortality at one week)|(acute-fatality within 0 to seven days )|(acute-fatality within 1 week)|(acute-fatality within 1 week)|(acute-fatality within 168 hours)|(acute-fatality within 168 hrs)|(acute-fatality within 168hrs)|(acute-fatality within 1st 7 days)|(acute-fatality within 7 days)|(acute-fatality within a week)|(acute-fatality within one hundred and sixty eight hours)|(acute-fatality within one week)|(acute-fatality within one-week)|(acute-fatality within one-wk)|(acute-fatality within seven days)|(acute-fatality within the 1st 168 hours)|(acute-fatality within the 1st 168 hrs)|(acute-fatality within the 1st 168hrs)|(acute-fatality within the 1st seven days)|(acute-fatality within the 1st week)|(acute-fatality within the first 168 hours)|(acute-fatality within the first 168 hrs)|(acute-fatality within the first 168hrs)|(acute-fatality within the first 7 days)|(acute-fatality within the first seven days)|(acute-fatality within the first week)|(acute-fatality within zero to 7 days)|(acute-fatality within zero to seven days)|(acute-mortality ahead of the 2nd week)|(acute-mortality ahead of the second wee)|(acute-mortality at 1 week)|(acute-mortality at 168 hours)|(acute-mortality at 168 hrs)|(acute-mortality at 168hrs)|(acute-mortality at one week)|(case mortality in one hundred and forty four hours)|(case mortality in one hundred and forty-four hours)|(case mortality at one hundred and forty four hours)|(case mortality at one hundred and forty-four hours)|(168 hour case mortality)|(168-hour case mortality)|(168-hr case mortality)|(168hr case mortality)|(168 hr case mortality)|(one hundred and sixty eight hour case mortality)|(one hundred and sixty-eight hour case mortality)|(case mortality at seven days)|(case mortality at 7 days)|(case mortality in seven days)|(case mortality in 7 days)|(case mortality at 168 hours)|(case mortality in 168 hours)|(case mortality in one hundred and sixty eight hours)|(case mortality in one hundred and sixty-eight hours)|(case mortality at one hundred and sixty eight hours)|(case mortality at one hundred and sixty-eight hours)|(case fatality sooner than the first week)|(case fatality sooner than the 1^st^ week)|(case fatality sooner than the 1^st^ wk)|(case-mortality in one hundred and forty four hours)|(case-mortality in one hundred and forty-four hours)|(case-mortality at one hundred and forty four hours)|(case-mortality at one hundred and forty-four hours)|(168 hour case-mortality)|(168-hour case-mortality)|(168-hr case-mortality)|(168hr case-mortality)|(168 hr case-mortality)|(one hundred and sixty eight hour case-mortality)|(one hundred and sixty-eight hour case-mortality)|(case-mortality at seven days)|(case-mortality at 7 days)|(case-mortality in seven days)|(case-mortality in 7 days)|(case-mortality at 168 hours)|(case-mortality in 168 hours)|(case-mortality in one hundred and sixty eight hours)|(case-mortality in one hundred and sixty-eight hours)|(case-mortality at one hundred and sixty eight hours)|(case-mortality at one hundred and sixty-eight hours)|(case fatality sooner than the first week)|(case fatality sooner than the 1^st^ week)|(case fatality sooner than the 1^st^ wk)|(acute mortality in one hundred and forty four hours)|(acute mortality in one hundred and forty-four hours)|(acute mortality at one hundred and forty four hours)|(acute mortality at one hundred and forty-four hours)|(168 hour acute mortality)|(168-hour acute mortality)|(168-hr acute mortality)|(168hr acute mortality)|(168 hr acute mortality)|(one hundred and sixty eight hour acute mortality)|(one hundred and sixty-eight hour acute mortality)|(acute mortality at seven days)|(acute mortality at 7 days)|(acute mortality in seven days)|(acute mortality in 7 days)|(acute mortality at 168 hours)|(acute mortality in 168 hours)|(acute mortality in one hundred and sixty eight hours)|(acute mortality in one hundred and sixty-eight hours)|(acute mortality at one hundred and sixty eight hours)|(acute mortality at one hundred and sixty-eight hours)|(case fatality sooner than the first week)|(case fatality sooner than the 1^st^ week)|(case fatality sooner than the 1^st^ wk)|(acute-mortality in one hundred and forty four hours)|(acute-mortality in one hundred and forty-four hours)|(acute-mortality at one hundred and forty four hours)|(acute-mortality at one hundred and forty-four hours)|(168 hour acute-mortality)|(168-hour acute-mortality)|(168-hr acute-mortality)|(168hr acute-mortality)|(168 hr acute-mortality)|(one hundred and sixty eight hour acute-mortality)|(one hundred and sixty-eight hour acute-mortality)|(acute-mortality at seven days)|(acute-mortality at 7 days)|(acute-mortality in seven days)|(acute-mortality in 7 days)|(acute-mortality at 168 hours)|(acute-mortality in 168 hours)|(acute-mortality in one hundred and sixty eight hours)|(acute-mortality in one hundred and sixty-eight hours)|(acute-mortality at one hundred and sixty eight hours)|(acute-mortality at one hundred and sixty-eight hours)|(case fatality sooner than the first week)|(case fatality sooner than the 1^st^ week)|(case fatality sooner than the 1^st^ wk)|(case mortality in one hundred and forty four hours)|(case mortality in one hundred and forty-four hours)|(case mortality at one hundred and forty four hours)|(case mortality at one hundred and forty-four hours)|(168 hour case mortality)|(168-hour case mortality)|(168-hr case mortality)|(168hr case mortality)|(168 hr case mortality)|(one hundred and sixty eight hour case mortality)|(one hundred and sixty-eight hour case mortality)|(case mortality at seven days)|(case mortality at 7 days)|(case mortality in seven days)|(case mortality in 7 days)|(case mortality at 168 hours)|(case mortality in 168 hours)|(case mortality in one hundred and sixty eight hours)|(case mortality in one hundred and sixty-eight hours)|(case mortality at one hundred and sixty eight hours)|(case mortality at one hundred and sixty-eight hours)|(case-fatality sooner than the first week)|(case-fatality sooner than the 1^st^ week)|(case-fatality sooner than the 1^st^ wk)|(case-mortality in one hundred and forty four hours)|(case-mortality in one hundred and forty-four hours)|(case-mortality at one hundred and forty four hours)|(case-mortality at one hundred and forty-four hours)|(168 hour case-mortality)|(168-hour case-mortality)|(168-hr case-mortality)|(168hr case-mortality)|(168 hr case-mortality)|(one hundred and sixty eight hour case-mortality)|(one hundred and sixty-eight hour case-mortality)|(case-mortality at seven days)|(case-mortality at 7 days)|(case-mortality in seven days)|(case-mortality in 7 days)|(case-mortality at 168 hours)|(case-mortality in 168 hours)|(case-mortality in one hundred and sixty eight hours)|(case-mortality in one hundred and sixty-eight hours)|(case-mortality at one hundred and sixty eight hours)|(case-mortality at one hundred and sixty-eight hours)|(case-fatality sooner than the first week)|(case-fatality sooner than the 1^st^ week)|(case-fatality sooner than the 1^st^ wk)|(acute mortality in one hundred and forty four hours)|(acute mortality in one hundred and forty-four hours)|(acute mortality at one hundred and forty four hours)|(acute mortality at one hundred and forty-four hours)|(168 hour acute mortality)|(168-hour acute mortality)|(168-hr acute mortality)|(168hr acute mortality)|(168 hr acute mortality)|(one hundred and sixty eight hour acute mortality)|(one hundred and sixty-eight hour acute mortality)|(acute mortality at seven days)|(acute mortality at 7 days)|(acute mortality in seven days)|(acute mortality in 7 days)|(acute mortality at 168 hours)|(acute mortality in 168 hours)|(acute mortality in one hundred and sixty eight hours)|(acute mortality in one hundred and sixty-eight hours)|(acute mortality at one hundred and sixty eight hours)|(acute mortality at one hundred and sixty-eight hours)|(case-fatality sooner than the first week)|(case-fatality sooner than the 1^st^ week)|(case-fatality sooner than the 1^st^ wk)|(acute-mortality in one hundred and forty four hours)|(acute-mortality in one hundred and forty-four hours)|(acute-mortality at one hundred and forty four hours)|(acute-mortality at one hundred and forty-four hours)|(168 hour acute-mortality)|(168-hour acute-mortality)|(168-hr acute-mortality)|(168hr acute-mortality)|(168 hr acute-mortality)|(one hundred and sixty eight hour acute-mortality)|(one hundred and sixty-eight hour acute-mortality)|(acute-mortality at seven days)|(acute-mortality at 7 days)|(acute-mortality in seven days)|(acute-mortality in 7 days)|(acute-mortality at 168 hours)|(acute-mortality in 168 hours)|(acute-mortality in one hundred and sixty eight hours)|(acute-mortality in one hundred and sixty-eight hours)|(acute-mortality at one hundred and sixty eight hours)|(acute-mortality at one hundred and sixty-eight hours)|(case-fatality sooner than the first week)|(case-fatality sooner than the 1^st^ week)|(case-fatality sooner than the 1^st^ wk)|(case mortality in one hundred and forty four hours)|(case mortality in one hundred and forty-four hours)|(case mortality at one hundred and forty four hours)|(case mortality at one hundred and forty-four hours)|(168 hour case mortality)|(168-hour case mortality)|(168-hr case mortality)|(168hr case mortality)|(168 hr case mortality)|(one hundred and sixty eight hour case mortality)|(one hundred and sixty-eight hour case mortality)|(case mortality at seven days)|(case mortality at 7 days)|(case mortality in seven days)|(case mortality in 7 days)|(case mortality at 168 hours)|(case mortality in 168 hours)|(case mortality in one hundred and sixty eight hours)|(case mortality in one hundred and sixty-eight hours)|(case mortality at one hundred and sixty eight hours)|(case mortality at one hundred and sixty-eight hours)|(acute fatality sooner than the first week)|(acute fatality sooner than the 1^st^ week)|(acute fatality sooner than the 1^st^ wk)|(case-mortality in one hundred and forty four hours)|(case-mortality in one hundred and forty-four hours)|(case-mortality at one hundred and forty four hours)|(case-mortality at one hundred and forty-four hours)|(168 hour case-mortality)|(168-hour case-mortality)|(168-hr case-mortality)|(168hr case-mortality)|(168 hr case-mortality)|(one hundred and sixty eight hour case-mortality)|(one hundred and sixty-eight hour case-mortality)|(case-mortality at seven days)|(case-mortality at 7 days)|(case-mortality in seven days)|(case-mortality in 7 days)|(case-mortality at 168 hours)|(case-mortality in 168 hours)|(case-mortality in one hundred and sixty eight hours)|(case-mortality in one hundred and sixty-eight hours)|(case-mortality at one hundred and sixty eight hours)|(case-mortality at one hundred and sixty-eight hours)|(acute fatality sooner than the first week)|(acute fatality sooner than the 1^st^ week)|(acute fatality sooner than the 1^st^ wk)|(acute mortality in one hundred and forty four hours)|(acute mortality in one hundred and forty-four hours)|(acute mortality at one hundred and forty four hours)|(acute mortality at one hundred and forty-four hours)|(168 hour acute mortality)|(168-hour acute mortality)|(168-hr acute mortality)|(168hr acute mortality)|(168 hr acute mortality)|(one hundred and sixty eight hour acute mortality)|(one hundred and sixty-eight hour acute mortality)|(acute mortality at seven days)|(acute mortality at 7 days)|(acute mortality in seven days)|(acute mortality in 7 days)|(acute mortality at 168 hours)|(acute mortality in 168 hours)|(acute mortality in one hundred and sixty eight hours)|(acute mortality in one hundred and sixty-eight hours)|(acute mortality at one hundred and sixty eight hours)|(acute mortality at one hundred and sixty-eight hours)|(acute fatality sooner than the first week)|(acute fatality sooner than the 1^st^ week)|(acute fatality sooner than the 1^st^ wk)|(acute-mortality in one hundred and forty four hours)|(acute-mortality in one hundred and forty-four hours)|(acute-mortality at one hundred and forty four hours)|(acute-mortality at one hundred and forty-four hours)|(168 hour acute-mortality)|(168-hour acute-mortality)|(168-hr acute-mortality)|(168hr acute-mortality)|(168 hr acute-mortality)|(one hundred and sixty eight hour acute-mortality)|(one hundred and sixty-eight hour acute-mortality)|(acute-mortality at seven days)|(acute-mortality at 7 days)|(acute-mortality in seven days)|(acute-mortality in 7 days)|(acute-mortality at 168 hours)|(acute-mortality in 168 hours)|(acute-mortality in one hundred and sixty eight hours)|(acute-mortality in one hundred and sixty-eight hours)|(acute-mortality at one hundred and sixty eight hours)|(acute-mortality at one hundred and sixty-eight hours)|(acute fatality sooner than the first week)|(acute fatality sooner than the 1^st^ week)|(acute fatality sooner than the 1^st^ wk)|(case mortality in one hundred and forty four hours)|(case mortality in one hundred and forty-four hours)|(case mortality at one hundred and forty four hours)|(case mortality at one hundred and forty-four hours)|(168 hour case mortality)|(168-hour case mortality)|(168-hr case mortality)|(168hr case mortality)|(168 hr case mortality)|(one hundred and sixty eight hour case mortality)|(one hundred and sixty-eight hour case mortality)|(case mortality at seven days)|(case mortality at 7 days)|(case mortality in seven days)|(case mortality in 7 days)|(case mortality at 168 hours)|(case mortality in 168 hours)|(case mortality in one hundred and sixty eight hours)|(case mortality in one hundred and sixty-eight hours)|(case mortality at one hundred and sixty eight hours)|(case mortality at one hundred and sixty-eight hours)|(acute-fatality sooner than the first week)|(acute-fatality sooner than the 1^st^ week)|(acute-fatality sooner than the 1^st^ wk)|(case-mortality in one hundred and forty four hours)|(case-mortality in one hundred and forty-four hours)|(case-mortality at one hundred and forty four hours)|(case-mortality at one hundred and forty-four hours)|(168 hour case-mortality)|(168-hour case-mortality)|(168-hr case-mortality)|(168hr case-mortality)|(168 hr case-mortality)|(one hundred and sixty eight hour case-mortality)|(one hundred and sixty-eight hour case-mortality)|(case-mortality at seven days)|(case-mortality at 7 days)|(case-mortality in seven days)|(case-mortality in 7 days)|(case-mortality at 168 hours)|(case-mortality in 168 hours)|(case-mortality in one hundred and sixty eight hours)|(case-mortality in one hundred and sixty-eight hours)|(case-mortality at one hundred and sixty eight hours)|(case-mortality at one hundred and sixty-eight hours)|(acute-fatality sooner than the first week)|(acute-fatality sooner than the 1^st^ week)|(acute-fatality sooner than the 1^st^ wk)|(acute mortality in one hundred and forty four hours)|(acute mortality in one hundred and forty-four hours)|(acute mortality at one hundred and forty four hours)|(acute mortality at one hundred and forty-four hours)|(168 hour acute mortality)|(168-hour acute mortality)|(168-hr acute mortality)|(168hr acute mortality)|(168 hr acute mortality)|(one hundred and sixty eight hour acute mortality)|(one hundred and sixty-eight hour acute mortality)|(acute mortality at seven days)|(acute mortality at 7 days)|(acute mortality in seven days)|(acute mortality in 7 days)|(acute mortality at 168 hours)|(acute mortality in 168 hours)|(acute mortality in one hundred and sixty eight hours)|(acute mortality in one hundred and sixty-eight hours)|(acute mortality at one hundred and sixty eight hours)|(acute mortality at one hundred and sixty-eight hours)|(acute-fatality sooner than the first week)|(acute-fatality sooner than the 1^st^ week)|(acute-fatality sooner than the 1^st^ wk)|(acute-mortality in one hundred and forty four hours)|(acute-mortality in one hundred and forty-four hours)|(acute-mortality at one hundred and forty four hours)|(acute-mortality at one hundred and forty-four hours)|(168 hour acute-mortality)|(168-hour acute-mortality)|(168-hr acute-mortality)|(168hr acute-mortality)|(168 hr acute-mortality)|(one hundred and sixty eight hour acute-mortality)|(one hundred and sixty-eight hour acute-mortality)|(acute-mortality at seven days)|(acute-mortality at 7 days)|(acute-mortality in seven days)|(acute-mortality in 7 days)|(acute-mortality at 168 hours)|(acute-mortality in 168 hours)|(acute-mortality in one hundred and sixty eight hours)|(acute-mortality in one hundred and sixty-eight hours)|(acute-mortality at one hundred and sixty eight hours)|(acute-mortality at one hundred and sixty-eight hours)|(acute-fatality sooner than the first week)|(acute-fatality sooner than the 1^st^ week)|(acute-fatality sooner than the 1^st^ wk)
